# Supplementary material for: Pervasive Listeria monocytogenes Is Common in the Norwegian Food System and Is Associated with Increased Prevalence of Stress Survival and Resistance Determinants
Source: Appl Environ Microbiol. 2022 Aug 25;88(18):e00861-22. doi: 10.1128/aem.00861-22 (PMC9499026; doi:10.1128/aem.00861-22)
Supplement: Supplemental file 1 — Supplemental material. Download aem.00861-22-s0001.pdf, PDF file, 3.3 MB [file aem.00861-22-s0001.pdf]

Supplemental Data

Pervasive *Listeria monocytogenes* are common in the Norwegian food system and associated with increased prevalence of stress survival and resistance determinants

Annette Fagerlund, Eva Wagner, Trond Møretrø, Even Heir, Birgitte Moen, Kathrin Rychli, and Solveig Langsrud

Contents

Text S1: Further description of identified genetic determinants of stress survival, resistance and persistence 2
- Plasmids and *repA* replication initiation genes 2
- Cadmium and arsenic resistance determinants 2
- QAC resistance determinants 3
- Biofilm-associated genes *bapL* and *inlL* 4
- Genetic hotspot harboring stress survival islets 5
- *inlA* PMSC mutations 5
- *inlB* deletion mutation in CC5 genomes 6
- Comment 6

Table S1: The 769 *L. monocytogenes* isolates from food industry included in this study 7
Table S2: Number of isolates from each processing plant 24
Table S3: Prevalence and distribution of plasmids in each CC 25
Table S4: BLAST analysis of stress survival and resistance genes 26
Table S5: Pearson's chi-square test: source meat/salmon 30
Table S6: Pearson's chi-square test: persistence and pervasion 31
Table S7: Pearson's chi-square test: source food/clinical/nature 32
Table S8: Plasmids used in BLAST analysis 33
Table S9: Genes encoding RepA used in BLAST analysis 34
Figure S1: Phylogenetic tree and presence of stress survival genes 35
Figure S2: Presence of *bcrABC* and *qacH* in CC9 36
References 37

Text S1: Further description of identified genetic determinants of stress survival, resistance and persistence

Plasmids and *repA* replication initiation genes

Listeria spp. is dominated by *repA*-family theta-replicating plasmids [1]. Six RepA groups (G1-G6) have been identified in plasmids and another five (G7-G11) by BLAST searches [2]. A comprehensive overview of *Listeria* spp. plasmids was recently published by Chmielowska *et al*. [2], and included five complete plasmid sequences previously identified in ST9 isolates from the currently analyzed dataset [3]. The study [2] reported that a plasmid 100% identical to pMF4545 (from MF4545 included in the current study) was identified in *L. monocytogenes* strain 05/08 isolated from mettwurst (a pork sausage) in Poland in 2008, and that RepA proteins in all five ST9 plasmids belong to RepA phylogenetic group G1 [2]. The majority of *Listeria* plasmids are large plasmids >15 kb that harbor *repA* replication systems. Smaller plasmids are less abundant and more divergent, but most are rolling-circle replication (RCR) plasmids [2].

Analysis of the prevalence and distribution of plasmids showed that the theta-replicating plasmids mainly harbored ***repA* G1** and ***repA* G2** replication genes, in addition to the novel *repA* G12 identified in an isolate from food processing environments and one ***repA* G4** gene identified on a 65 kb contig from a clinical isolate (S3 Table). BLAST analysis against the GenBank nr database identified one single other *L. monocytogenes* plasmid carrying ***repA* G12** (pLM-F-19; accession KY613764). Three different sequence variants of *repA* G1 (>99% identical) and four different sequence variants of *repA* G2 (94-98% identity) were identified. As previously observed [4], the *repA* G2 plasmids were on average larger than *repA* G1 plasmids (72 kb vs. 48 kb). Only two CCs, namely CC7 and CC415, harbored isolates with either G1 or G2 *repA* plasmids. Within CC415, the clade containing 15 isolates from salmon factory S6 contained *repA* G2, while the CC415 isolates from meat factories contained *repA* G1. CC3 and CC9 were among the groups harboring *repA* G1 plasmids, while CC8, CC11, and CC121 only contained *repA* G2 plasmids.

Two **small non-*repA* plasmids** were identified among the clinical isolates. The first was from a CC7 isolate from 2014 (ERR2522330), which contained a contig that could be circularized and was 100% identical to the 2776 bp pAUSMDU00000235 plasmid found in a clinical strain in Australia in 2009 [5]. The plasmid encodes a bacteriocin, a bacteriocin immunity protein, and a DUF536 domain-containing protein, but no known plasmid replication system. The second was identified in an ST691 (CC7) isolate from 2013 (ERR2522310), which contained a plasmid that aligned with 99.98% identity over 99.86% of the plasmid/contig lengths with the 4392 bp long plasmid pLMST6 (pLmN12-0935) from a clinical strain belonging to ST403/CC403 isolated in Switzerland in 2012 [6] (only one SNP in an intergenic region was identified plus mismatches within 6 bp of the end of the contig).

Cadmium and arsenic resistance determinants

Previous studies have shown that around 95% of *L. monocytogenes* plasmids confer cadmium resistance, and the most commonly occurring plasmid-borne determinant is the transposon-associated cassette *cadA1C1* [7]. In the current study, a total of 491 genomes containing plasmids were identified. Of these, ***cadA1C1*** was present on contigs identified as plasmids in 465 isolates, ***cadA2C2*** was found on plasmids in nine isolates, and **both *cadA1C1* and *cadA2C2*** were present in the ten CC5 isolates harboring two plasmids each. In total, the prevalence of cadmium resistance determinants in identified plasmids was 98.6%. The only seven identified plasmids not carrying cadmium resistance genes were the two small plasmids found in clinical isolates, the *repA* G4 and *repA* G12 plasmids, and three *repA* G2 plasmids on contigs of length 64-66 Kb present in D052L and D161L (both CC8 from dairy farms), and MF7648 (CC11/ST451 from factory M8). These seven plasmids lacked all queried

stress/resistance genes, except for the *repA* G4 plasmid in ERR2522309 which carried *arsCBADR* and the small plasmid in ERR2522310 which carried *emrC*.

Seven isolates lacking plasmids harbored *cadA1C1* on a chromosomally located Tn*5422* transposon. These were one CC9 isolate from food (MF4562 from M1), two CC31 isolates (one clinical and one from natural environment) and four CC18 isolates from dairy farms.

Another type of genetic element that may harbor cadmium resistance determinants is the ***Listeria* genomic island 2 (LGI2)**, which is a 35 kb chromosomal region containing a large arsenic resistance operon (*arsA1D1R1D2R2A2B1B2*) and either ***cadA4C4*** or ***cadA5C5*** cadmium resistance genes [8-10]. LGI2 harboring these genetic elements was present in 72 of the genomes examined in the current study, with a prevalence of 2% in clinical isolates, 5% in isolates from natural environments, and 8% in the isolates from food industry. A CC21 isolate collected at factory M3 in 1998 carried the *cadA5C5* genes, while the remaining genomes, belonging to CC1 (in 37 of 48 isolates; 77%), CC2 (in all 7 isolates), CC14 (in 22 of 29 isolates; 76%), and CC204 (in all 5 isolates), carried *cadA4C4*. All seven CC2 isolates (from salmon factory S6) and all five CC204 isolates (from natural environments) contained LGI2. Within CC1, LGI2 was present in all genomes from a large clade comprising 37 isolates from salmon processing factories, but not in the six CC1 clinical isolates or in the three isolates from meat slaughter departments. Within CC14, LGI2 was present in the 20 ST14 isolates from salmon processing plants S1, S2, and S3 and in the two ST14 clinical isolates, but not in the two ST14 isolates from natural environments or in the clade consisting of five ST399 isolates (comprising clinical, food, and environmental isolates). LGI2 was present in 30% of the examined lineage I isolates and in 2% of the lineage II isolates, and thus significantly more enriched in lineage I compared to lineage II ($p < 0.001$). Furthermore, it was significantly more enriched in salmon processing compared to in meat processing factories ($p < 0.001$).

A second type of **arsenic resistance cassette (*arsCBADR*)** is carried on a **Tn*554*-like transposon** [8], and was present in 314 of the examined genomes. All genes in the Tn*554 ars* operon were 100% identical between isolates. This transposon and resistance cassette was present in 19% (n=21) of CC7 and 98% (n=287) of CC9 isolates, in addition to one isolate each from CC1, CC21, CC8, and three belonging to CC31. In contrast to the *ars* operon present on LGI2, it was thus significantly associated with isolates belonging to lineage II and with isolates from meat processing plants (Fisher's exact test). The *arsCBADR* operon was located on the chromosome in 282 CC9 and two CC31 genomes, and on plasmids in the remaining 25 genomes, including in all CC7 isolates.

The only isolate containing both arsenic resistance determinants was the CC21 isolate harboring LGI2 with *cadA5C5*. This isolate carried a 102 kb *repA* G2 plasmid harboring *cadA1C1* and *arsCBADR*. None of the isolates harboring LGI2 with *cadA4C4* carried plasmids.

Homologs to the ***cadA3C3*** genes present in *L. monocytogenes* EGD-e, the ***cadA6C6*** genes [11] present on the *Listeria seeligeri* Sr12 plasmid pLIS4 and the *Listeria ivanovii* strain Sr11 plasmid pLIS6 were not identified in the current dataset. The ***cadA7C7*** genes described by Lee *et al*. [12] were >99% identical to the *cadA4C4* genes, and thus not considered a separate genetic determinant in the current work (and all BLAST hits to "*cadA7C7*" genes were more similar to the *cadA4C4* reference than to *cadA7A7*).

QAC resistance determinants

Quaternary ammonium compounds (QACs) are a group of sanitizers commonly used in the food industry. Several membrane-bound efflux transporters belonging to the small multidrug resistance (SMR) protein family have been described to confer resistance to QACs in *L. monocytogenes*. These include **QacH**, encoded on the Tn*6188* transposon [13, 14], **EmrC** encoded on the pLMST6 plasmid [6,

15], and **EmrE** encoded on Listeria Genomic Island 1 (LGI1) [16, 17]. EmrC and EmrE are 70% and 42% identical to QacH encoded on the Tn*6188* transposon, respectively. Another QAC resistance determinant is the ***bcrABC*** resistance cassette encoding a TetR family regulator (BcrA) and two SMR proteins (BcrB and BcrC) [18, 19]. BcrB and BcrC are 42% and 44% identical to QacH, respectively. One study [20] has referred to a SMR resistance gene *ebrB* common in food-derived lineage II strains, identified through gene cluster analysis. However, closer examination revealed that the genes in the identified cluster were identical to *bcrC* and *qacH*.

The Tn*6188* transposon carrying *qacH* was identified in 45% (133/293) of the CC9 genomes and in 86% (88/102) of the CC121 genomes of both food processing and clinical origin, and thus only observed in lineage II. In all cases the Tn*6188* transposon was chromosomally encoded. In addition, the ***repA* G4 plasmid** carried by the CC1 clinical isolate (ERR2522309) **encoded a protein that was 90% identical to QacH** encoded on Tn*6188*. BLAST analysis against the GenBank nr database showed that this *qacH* variant was found in three other *L. monocytogenes* isolates, including on plasmids pLIS26 and pLIS12, on *Clostridioides difficile* plasmid pCD-CDSMR, an *Enterococcus faecalis* isolate, and on 13 *Bacillus cereus* plasmids. The *emrC* gene was present on the small pLMST6 plasmid found in the CC7/ST691 clinical isolate (ERR2522310), while the *emrE* gene was not identified in the current dataset.

The *bcrABC* resistance cassette was identified in 73% (11/15) of the CC5 genomes, in one genome from a clinical CC7 isolate (ERR2522290), and in 43% (127/293) of the CC9 genomes. This resistance cassette was thus found in both lineage I and lineage II in the current dataset but was only present on *repA* G1 and G2 plasmids, never on the chromosome. Interestingly, *bcrABC* and *qacH* were never observed in the same isolate, despite CC9 isolates co-existing in the same environment in both factories M1 and M4 [21] (Fig. S2). All genomes harboring *bcrABC* also carried *cadA2C2* and *tmr*, encoding a triphenylmethane reductase that mediates detoxification of dyes such as crystal violet [22].

Some genes contributing to QAC resistance in *L. monocytogenes* were identified as core genes present in all genomes examined in the current study. These include the ***sug*** operon [23] which shows homology towards *bcrABC*, the ***mdrL*** [24-26] and ***lde*** [27, 28] genes encoding non-specific multidrug efflux MFS transporters, ***fepA*** and ***fepR*** encoding a MATE family efflux pump and its regulator [29] and the ***mepA*** [30] MATE family transporter. No PMSC mutations in *fepR*, described to confer increased QAC resistance [29], were identified in the currently examined genomes.

Biofilm-associated genes *bapL* and *inlL*

Biofilm formation capacity is often considered a stress resistance phenotype and may contribute to the survival and persistence of *L. monocytogenes* in natural and food processing environments [31-33]. Two genes known to be involved in biofilm formation or adhesion in *L. monocytogenes* were identified as accessory genes in the current dataset: *bapL* and *inlL*. **BapL** is a 2013 aa long LPXTG cell-wall anchored protein shown to contribute to surface attachment to abiotic surfaces [34]. The ***inlL*** gene encodes **internalin L**, an 626 or 621 aa long LPXTG protein, has been shown to be involved in adhesion to polystyrene and mucin type 2, as well as biofilm formation [35]. The internalins of *L. monocytogenes* comprise 35 distinct genes encoding proteins with leucine-rich repeat domains [35, 36]. The proteins in this family were initially named internalins due to the involvement of the initially characterized internalins in the infection internalization process. However, none of the other internalins have known roles during internalization.

When present, *bapL* and *inlL* were both always found on the chromosomal backbone, never on plasmid contigs. Both genes were found in all examined CC9, ST20 and CC204 isolates, as well as in the single CC671 isolate. The *bapL* gene was additionally present in the single ST11 and CC475 isolates and all CC14 (both ST14 and ST399), CC90, and CC121 isolates. However, in 84 of the 102 CC121 genomes

(82%), the protein was truncated due to a PMSC mutation at codon number 61, and thus presumed to be non-functional. The *inlL* gene was additionally present in all CC7, CC18, CC19, ST647 (CC20), CC21, CC29, CC37, CC200, CC403, CC412, and CC415 isolates. Thus, both genes were only identified in lineage II genomes, with intact *bapL* present in 68% and *inlL* (both 626 and 621 aa) present in 39% of the examined isolates in this lineage.

Genetic hotspot harboring stress survival islets

A hypervariable genetic hotspot (*lmo0443-lmo0449*) has previously been shown to harbor one of three inserts: stress survival islet 1 (**SSI-1**), **SSI-2**, or LMOf2365_0481. All examined genomes in the current study harbored one of these three genetic elements at genetic location. While no phenotype has been attributed to the presence of LMOf2365_0481 [37], the other islets can promote growth under suboptimal or stress conditions such as low pH and high salt (SSI-1) and alkaline or oxidative stress (SSI-2) [38, 39]. The presence of SSI-1 has also been associated with increased adhesion efficiency and biofilm formation capacity [40-43]. SSI-1 was detected in 60% of the isolates from food processing environments and in a total of 14 CCs, including CC3, CC5, CC7, CC8, and CC9. SSI-2 was detected in CC121 isolates and in the single identified isolate belonging to CC475/ST504 found in the natural environment dataset (MF6841). CC475 and CC121 were more closely related than the majority of CCs, with the CC475 isolate differentiated by 928 wgMLST allelic differences towards the closest CC121 isolate. LMOf2365_0481, with no known associated phenotype, was most prevalent among the isolates from natural environments (75%). All isolates belonging to the same CC harboured the same genetic element in this hypervariable region, indicating that is was relatively stable, although the presence of SSI-1 in both lineage I and II confirms a history of genetic exchange.

inlA PMSC mutations

Internalin A is a 800 aa long LPXTG-anchored cell surface protein composed of a signal peptide sequence, a N-terminal leucine-rich repeat domain, an “inter-repeat” (IR) domain, a B-repeat domain, and a membrane anchor domain. During foodborne infection, one of the pathways by which *L. monocytogenes* can cross the intestinal epithelium to establish a systemic infection is the binding of internalin A to the receptor E-cadherin, followed by transcytosis and finally release of the bacterial cells into the lamina propria [44, 45].

Several different premature stop codon (PMSC) mutations in *inlA* have been described [46]. It has been shown that interruption of either *inlA* or *inlB* by transposon mutagenesis results in increased biofilm formation at 15°C [47]. One of the most common mutation types include mutation type 4, which results in premature termination after codon number 9 and complete loss of internalin A protein. Mutation type 4 is caused by slipped strand mispairing resulting in deletion of one adenine in a seven adenine homopolymeric tract harbored by the *inlA* alleles present in most lineage II isolates, and may thus revert back to the wild-type allele by the same mechanism, constituting a phase variation mechanism. In lineage I isolates this homopolymeric tract is replaced by AAGAAA, accounting for the lack of PMSC type 4 mutations in lineage I isolates [48]. Most other described PMSC mutations occur within the B-repeat or membrane anchor domain regions [46], resulting in the expression of truncated forms of internalin A which are secreted rather than anchored to the bacterial cell wall [49].

The following *inlA* PMSC mutation types [46] were identified in the current study:

***inlA* mutation type**	**Amino acid (aa) position of mutation**	**Isolates where the mutation was identified**
PMSC mutation type 4	aa position 9	All four CC199 isolates, all three ST31 isolates, and two (0.7%) of the CC9 isolates
PMSC mutation type 6	aa position 492	95 (93%) of the CC121 isolates

PMSC mutation type 13	aa position 527	One (0.3%) of the CC9 isolate (MF5639)
PMSC mutation type 12	aa position 576	136 (47%) of the CC9 isolates
PMSC mutation type 1	aa position 606	All 13 CC5 (lineage I) isolates from factory M6
PMSC mutation type 11	aa position 685	152 (52%) of the CC9 isolates

The prevalence of *inlA* PMSC mutations within the examined sets of isolates from Norwegian food processing environments, clinical isolates, and rural and natural environments was 51%, 15%, and 0%, respectively (Fig. 6 in main text). With the exception of the 13 CC5 isolates from factory M6 which harbor *inlA* PMSC mutation type 1 (which has also been identified in CC5 isolates in other studies [43, 50]), all identified isolates with *inlA* PMSC mutations belonged to lineage II. In line with the described phase variation mechanism responsible for the *inlA* PMSC mutation type 4, the isolates harboring this mutation type were polyphyletic, appearing in multiple parts of the phylogenetic tree (CC199, ST31, and CC9). The remaining groups of isolates harboring each *inlA* PMSC mutation belonged to monophyletic groups. As previously observed [51], several different *inlA* PMSC truncation mutations were observed within CC9, representing convergent evolution within this CC.

In addition to *inlA* PMSC mutations, a previously described **3-codon deletion (3CD) *inlA* genotype** was identified, which results in deletion of three amino acids (aspartic acid, threonine, and serine) at positions 738–740 in the pre anchor region [52]. In contrast to the PMSC mutations, isolates harboring the 3CD mutation appear to show increased virulence relative to the wild-type allele [43, 52], and is more prevalent among clinical than food isolates [43]. Interestingly, the 3CD mutation represents another case of convergent evolution within the *inlA* gene [52], and was in the current study identified in the lineage I CCs CC6 (all isolates, with representatives from food industry, clinical, and natural environment datasets) and CC315 (all isolates, which were from the S6 salmon factory from 2017-2020), and in the single lineage II CC89 clinical isolate (ERR2522298). 3CD mutations in CC6, CC315 and CC89 isolates were identified also in other studies [4, 43, 53].

inlB deletion mutation in CC5 genomes

The lineage I CC5 isolates from meat factory M6, which harbor the *inlA* PMSC mutation type 1, additionally carry a **47-codon deletion (47CD)** after codon 356 in ***inlB***. This gene is transcribed immediately downstream of *inlA* and encodes internalin B. Like internalin A, internalin B is a cell surface protein that mediates binding and entry into eukaryotic cells, but the two proteins bind different receptors and therby mediate entry into different cell types [45]. The 47CD mutation results in removal of the B-repeat of internalin B [54, 55] which appears to represent a functional protein-protein interaction site [56], and was previously identified in around 1% of sequenced *L. monocytogenes* isolates [42]. Both *inlA* PMSC mutation type 1 and a 47CD *inlB* allele were detected in eight CC5 isolates from Polish food processing environments [57]. An internal in-frame deletion mutation in *inlB* was also identified in 11 CC5 isolates from another study [58]. In contrast to the CC5 isolates from food processing environments, the two clinical CC5 isolates examined in the current study carried neither the *inlA* PMSC nor the *inlB* 47CD mutations.

Comment

BLAST analyses against the NCBI GenBank nr database was performed 12.03.2022.

Table S1: The 769 *L. monocytogenes* isolates from food industry included in this study

Isolate	Collection Year	Factory	Food sector	Sample type	MLST	CC	Lineage	SNP reference	BioProject*	BioSample	SRA Run	WGS GenBank Accession
**MF3638**	2007	-	dairy	food product (cheese)	ST7	CC7	II	MF2133	PRJNA689484	SAMN17224281	SRR13588325	JAERFU000000000
**MF6990**	2018	-	domestic kitchen	food processing environment	ST9	C9	II	MF1576	PRJNA689484	SAMN17224388	SRR13588259	JAERBU000000000
**MF1332**	1990	-	meat	food processing - low hygienic zone	ST18	CC18	II	MF4566	PRJNA689484	SAMN17224249	SRR13588426	JAERHA000000000
**MF1341**	1990	-	meat	food processing - low hygienic zone	ST177	CC177	II	MF7243	PRJNA689484	SAMN17224250	SRR13588425	JAERGZ000000000
**MF1342**	1990	-	meat	food processing - low hygienic zone	ST1	CC1	I	MF7036	PRJNA689484	SAMN17224251	SRR13588392	JAERGY000000000
**MF2154**	1991	M3	meat	raw material	ST7	CC7	II	MF2133	PRJNA689484	SAMN17224264	SRR13588397	JAERGL000000000
**MF2155**	1991	M3	meat	raw material	ST7	CC7	II	MF2133	PRJNA689484	SAMN17224265	SRR13588396	JAERGK000000000
**MF2184**	1992	-	meat	food product (cooked)	ST3	CC3	I	MF7891	PRJNA689484	SAMN17224276	SRR13588326	JAERFZ000000000
**MF1548**	1998	M3	meat	food processing environment	ST21	CC21	II	MF7744	PRJNA689484	SAMN17224252	SRR13588381	JAERGX000000000
**MF1576**	1998	M3	meat	food processing environment	ST9	CC9	II	MF1576	PRJNA689484	SAMN17224253	SRR13588370	JAERGW000000000
**MF1642**	1998	M3	meat	food processing environment	ST9	CC9	II	MF1576	PRJNA689484	SAMN17224254	SRR13588359	JAERGV000000000
**MF2175**	1998	M3	meat	raw meat - during processing	ST9	CC9	II	MF1576	PRJNA689484	SAMN17224268	SRR13588394	JAERGH000000000
**MF2179**	1998	M3	meat	raw meat - during processing	ST121	CC121	II	MF4804	PRJNA689484	SAMN17224271	SRR13588390	JAERGE000000000
**MF2183**	1998	M3	meat	food processing environment	ST9	CC9	II	MF1576	PRJNA689484	SAMN17224275	SRR13588386	JAERGA000000000
**MF2177**	2001	M3	meat	food processing environment	ST7	CC7	II	MF2133	PRJNA689484	SAMN17224269	SRR13588393	JAERGG000000000
**MF2178**	2001	M3	meat	food processing environment	ST9	CC9	II	MF1576	PRJNA689484	SAMN17224270	SRR13588391	JAERGF000000000
**MF2180**	2001	M3	meat	food processing environment	ST121	CC121	II	MF4804	PRJNA689484	SAMN17224272	SRR13588389	JAERGD000000000
**MF2181**	2001	M3	meat	food processing environment	ST1	CC1	I	MF7036	PRJNA689484	SAMN17224273	SRR13588388	JAERGC000000000
**MF2182**	2001	M3	meat	food processing environment	ST1	CC1	I	MF7036	PRJNA689484	SAMN17224274	SRR13588387	JAERGB000000000
**MF2196**	2001	M3	meat	food processing environment	ST9	CC9	II	MF1576	PRJNA689484	SAMN17224277	SRR13588385	JAERFY000000000
**MF2197**	2001	M3	meat	food processing environment	ST9	CC9	II	MF1576	PRJNA689484	SAMN17224278	SRR13588384	JAERFX000000000
**MF2198**	2001	M3	meat	food processing environment	ST9	CC9	II	MF1576	PRJNA689484	SAMN17224279	SRR13378930	JAERFW000000000
**MF2199**	2001	M3	meat	raw meat - during processing	ST7	CC7	II	MF2133	PRJNA689484	SAMN17224280	SRR13588383	JAERFV000000000
**MF2162**	2002	-	meat	food processing environment	ST7	CC7	II	MF2133	PRJNA689484	SAMN17224266	SRR13588395	JAERGJ000000000
**MF2174**	2002	M3	meat	raw meat - during processing	ST200	CC200	I		PRJNA689484	SAMN17224267	SRR13378931	JAERGI000000000
**MF2133**	2004	M6	meat	food processing environment	ST7	CC7	II	MF2133	PRJNA689484	SAMN17224255	SRR13588348	JAERGU000000000
**MF2134**	2004	M6	meat	food product (cooked)	ST7	CC7	II	MF2133	PRJNA689484	SAMN17224256	SRR13588337	JAERGT000000000
**MF2135**	2004	M6	meat	food processing environment	ST8	CC8	II	MF4245	PRJNA689484	SAMN17224257	SRR13588448	JAERGS000000000
**MF2136**	2004	M6	meat	food processing environment	ST121	CC121	II	MF4804	PRJNA689484	SAMN17224258	SRR13588437	JAERGR000000000
**MF2137**	2004	M6	meat	food processing environment	ST19	CC19	II	MF7335	PRJNA689484	SAMN17224259	SRR13588424	JAERGQ000000000
**MF2138**	2004	M6	meat	food processing environment	ST7	CC7	II	MF2133	PRJNA689484	SAMN17224260	SRR13588413	JAERGP000000000
**MF2139**	2004	M6	meat	food processing environment	ST7	CC7	II	MF2133	PRJNA689484	SAMN17224261	SRR13588402	JAERGO000000000
**MF2140**	2004	M6	meat	food product (cooked)	ST7	CC7	II	MF2133	PRJNA689484	SAMN17224262	SRR13588399	JAERGN000000000
**MF2141**	2004	M6	meat	food product (cooked)	ST121	CC121	II	MF4804	PRJNA689484	SAMN17224263	SRR13588398	JAERGM000000000
**MF7799**	2004	M6	meat	food product (cooked)	ST121	CC121	II	MF4804	PRJNA689484	SAMN17224624	SRR13588423	JAEQSS000000000
**MF5367**	2006	M6	meat	food processing environment	ST7	CC7	II	MF2133	PRJNA689484	SAMN17224328	SRR13588161	JAERDZ000000000
**MF4999**	2009	M1	meat	food processing environment	ST9	CC9	II	MF1576	PRJNA419519	SAMN14314731	SRR11262207	JAFFES000000000
**MF6174**	2009	M1	meat	food processing environment	ST9	CC9	II	MF1576	PRJNA419519	SAMN14314769	SRR11262036	JAFFDG000000000
**MF5000**	2010	M1	meat	food processing environment	ST9	CC9	II	MF1576	PRJNA419519	SAMN14314732	SRR11262206	JAFFER000000000
**MF5368**	2010	M6	meat	food processing environment	ST5	CC5	I	MF7680	PRJNA689484	SAMN17224329	SRR13588375	JAERDY000000000
**MF6175**	2010	M1	meat	food processing environment	ST9	CC9	II	MF1576	PRJNA419519	SAMN14314770	SRR11262035	JAFFDF000000000
**MF6186**	2010	M6	meat	food processing environment	ST5	CC5	I	MF7680	PRJNA689484	SAMN17224344	SRR13588368	JAERDJ000000000
**MF5001**	2011	M1	meat	food processing environment	ST9	CC9	II	MF1576	PRJNA419519	SAMN14314733	SRR11262205	JAFFEQ000000000

**MF5369**	2011	M6	meat	food processing environment	ST8	CC8	II	MF4245	PRJNA293674	SAMN04009323	SRR3099224	LKUY00000000
**MF5370**	2011	M6	meat	food processing environment	ST7	CC7	II	MF2133	PRJNA689484	SAMN17224330	SRR13588374	JAERDX000000000
**MF6176**	2011	M1	meat	food processing environment	ST9	CC9	II	MF1576	PRJNA419519	SAMN14314771	SRR11262034	JAFFDE000000000
**MF6177**	2011	M1	meat	food processing environment	ST9	CC9	II	MF1576	PRJNA419519	SAMN14314772	SRR11262033	JAFFDD000000000
**MF6178**	2011	M1	meat	food processing environment	ST9	CC9	II	MF1576	PRJNA419519	SAMN14314773	SRR11262031	JAFFDC000000000
**MF6179**	2011	M1	meat	food processing environment	ST9	CC9	II	MF1576	PRJNA419519	SAMN14314774	SRR11262032	JAFFDB000000000
**MF6187**	2011	M6	meat	food processing environment	ST7	CC7	II	MF2133	PRJNA689484	SAMN17224345	SRR13588158	JAERDI000000000
**MF6188**	2011	M6	meat	food processing environment	ST7	CC7	II	MF2133	PRJNA689484	SAMN17224346	SRR13588157	JAERDH000000000
**MF6189**	2011	M6	meat	food processing environment	ST7	CC7	II	MF2133	PRJNA689484	SAMN17224347	SRR13588156	JAERDG000000000
**MF6190**	2011	M6	meat	food processing environment	ST7	CC7	II	MF2133	PRJNA689484	SAMN17224348	SRR13588155	JAERDF000000000
**MF6191**	2011	M6	meat	food processing environment	ST7	CC7	II	MF2133	PRJNA689484	SAMN17224349	SRR13588154	JAERDE000000000
**MF6192**	2011	M6	meat	food processing environment	ST7	CC7	II	MF2133	PRJNA689484	SAMN17224350	SRR13588152	JAERDD000000000
**MF6193**	2011	M6	meat	food processing environment	ST7	CC7	II	MF2133	PRJNA689484	SAMN17224351	SRR13588151	JAERDC000000000
**MF4536**	2012	M1	meat	food processing environment	ST9	CC9	II	MF1576	PRJNA419519	SAMN14314680	SRR11262219	JAFFGR000000000
**MF4537**	2012	M1	meat	food processing environment	ST9	CC9	II	MF1576	PRJNA419519	SAMN14314681	SRR11262218	JAFFGQ000000000
**MF4538**	2012	M1	meat	food processing environment	ST9	CC9	II	MF1576	PRJNA419519	SAMN14314682	SRR11262108	JAFFGP000000000
**MF4539**	2012	M1	meat	food processing environment	ST9	CC9	II	MF1576	PRJNA419519	SAMN14314683	SRR11262173	JAFFGO000000000
**MF4540**	2012	M1	meat	food processing environment	ST9	CC9	II	MF1576	PRJNA419519	SAMN14314684	SRR11262162	JAFFGN000000000
**MF4541**	2012	M1	meat	food processing environment	ST9	CC9	II	MF1576	PRJNA419519	SAMN14314685	SRR11262214	JAFFGM000000000
**MF4542**	2012	M1	meat	food processing environment	ST9	CC9	II	MF1576	PRJNA419519	SAMN14314686	SRR11262203	JAFFGL000000000
**MF4543**	2012	M1	meat	food processing environment	ST9	CC9	II	MF1576	PRJNA419519	SAMN14314687	SRR11262192	JAFFGK000000000
**MF4544**	2012	M1	meat	food processing environment	ST9	CC9	II	MF1576	PRJNA419519	SAMN14314688	SRR11262053	JAFFGJ000000000
**MF4545**	2012	M1	meat	food processing environment	ST9	CC9	II	MF1576	PRJNA419519	SAMN08056483	SRR11262042	CP025443, CP025444
**MF4546**	2012	M1	meat	food processing environment	ST9	CC9	II	MF1576	PRJNA419519	SAMN14314689	SRR11262217	JAFFGI000000000
**MF4547**	2012	M1	meat	food processing environment	ST9	CC9	II	MF1576	PRJNA419519	SAMN14314690	SRR11262021	JAFFGH000000000
**MF4549**	2012	M1	meat	food processing environment	ST9	CC9	II	MF1576	PRJNA419519	SAMN14314691	SRR11262010	JAFFGG000000000
**MF4550**	2012	M1	meat	food processing environment	ST9	CC9	II	MF1576	PRJNA419519	SAMN14314692	SRR11262154	JAFFGF000000000
**MF4551**	2012	M1	meat	food processing environment	ST9	CC9	II	MF1576	PRJNA419519	SAMN14314693	SRR11262142	JAFFGE000000000
**MF4552**	2012	M1	meat	food processing environment	ST9	CC9	II	MF1576	PRJNA419519	SAMN14314694	SRR11262131	JAFFGD000000000
**MF4553**	2012	M1	meat	food processing environment	ST9	CC9	II	MF1576	PRJNA419519	SAMN14314695	SRR11261998	JAFFGC000000000
**MF4554**	2012	M1	meat	food processing environment	ST9	CC9	II	MF1576	PRJNA419519	SAMN14314696	SRR11261987	JAFFGB000000000
**MF4555**	2012	M1	meat	food processing environment	ST9	CC9	II	MF1576	PRJNA419519	SAMN14314697	SRR11261976	JAFFGA000000000
**MF4556**	2012	M1	meat	food processing environment	ST9	CC9	II	MF1576	PRJNA419519	SAMN14314698	SRR11262119	JAFFFZ000000000
**MF4557**	2012	M1	meat	food processing environment	ST9	CC9	II	MF1576	PRJNA419519	SAMN14314699	SRR11262107	JAFFFY000000000
**MF4558**	2012	M1	meat	food processing environment	ST9	CC9	II	MF1576	PRJNA419519	SAMN14314700	SRR11262096	JAFFFX000000000
**MF4559**	2012	M1	meat	food processing environment	ST9	CC9	II	MF1576	PRJNA419519	SAMN14314701	SRR11262085	JAFFFW000000000
**MF4560**	2012	M1	meat	food processing environment	ST9	CC9	II	MF1576	PRJNA419519	SAMN14314702	SRR11262074	JAFFFV000000000
**MF4561**	2012	M1	meat	food processing environment	ST9	CC9	II	MF1576	PRJNA419519	SAMN14314703	SRR11262063	JAFFFU000000000
**MF4562**	2012	M1	meat	food processing environment	ST9	CC9	II	MF1576	PRJNA419519	SAMN08056484	SRR11262180	CP025442
**MF4563**	2012	M1	meat	food processing environment	ST9	CC9	II	MF1576	PRJNA419519	SAMN14314704	SRR11262177	JAFFFT000000000
**MF4564**	2012	M1	meat	food processing environment	ST91	CC91	II	MF7663	PRJNA689484	SAMN17224307	SRR13588199	JAEREU000000000
**MF4565**	2012	M1	meat	food processing environment	ST18	CC18	II	MF4566	PRJNA689484	SAMN17224308	SRR13588324	JAERET000000000
**MF4566**	2012	M1	meat	food processing environment	ST18	CC18	II	MF4566	PRJNA689484	SAMN17224309	SRR13378883	JAERES000000000
**MF4624**	2012	M4	meat	food processing environment	ST9	CC9	II	MF1576	PRJNA419519	SAMN08056485	SRR11262176	CP025259, CP025260
**MF4625**	2012	M4	meat	food processing environment	ST9	CC9	II	MF1576	PRJNA419519	SAMN14314705	SRR11262175	JAFFFS000000000
**MF4626**	2012	M4	meat	food processing environment	ST9	CC9	II	MF1576	PRJNA419519	SAMN08056486	SRR11262174	CP025082, CP025083

**MF4627**	2012	M4	meat	food processing environment	ST451	CC11	II	MF4627	PRJNA689484	SAMN17224314	SRR13588288	JAEREN000000000
**MF4629**	2012	M4	meat	food processing environment	ST9	CC9	II	MF1576	PRJNA419519	SAMN14314706	SRR11262172	JAFFFR000000000
**MF4676**	2012	M4	meat	food processing environment	ST9	CC9	II	MF1576	PRJNA419519	SAMN14314707	SRR11262171	JAFFFQ000000000
**MF4677**	2012	M4	meat	food processing environment	ST9	CC9	II	MF1576	PRJNA419519	SAMN14314708	SRR11262170	JAFFFP000000000
**MF4678**	2012	M4	meat	food processing environment	ST9	CC9	II	MF1576	PRJNA419519	SAMN14314709	SRR11262169	JAFFFO000000000
**MF4679**	2012	M4	meat	food processing environment	ST9	CC9	II	MF1576	PRJNA419519	SAMN14314710	SRR11262168	JAFFFN000000000
**MF4680**	2012	M4	meat	food processing environment	ST9	CC9	II	MF1576	PRJNA419519	SAMN14314711	SRR11262167	JAFFFM000000000
**MF4681**	2012	M4	meat	food processing environment	ST9	CC9	II	MF1576	PRJNA419519	SAMN14314712	SRR11262166	JAFFFL000000000
**MF4682**	2012	M4	meat	food processing environment	ST9	CC9	II	MF1576	PRJNA419519	SAMN14314713	SRR11262165	JAFFFK000000000
**MF4685**	2012	M4	meat	food processing environment	ST9	CC9	II	MF1576	PRJNA419519	SAMN14314714	SRR11262164	JAFFFJ000000000
**MF4686**	2012	M4	meat	food processing environment	ST9	CC9	II	MF1576	PRJNA419519	SAMN14314715	SRR11262163	JAFFFI000000000
**MF4687**	2012	M4	meat	food processing environment	ST9	CC9	II	MF1576	PRJNA419519	SAMN14314716	SRR11262161	JAFFFH000000000
**MF4688**	2012	M4	meat	food processing environment	ST9	CC9	II	MF1576	PRJNA419519	SAMN14314717	SRR11262160	JAFFFG000000000
**MF4689**	2012	M4	meat	food processing environment	ST9	CC9	II	MF1576	PRJNA419519	SAMN14314718	SRR11262159	JAFFFF000000000
**MF4690**	2012	M4	meat	food processing environment	ST9	CC9	II	MF1576	PRJNA419519	SAMN14314719	SRR11262158	JAFFFE000000000
**MF4691**	2012	M4	meat	food processing environment	ST9	CC9	II	MF1576	PRJNA419519	SAMN14314720	SRR11262157	JAFFFD000000000
**MF4692**	2012	M4	meat	food processing environment	ST9	CC9	II	MF1576	PRJNA419519	SAMN14314721	SRR11262156	JAFFFC000000000
**MF4693**	2012	M4	meat	food processing environment	ST9	CC9	II	MF1576	PRJNA419519	SAMN14314722	SRR11262155	JAFFFB000000000
**MF4694**	2012	M4	meat	food processing environment	ST9	CC9	II	MF1576	PRJNA419519	SAMN14314723	SRR11262153	JAFFFA000000000
**MF4695**	2012	M4	meat	food processing environment	ST9	CC9	II	MF1576	PRJNA419519	SAMN14314724	SRR11262216	JAFFEZ000000000
**MF4696**	2012	M4	meat	food processing environment	ST9	CC9	II	MF1576	PRJNA419519	SAMN14314725	SRR11262215	JAFFEY000000000
**MF4697**	2012	M4	meat	food processing environment	ST9	CC9	II	MF1576	PRJNA419519	SAMN08056487	SRR11262213	CP025438, CP025439
**MF4712**	2012	M4	meat	meat - during processing	ST7	CC7	II	MF2133	PRJNA689484	SAMN17224315	SRR13588282	JAEREM000000000
**MF4989**	2012	M4	meat	food processing environment	ST451	CC11	II	MF4627	PRJNA689484	SAMN17224318	SRR13588377	JAEREJ000000000
**MF4991**	2012	M2	meat	food processing environment	ST7	CC7	II	MF2133	PRJNA689484	SAMN17224319	SRR13588168	JAEREI000000000
**MF4995**	2012	M1	meat	food processing environment	ST9	CC9	II	MF1576	PRJNA419519	SAMN14314727	SRR11262211	JAFFEW000000000
**MF4996**	2012	M1	meat	food processing environment	ST9	CC9	II	MF1576	PRJNA419519	SAMN14314728	SRR11262210	JAFFEV000000000
**MF4997**	2012	M1	meat	food processing environment	ST9	CC9	II	MF1576	PRJNA419519	SAMN14314729	SRR11262209	JAFFEU000000000
**MF4998**	2012	M1	meat	food processing environment	ST9	CC9	II	MF1576	PRJNA419519	SAMN14314730	SRR11262208	JAFFET000000000
**MF5366**	2012	M6	meat	food processing environment	ST7	CC7	II	MF2133	PRJNA689484	SAMN17224327	SRR13588278	JAEREA000000000
**MF5371**	2012	M6	meat	food processing environment	ST5	CC5	I	MF7680	PRJNA689484	SAMN17224331	SRR13588373	JAERDW000000000
**MF6152**	2012	M4	meat	food processing environment	ST9	CC9	II	MF1576	PRJNA419519	SAMN14314751	SRR11262057	JAFFDY000000000
**MF6153**	2012	M4	meat	food processing environment	ST9	CC9	II	MF1576	PRJNA419519	SAMN14314752	SRR11262056	JAFFDX000000000
**MF6154**	2012	M4	meat	food processing environment	ST9	CC9	II	MF1576	PRJNA419519	SAMN14314753	SRR11262055	JAFFDW000000000
**MF6155**	2012	M4	meat	food processing environment	ST9	CC9	II	MF1576	PRJNA419519	SAMN14314754	SRR11262054	JAFFDV000000000
**MF6156**	2012	M4	meat	food processing environment	ST9	CC9	II	MF1576	PRJNA419519	SAMN14314755	SRR11262052	JAFFDU000000000
**MF6157**	2012	M4	meat	food processing environment	ST9	CC9	II	MF1576	PRJNA419519	SAMN14314756	SRR11262051	JAFFDT000000000
**MF6158**	2012	M4	meat	food processing environment	ST9	CC9	II	MF1576	PRJNA419519	SAMN14314757	SRR11262050	JAFFDS000000000
**MF6159**	2012	M4	meat	food processing environment	ST9	CC9	II	MF1576	PRJNA419519	SAMN14314758	SRR11262049	JAFFDR000000000
**MF6160**	2012	M4	meat	food processing environment	ST9	CC9	II	MF1576	PRJNA419519	SAMN14314759	SRR11262048	JAFFDQ000000000
**MF6161**	2012	M4	meat	food processing environment	ST9	CC9	II	MF1576	PRJNA419519	SAMN14314760	SRR11262047	JAFFDP000000000
**MF6162**	2012	M4	meat	food processing environment	ST9	CC9	II	MF1576	PRJNA419519	SAMN14314761	SRR11262046	JAFFDO000000000
**MF6163**	2012	M4	meat	food processing environment	ST1416	CC19	II	MF7335	PRJNA689484	SAMN17224342	SRR13378872	JAERDL000000000
**MF6164**	2012	M4	meat	food processing environment	ST1416	CC19	II	MF7335	PRJNA689484	SAMN17224343	SRR13378861	JAERDK000000000
**MF6172**	2012	M1	meat	food processing environment	ST9	CC9	II	MF1576	PRJNA419519	SAMN08056488	SRR11262038	CP025440, CP025441
**MF6173**	2012	M1	meat	food processing environment	ST9	CC9	II	MF1576	PRJNA419519	SAMN14314768	SRR11262037	JAFFDH000000000

**MF6180**	2012	M1	meat	food processing environment	ST9	CC9	II	MF1576	PRJNA419519	SAMN14314775	SRR11262030	JAFFDA000000000
**MF6181**	2012	M1	meat	food processing environment	ST9	CC9	II	MF1576	PRJNA419519	SAMN14314776	SRR11262029	JAFFCZ000000000
**MF6182**	2012	M1	meat	food processing environment	ST9	CC9	II	MF1576	PRJNA419519	SAMN14314777	SRR11262028	JAFFCY000000000
**MF6183**	2012	M1	meat	food processing environment	ST9	CC9	II	MF1576	PRJNA419519	SAMN14314778	SRR11262027	JAFFCX000000000
**MF6184**	2012	M1	meat	food processing environment	ST9	CC9	II	MF1576	PRJNA419519	SAMN14314779	SRR11262026	JAFFCW000000000
**MF6185**	2012	M1	meat	food processing environment	ST9	CC9	II	MF1576	PRJNA419519	SAMN14314780	SRR11262025	JAFFCV000000000
**MF5372**	2013	M5	meat	food processing environment	ST9	CC9	II	MF1576	PRJNA419519	SAMN14314734	SRR11262204	JAFFEP000000000
**MF5376**	2013	M2	meat	food processing environment	ST7	CC7	II	MF2133	PRJNA689484	SAMN17224332	SRR13588277	JAERDV000000000
**MF5377**	2013	M2	meat	food processing environment	ST8	CC8	II	MF4245	PRJNA293674	SAMN04009324	SRR3099225	LKUX00000000
**MF5378**	2013	M2	meat	food processing environment	ST394	CC415	II	MF7713	PRJNA689484	SAMN17224333	SRR13588275	JAERDU000000000
**MF5379**	2013	M4	meat	food processing environment	ST9	CC9	II	MF1576	PRJNA419519	SAMN14314735	SRR11262202	JAFFEO000000000
**MF5380**	2013	M4	meat	food processing environment	ST9	CC9	II	MF1576	PRJNA419519	SAMN14314736	SRR11262201	JAFFEN000000000
**MF5383**	2013	M1	meat	food processing environment	ST9	CC9	II	MF1576	PRJNA419519	SAMN14314737	SRR11262200	JAFFEM000000000
**MF5384**	2013	M1	meat	food processing environment	ST9	CC9	II	MF1576	PRJNA419519	SAMN14314738	SRR11262199	JAFFEL000000000
**MF5385**	2013	M1	meat	food processing environment	ST91	CC91	II	MF7663	PRJNA689484	SAMN17224334	SRR13588274	JAERDT000000000
**MF5386**	2013	M1	meat	food processing environment	ST9	CC9	II	MF1576	PRJNA419519	SAMN14314739	SRR11262198	JAFFEK000000000
**MF5406**	2013	M4	meat	food processing environment	ST9	CC9	II	MF1576	PRJNA419519	SAMN14314740	SRR11262197	JAFFEJ000000000
**MF5628**	2013	M5	meat	food processing environment	ST9	CC9	II	MF1576	PRJNA419519	SAMN14314741	SRR11262196	JAFFEI000000000
**MF6166**	2013	M4	meat	food processing environment	ST9	CC9	II	MF1576	PRJNA419519	SAMN14314762	SRR11262045	JAFFDN000000000
**MF6167**	2013	M4	meat	food processing environment	ST9	CC9	II	MF1576	PRJNA419519	SAMN14314763	SRR11262044	JAFFDM000000000
**MF6168**	2013	M4	meat	food processing environment	ST9	CC9	II	MF1576	PRJNA419519	SAMN14314764	SRR11262043	JAFFDL000000000
**MF6169**	2013	M4	meat	food processing environment	ST9	CC9	II	MF1576	PRJNA419519	SAMN14314765	SRR11262041	JAFFDK000000000
**MF6170**	2013	M4	meat	food processing environment	ST9	CC9	II	MF1576	PRJNA419519	SAMN14314766	SRR11262040	JAFFDJ000000000
**MF6171**	2013	M5	meat	food processing environment	ST9	CC9	II	MF1576	PRJNA419519	SAMN14314767	SRR11262039	JAFFDI000000000
**MF6195**	2013	M5	meat	food processing environment	ST9	CC9	II	MF1576	PRJNA419519	SAMN14314781	SRR11262024	JAFFCU000000000
**MF6196**	2013	M2	meat	food processing environment	ST7	CC7	II	MF2133	PRJNA689484	SAMN17224352	SRR13588150	JAERDB000000000
**MF6197**	2013	M2	meat	food processing environment	ST7	CC7	II	MF2133	PRJNA689484	SAMN17224353	SRR13588271	JAERDA000000000
**MF6198**	2013	M2	meat	food processing environment	ST7	CC7	II	MF2133	PRJNA689484	SAMN17224354	SRR13588149	JAERCZ000000000
**MF6200**	2013	M2	meat	food processing environment	ST7	CC7	II	MF2133	PRJNA689484	SAMN17224355	SRR13588148	JAERCY000000000
**MF6201**	2013	M4	meat	food processing environment	ST9	CC9	II	MF1576	PRJNA419519	SAMN14314782	SRR11262023	JAFFCT000000000
**MF6203**	2013	M4	meat	food processing environment	ST9	CC9	II	MF1576	PRJNA419519	SAMN14314783	SRR11262022	JAFFCS000000000
**MF6204**	2013	M4	meat	food processing environment	ST9	CC9	II	MF1576	PRJNA419519	SAMN14314784	SRR11262020	JAFFCR000000000
**MF6205**	2013	M4	meat	food processing environment	ST9	CC9	II	MF1576	PRJNA419519	SAMN14314785	SRR11262019	JAFFCQ000000000
**MF6206**	2013	M4	meat	food processing environment	ST9	CC9	II	MF1576	PRJNA419519	SAMN14314786	SRR11262018	JAFFCP000000000
**MF6207**	2013	M4	meat	food processing environment	ST9	CC9	II	MF1576	PRJNA419519	SAMN14314787	SRR11262017	JAFFCO000000000
**MF6208**	2013	M4	meat	food processing environment	ST9	CC9	II	MF1576	PRJNA419519	SAMN14314788	SRR11262016	JAFFCN000000000
**MF6209**	2013	M4	meat	food processing environment	ST9	CC9	II	MF1576	PRJNA419519	SAMN14314789	SRR11262015	JAFFCM000000000
**MF6210**	2013	M4	meat	food processing environment	ST9	CC9	II	MF1576	PRJNA419519	SAMN14314790	SRR11262014	JAFFCL000000000
**MF6211**	2013	M1	meat	food processing environment	ST9	CC9	II	MF1576	PRJNA419519	SAMN14314791	SRR11262013	JAFFCK000000000
**MF6212**	2013	M1	meat	food processing environment	ST9	CC9	II	MF1576	PRJNA419519	SAMN14314792	SRR11262012	JAFFCJ000000000
**MF6213**	2013	M1	meat	food processing environment	ST9	CC9	II	MF1576	PRJNA419519	SAMN14314793	SRR11262011	JAFFCI000000000
**MF6214**	2013	M1	meat	food processing environment	ST9	CC9	II	MF1576	PRJNA419519	SAMN14314794	SRR11262009	JAFFCH000000000
**MF6215**	2013	M1	meat	food processing environment	ST9	CC9	II	MF1576	PRJNA419519	SAMN14314795	SRR11262008	JAFFCG000000000
**MF6216**	2013	M1	meat	food processing environment	ST9	CC9	II	MF1576	PRJNA419519	SAMN14314796	SRR11262007	JAFFCF000000000
**MF6217**	2013	M1	meat	food processing environment	ST9	CC9	II	MF1576	PRJNA419519	SAMN14314797	SRR11262006	JAFFCE000000000
**MF6218**	2013	M1	meat	food processing environment	ST91	CC91	II	MF7663	PRJNA689484	SAMN17224356	SRR13588367	JAERCX000000000

**MF6219**	2013	M1	meat	food processing environment	ST9	CC9	II	MF1576	PRJNA419519	SAMN14314798	SRR11262005	JAFFCD000000000
**MF6220**	2013	M1	meat	food processing environment	ST9	CC9	II	MF1576	PRJNA419519	SAMN14314799	SRR11262004	JAFFCC000000000
**MF6221**	2013	M1	meat	food processing environment	ST9	CC9	II	MF1576	PRJNA419519	SAMN14314800	SRR11262003	JAFFCB000000000
**MF4994**	2014	M4	meat	food processing environment	ST9	CC9	II	MF1576	PRJNA419519	SAMN14314726	SRR11262212	JAFFEX000000000
**MF5630**	2014	M1	meat	food processing environment	ST19	CC19	II	MF7335	PRJNA689484	SAMN17224337	SRR13588273	JAERDQ000000000
**MF5633**	2014	M1	meat	food processing environment	ST9	CC9	II	MF1576	PRJNA419519	SAMN14314742	SRR11262195	JAFFEH000000000
**MF5634**	2014	M4	meat	food processing environment	ST121	CC121	II	MF4804	PRJNA689484	SAMN17224338	SRR13588272	JAERDP000000000
**MF5635**	2014	M1	meat	food processing environment	ST9	CC9	II	MF1576	PRJNA419519	SAMN14314743	SRR11262194	JAFFEG000000000
**MF5639**	2014	M4	meat	food processing environment	ST9	CC9	II	MF1576	PRJNA419519	SAMN14314744	SRR11262193	JAFFEF000000000
**MF5641**	2014	M4	meat	food processing environment	ST451	CC11	II	MF4627	PRJNA689484	SAMN17224339	SRR13588371	JAERDO000000000
**MF5642**	2014	M4	meat	food processing environment	ST9	CC9	II	MF1576	PRJNA419519	SAMN14314745	SRR11262191	JAFFEE000000000
**MF5645**	2014	M1	meat	food processing environment	ST9	CC9	II	MF1576	PRJNA419519	SAMN14314746	SRR11262190	JAFFED000000000
**MF5646**	2014	M1	meat	food processing environment	ST91	CC91	II	MF7663	PRJNA689484	SAMN17224340	SRR13588369	JAERDN000000000
**MF5647**	2014	M2	meat	food processing environment	ST7	CC7	II	MF2133	PRJNA689484	SAMN17224341	SRR13588159	JAERDM000000000
**MF5648**	2014	M4	meat	food product (cooked)	ST9	CC9	II	MF1576	PRJNA419519	SAMN14314747	SRR11262189	JAFFEC000000000
**MF5649**	2014	M4	meat	food processing environment	ST9	CC9	II	MF1576	PRJNA419519	SAMN14314748	SRR11262188	JAFFEB000000000
**MF5653**	2014	M4	meat	food processing environment	ST9	CC9	II	MF1576	PRJNA419519	SAMN14314749	SRR11262187	JAFFEA000000000
**MF5655**	2014	M4	meat	food product (cooked)	ST9	CC9	II	MF1576	PRJNA419519	SAMN14314750	SRR11262186	JAFFDZ000000000
**MF6222**	2014	M4	meat	food processing environment	ST9	CC9	II	MF1576	PRJNA419519	SAMN14314801	SRR11262002	JAFFCA000000000
**MF6223**	2014	M4	meat	food processing environment	ST9	CC9	II	MF1576	PRJNA419519	SAMN14314802	SRR11262001	JAFFBZ000000000
**MF6224**	2014	M4	meat	food processing environment	ST9	CC9	II	MF1576	PRJNA419519	SAMN14314803	SRR11262000	JAFFBY000000000
**MF6225**	2014	M4	meat	food processing environment	ST9	CC9	II	MF1576	PRJNA419519	SAMN14314804	SRR11262152	JAFFBX000000000
**MF6226**	2014	M4	meat	food processing environment	ST9	CC9	II	MF1576	PRJNA419519	SAMN14314805	SRR11262151	JAFFBW000000000
**MF6227**	2014	M4	meat	food processing environment	ST9	CC9	II	MF1576	PRJNA419519	SAMN14314806	SRR11262150	JAFFBV000000000
**MF6228**	2014	M4	meat	food processing environment	ST9	CC9	II	MF1576	PRJNA419519	SAMN14314807	SRR11262149	JAFFBU000000000
**MF6229**	2014	M4	meat	food processing environment	ST9	CC9	II	MF1576	PRJNA419519	SAMN14314808	SRR11262148	JAFFBT000000000
**MF6234**	2014	M4	meat	food processing environment	ST9	CC9	II	MF1576	PRJNA419519	SAMN14314809	SRR11262147	JAFFBS000000000
**MF6235**	2014	M4	meat	food processing environment	ST9	CC9	II	MF1576	PRJNA419519	SAMN14314810	SRR11262146	JAFFBR000000000
**MF6236**	2014	M4	meat	food processing environment	ST9	CC9	II	MF1576	PRJNA419519	SAMN14314811	SRR11262145	JAFFBQ000000000
**MF6237**	2014	M4	meat	food processing environment	ST9	CC9	II	MF1576	PRJNA419519	SAMN14314812	SRR11262144	JAFFBP000000000
**MF6238**	2014	M4	meat	food processing environment	ST9	CC9	II	MF1576	PRJNA419519	SAMN14314813	SRR11262143	JAFFBO000000000
**MF6239**	2014	M4	meat	food processing environment	ST9	CC9	II	MF1576	PRJNA419519	SAMN14314814	SRR11262141	JAFFBN000000000
**MF6240**	2014	M1	meat	food processing environment	ST9	CC9	II	MF1576	PRJNA419519	SAMN14314815	SRR11262140	JAFFBM000000000
**MF6241**	2014	M1	meat	food processing environment	ST9	CC9	II	MF1576	PRJNA419519	SAMN14314816	SRR11262139	JAFFBL000000000
**MF6242**	2014	M1	meat	food processing environment	ST9	CC9	II	MF1576	PRJNA419519	SAMN14314817	SRR11262138	JAFFBK000000000
**MF6243**	2014	M1	meat	food processing environment	ST9	CC9	II	MF1576	PRJNA419519	SAMN14314818	SRR11262137	JAFFBJ000000000
**MF6244**	2014	M1	meat	food processing environment	ST9	CC9	II	MF1576	PRJNA419519	SAMN14314819	SRR11262136	JAFFBI000000000
**MF6245**	2014	M1	meat	food processing environment	ST9	CC9	II	MF1576	PRJNA419519	SAMN14314820	SRR11262135	JAFFBH000000000
**MF6246**	2014	M1	meat	food processing environment	ST9	CC9	II	MF1576	PRJNA419519	SAMN14314821	SRR11262134	JAFFBG000000000
**MF6247**	2014	M1	meat	food processing environment	ST9	CC9	II	MF1576	PRJNA419519	SAMN14314822	SRR11262133	JAFFBF000000000
**MF6248**	2014	M1	meat	food processing environment	ST9	CC9	II	MF1576	PRJNA419519	SAMN14314823	SRR11262132	JAFFBE000000000
**MF6249**	2014	M2	meat	food processing environment	ST7	CC7	II	MF2133	PRJNA689484	SAMN17224357	SRR13588147	JAERCW000000000
**MF6250**	2014	M2	meat	food processing environment	ST7	CC7	II	MF2133	PRJNA689484	SAMN17224358	SRR13588146	JAERCV000000000
**MF6251**	2014	M2	meat	food processing environment	ST7	CC7	II	MF2133	PRJNA689484	SAMN17224359	SRR13588145	JAERCU000000000
**MF6252**	2014	M2	meat	food processing environment	ST7	CC7	II	MF2133	PRJNA689484	SAMN17224360	SRR13588144	JAERCT000000000
**MF6253**	2014	M2	meat	food processing environment	ST7	CC7	II	MF2133	PRJNA689484	SAMN17224361	SRR13588143	JAERCS000000000

**MF6254**	2014	M4	meat	food product (cooked)	ST9	CC9	II	MF1576	PRJNA419519	SAMN14314824	SRR11262130	JAFFBD000000000
**MF6255**	2014	M4	meat	food product (cooked)	ST9	CC9	II	MF1576	PRJNA419519	SAMN14314825	SRR11262129	JAFFBC000000000
**MF6256**	2014	M4	meat	food product (cooked)	ST9	CC9	II	MF1576	PRJNA419519	SAMN14314826	SRR11262128	JAFFBB000000000
**MF6257**	2014	M4	meat	food product (cooked)	ST9	CC9	II	MF1576	PRJNA419519	SAMN14314827	SRR11262127	JAFFBA000000000
**MF6258**	2014	M4	meat	food product (cooked)	ST9	CC9	II	MF1576	PRJNA419519	SAMN14314828	SRR11262126	JAFFAZ000000000
**MF6259**	2014	M4	meat	food product (cooked)	ST9	CC9	II	MF1576	PRJNA419519	SAMN14314829	SRR11262125	JAFFAY000000000
**MF6260**	2014	M4	meat	food product (cooked)	ST9	CC9	II	MF1576	PRJNA419519	SAMN14314830	SRR11262124	JAFFAX000000000
**MF6261**	2014	M4	meat	food processing environment	ST9	CC9	II	MF1576	PRJNA419519	SAMN14314831	SRR11262123	JAFFAW000000000
**MF6262**	2014	M4	meat	food processing environment	ST9	CC9	II	MF1576	PRJNA419519	SAMN14314832	SRR11262122	JAFFAV000000000
**MF6263**	2014	M4	meat	food processing environment	ST9	CC9	II	MF1576	PRJNA419519	SAMN14314833	SRR11261999	JAFFAU000000000
**MF6265**	2014	M4	meat	food processing environment	ST9	CC9	II	MF1576	PRJNA419519	SAMN14314834	SRR11261997	JAFFAT000000000
**MF6267**	2014	M4	meat	food processing environment	ST9	CC9	II	MF1576	PRJNA419519	SAMN14314835	SRR11261996	JAFFAS000000000
**MF6268**	2014	M4	meat	food processing environment	ST9	CC9	II	MF1576	PRJNA419519	SAMN14314836	SRR11261995	JAFFAR000000000
**MF6270**	2014	M4	meat	food processing environment	ST9	CC9	II	MF1576	PRJNA419519	SAMN14314837	SRR11261994	JAFFAQ000000000
**MF6272**	2014	M4	meat	food processing environment	ST9	CC9	II	MF1576	PRJNA419519	SAMN14314838	SRR11261993	JAFFAP000000000
**MF6273**	2014	M4	meat	food processing environment	ST9	CC9	II	MF1576	PRJNA419519	SAMN14314839	SRR11261992	JAFFAO000000000
**MF6275**	2014	M4	meat	food processing environment	ST9	CC9	II	MF1576	PRJNA419519	SAMN14314840	SRR11261991	JAFFAN000000000
**MF6276**	2014	M4	meat	food processing environment	ST9	CC9	II	MF1576	PRJNA419519	SAMN14314841	SRR11261990	JAFFAM000000000
**MF6278**	2014	M4	meat	food processing environment	ST9	CC9	II	MF1576	PRJNA419519	SAMN14314842	SRR11261989	JAFFAL000000000
**MF6279**	2014	M4	meat	food processing environment	ST9	CC9	II	MF1576	PRJNA419519	SAMN14314843	SRR11261988	JAFFAK000000000
**MF6281**	2014	M4	meat	food processing environment	ST9	CC9	II	MF1576	PRJNA419519	SAMN14314844	SRR11261986	JAFFAJ000000000
**MF6283**	2014	M4	meat	food processing environment	ST9	CC9	II	MF1576	PRJNA419519	SAMN14314845	SRR11261985	JAFFAI000000000
**MF6284**	2014	M4	meat	food processing environment	ST9	CC9	II	MF1576	PRJNA419519	SAMN14314846	SRR11261984	JAFFAH000000000
**MF6285**	2014	M4	meat	food processing environment	ST9	CC9	II	MF1576	PRJNA419519	SAMN14314847	SRR11261983	JAFFAG000000000
**MF6286**	2014	M4	meat	food product (cooked)	ST9	CC9	II	MF1576	PRJNA419519	SAMN14314848	SRR11261982	JAFFAF000000000
**MF6287**	2014	M4	meat	food product (cooked)	ST9	CC9	II	MF1576	PRJNA419519	SAMN14314849	SRR11261981	JAFFAE000000000
**MF6289**	2014	M4	meat	food product (cooked)	ST9	CC9	II	MF1576	PRJNA419519	SAMN14314850	SRR11261980	JAFFAD000000000
**MF6290**	2014	M4	meat	food processing environment	ST9	CC9	II	MF1576	PRJNA419519	SAMN14314851	SRR11261979	JAFFAC000000000
**MF6291**	2014	M4	meat	food processing environment	ST9	CC9	II	MF1576	PRJNA419519	SAMN14314852	SRR11261978	JAFFAB000000000
**MF6292**	2014	M4	meat	food processing environment	ST9	CC9	II	MF1576	PRJNA419519	SAMN14314853	SRR11261977	JAFFAA000000000
**MF6293**	2014	M4	meat	food product (raw)	ST7	CC7	II	MF2133	PRJNA689484	SAMN17224363	SRR13588141	JAERCR000000000
**MF6294**	2014	M4	meat	food processing environment	ST9	CC9	II	MF1576	PRJNA419519	SAMN14314854	SRR11261975	JAFEZZ000000000
**MF6295**	2014	M4	meat	food processing environment	ST9	CC9	II	MF1576	PRJNA419519	SAMN14314855	SRR11261974	JAFEZY000000000
**MF6296**	2014	M4	meat	food processing environment	ST9	CC9	II	MF1576	PRJNA419519	SAMN14314856	SRR11261973	JAFEZX000000000
**MF6297**	2014	M4	meat	food product (cooked)	ST9	CC9	II	MF1576	PRJNA419519	SAMN14314857	SRR11261972	JAFEZW000000000
**MF6298**	2014	M4	meat	food product (cooked)	ST9	CC9	II	MF1576	PRJNA419519	SAMN14314858	SRR11261971	JAFEZV000000000
**MF6299**	2014	M4	meat	food processing environment	ST9	CC9	II	MF1576	PRJNA419519	SAMN14314859	SRR11261970	JAFEZU000000000
**MF6300**	2014	M4	meat	food processing environment	ST9	CC9	II	MF1576	PRJNA419519	SAMN14314860	SRR11261969	JAFEZT000000000
**MF6301**	2014	M4	meat	food processing environment	ST9	CC9	II	MF1576	PRJNA419519	SAMN14314861	SRR11261968	JAFEZS000000000
**MF6302**	2014	M4	meat	food processing environment	ST9	CC9	II	MF1576	PRJNA419519	SAMN14314862	SRR11262121	JAFEZR000000000
**MF6303**	2014	M4	meat	food processing environment	ST9	CC9	II	MF1576	PRJNA419519	SAMN14314863	SRR11262120	JAFEZQ000000000
**MF6304**	2014	M4	meat	food processing environment	ST9	CC9	II	MF1576	PRJNA419519	SAMN14314864	SRR11262118	JAFEZP000000000
**MF6306**	2014	M4	meat	food processing environment	ST9	CC9	II	MF1576	PRJNA419519	SAMN14314865	SRR11262117	JAFEZO000000000
**MF6307**	2014	M4	meat	food processing environment	ST9	CC9	II	MF1576	PRJNA419519	SAMN14314866	SRR11262116	JAFEZN000000000
**MF6308**	2014	M4	meat	food processing environment	ST9	CC9	II	MF1576	PRJNA419519	SAMN14314867	SRR11262115	JAFEZM000000000
**MF6309**	2014	M4	meat	food processing environment	ST9	CC9	II	MF1576	PRJNA419519	SAMN14314868	SRR11262114	JAFEZL000000000

**MF6310**	2014	M4	meat	food processing environment	ST9	CC9	II	MF1576	PRJNA419519	SAMN14314869	SRR11262113	JAFEZK000000000
**MF6311**	2014	M4	meat	food processing environment	ST9	CC9	II	MF1576	PRJNA419519	SAMN14314870	SRR11262112	JAFEZJ000000000
**MF6312**	2014	M4	meat	food processing environment	ST9	CC9	II	MF1576	PRJNA419519	SAMN14314871	SRR11262111	JAFEZI000000000
**MF6313**	2014	M4	meat	food processing environment	ST9	CC9	II	MF1576	PRJNA419519	SAMN14314872	SRR11262110	JAFEZH000000000
**MF6316**	2014	M4	meat	food processing environment	ST9	CC9	II	MF1576	PRJNA419519	SAMN14314873	SRR11262109	JAFEZG000000000
**MF6317**	2014	M4	meat	food processing environment	ST9	CC9	II	MF1576	PRJNA419519	SAMN14314874	SRR11262106	JAFEZF000000000
**MF6318**	2014	M4	meat	food processing environment	ST9	CC9	II	MF1576	PRJNA419519	SAMN14314875	SRR11262105	JAFEZE000000000
**MF6320**	2014	M4	meat	food processing environment	ST9	CC9	II	MF1576	PRJNA419519	SAMN14314876	SRR11262104	JAFEZD000000000
**MF6324**	2014	M4	meat	food processing environment	ST9	CC9	II	MF1576	PRJNA419519	SAMN14314877	SRR11262103	JAFEZC000000000
**MF6329**	2015	M4	meat	food processing environment	ST9	CC9	II	MF1576	PRJNA419519	SAMN14314878	SRR11262102	JAFEZB000000000
**MF6330**	2015	M4	meat	food processing environment	ST9	CC9	II	MF1576	PRJNA419519	SAMN14314879	SRR11262101	JAFEZA000000000
**MF6331**	2015	M4	meat	food processing environment	ST121	CC121	II	MF4804	PRJNA689484	SAMN17224366	SRR13378850	JAERCQ000000000
**MF6332**	2015	M4	meat	food processing environment	ST9	CC9	II	MF1576	PRJNA419519	SAMN14314880	SRR11262100	JAFEYZ000000000
**MF6333**	2015	M4	meat	food processing environment	ST6	CC6	I	MF7750	PRJNA689484	SAMN17224367	SRR13378839	JAERCP000000000
**MF6334**	2015	M4	meat	food processing environment	ST9	CC9	II	MF1576	PRJNA419519	SAMN14314881	SRR11262099	JAFEYY000000000
**MF6335**	2015	M4	meat	food processing environment	ST121	CC121	II	MF4804	PRJNA689484	SAMN17224368	SRR13378828	JAERCO000000000
**MF6336**	2015	M4	meat	food processing environment	ST394	CC415	II	MF7713	PRJNA689484	SAMN17224369	SRR13378817	JAERCN000000000
**MF6337**	2015	M4	meat	food processing environment	ST9	CC9	II	MF1576	PRJNA419519	SAMN14314882	SRR11262098	JAFEYX000000000
**MF6338**	2015	M7	meat	food processing - low hygienic zone	ST9	CC9	II	MF1576	PRJNA419519	SAMN14314883	SRR11262097	JAFEYW000000000
**MF6339**	2015	M7	meat	food processing - low hygienic zone	ST9	CC9	II	MF1576	PRJNA419519	SAMN14314884	SRR11262095	JAFEYV000000000
**MF6340**	2015	M7	meat	food processing - low hygienic zone	ST121	CC121	II	MF4804	PRJNA689484	SAMN17224370	SRR13378806	JAERCM000000000
**MF6341**	2015	M7	meat	food processing - low hygienic zone	ST18	CC18	II	MF4566	PRJNA689484	SAMN17224371	SRR13378929	JAERCL000000000
**MF6342**	2015	M7	meat	food processing - low hygienic zone	ST121	CC121	II	MF4804	PRJNA689484	SAMN17224372	SRR13378918	JAERCK000000000
**MF6343**	2015	M7	meat	food processing - low hygienic zone	ST9	CC9	II	MF1576	PRJNA419519	SAMN14314885	SRR11262094	JAFEYU000000000
**MF6344**	2015	M4	meat	food processing environment	ST121	CC121	II	MF4804	PRJNA689484	SAMN17224373	SRR13378907	JAERCJ000000000
**MF6345**	2015	M4	meat	food processing environment	ST9	CC9	II	MF1576	PRJNA419519	SAMN14314886	SRR11262093	JAFEYT000000000
**MF6346**	2015	M4	meat	food processing environment	ST9	CC9	II	MF1576	PRJNA419519	SAMN14314887	SRR11262092	JAFEYS000000000
**MF6348**	2015	M4	meat	food processing environment	ST9	CC9	II	MF1576	PRJNA419519	SAMN14314888	SRR11262091	JAFEYR000000000
**MF6349**	2015	M4	meat	food processing environment	ST4	CC4	I		PRJNA689484	SAMN17224374	SRR13378896	JAERCI000000000
**MF6350**	2015	M4	meat	food processing environment	ST9	CC9	II	MF1576	PRJNA419519	SAMN14314889	SRR11262090	JAFEYQ000000000
**MF6352**	2015	M4	meat	food processing environment	ST121	CC121	II	MF4804	PRJNA689484	SAMN17224375	SRR13378889	JAERCH000000000
**MF6353**	2015	M4	meat	food processing environment	ST394	CC415	II	MF7713	PRJNA689484	SAMN17224376	SRR13378888	JAERCG000000000
**MF6355**	2015	M4	meat	food processing environment	ST121	CC121	II	MF4804	PRJNA689484	SAMN17224377	SRR13378887	JAERCF000000000
**MF6356**	2015	M4	meat	food processing environment	ST9	CC9	II	MF1576	PRJNA419519	SAMN14314890	SRR11262089	JAFEYP000000000
**MF7690**	2015	M1	meat	food processing environment	ST9	CC9	II	MF1576	PRJNA689484	SAMN17224559	SRR13588444	JAEQVF000000000
**MF7691**	2015	M1	meat	food processing environment	ST9	CC9	II	MF1576	PRJNA689484	SAMN17224560	SRR13588443	JAEQVE000000000
**MF7692**	2015	M1	meat	food processing environment	ST9	CC9	II	MF1576	PRJNA689484	SAMN17224561	SRR13588442	JAEQVD000000000
**MF7693**	2015	M1	meat	food processing environment	ST9	CC9	II	MF1576	PRJNA689484	SAMN17224562	SRR13588441	JAEQVC000000000
**MF7680**	2016	M6	meat	food processing environment	ST5	CC5	I	MF7680	PRJNA689484	SAMN17224549	SRR13588333	JAEQVP000000000
**MF7681**	2016	M6	meat	food processing environment	ST5	CC5	I	MF7680	PRJNA689484	SAMN17224550	SRR13588332	JAEQVO000000000
**MF7682**	2016	M6	meat	food processing environment	ST5	CC5	I	MF7680	PRJNA689484	SAMN17224551	SRR13588331	JAEQVN000000000
**MF7683**	2016	M6	meat	food processing environment	ST5	CC5	I	MF7680	PRJNA689484	SAMN17224552	SRR13588330	JAEQVM000000000
**MF6577**	2017	M1	meat	food processing environment	ST9	CC9	II	MF1576	PRJNA419519	SAMN14314891	SRR11262088	JAFEYO000000000
**MF6578**	2017	M1	meat	food processing environment	ST9	CC9	II	MF1576	PRJNA419519	SAMN14314892	SRR11262087	JAFEYN000000000
**MF6579**	2017	M1	meat	food processing environment	ST9	CC9	II	MF1576	PRJNA419519	SAMN14314893	SRR11262086	JAFEYM000000000
**MF6582**	2017	M1	meat	food processing environment	ST9	CC9	II	MF1576	PRJNA419519	SAMN14314894	SRR11262084	JAFEYL000000000

**MF6587**	2017	M1	meat	food processing environment	ST9	CC9	II	MF1576	PRJNA419519	SAMN14314895	SRR11262083	JAFEYK000000000
**MF6588**	2017	M1	meat	food processing environment	ST9	CC9	II	MF1576	PRJNA419519	SAMN14314896	SRR11262082	JAFEYJ000000000
**MF6589**	2017	M1	meat	food processing environment	ST9	CC9	II	MF1576	PRJNA419519	SAMN14314897	SRR11262081	JAFEYI000000000
**MF6590**	2017	M1	meat	food processing environment	ST9	CC9	II	MF1576	PRJNA419519	SAMN14314898	SRR11262080	JAFEYH000000000
**MF6591**	2017	M4	meat	food processing environment	ST9	CC9	II	MF1576	PRJNA419519	SAMN14314899	SRR11262079	JAFEYG000000000
**MF6592**	2017	M1	meat	food processing environment	ST9	CC9	II	MF1576	PRJNA419519	SAMN14314900	SRR11262078	JAFEYF000000000
**MF6593**	2017	M1	meat	food processing environment	ST9	CC9	II	MF1576	PRJNA419519	SAMN14314901	SRR11262077	JAFEYE000000000
**MF6705**	2017	M1	meat	food processing environment	ST9	CC9	II	MF1576	PRJNA419519	SAMN14314902	SRR11262076	JAFEYD000000000
**MF6706**	2017	M1	meat	food processing environment	ST9	CC9	II	MF1576	PRJNA419519	SAMN14314903	SRR11262075	JAFEYC000000000
**MF6707**	2017	M1	meat	food processing environment	ST9	CC9	II	MF1576	PRJNA419519	SAMN14314904	SRR11262073	JAFEYB000000000
**MF6708**	2017	M4	meat	food processing environment	ST101	CC101	II		PRJNA689484	SAMN17224378	SRR13588270	JAERCE000000000
**MF6709**	2017	M1	meat	food processing environment	ST9	CC9	II	MF1576	PRJNA419519	SAMN14314905	SRR11262072	JAFEYA000000000
**MF6710**	2017	M1	meat	food processing environment	ST9	CC9	II	MF1576	PRJNA419519	SAMN14314906	SRR11262071	JAFEXZ000000000
**MF6796**	2017	M4	meat	food processing environment	ST121	CC121	II	MF4804	PRJNA689484	SAMN17224379	SRR13588269	JAERCD000000000
**MF6797**	2017	M1	meat	food processing environment	ST9	CC9	II	MF1576	PRJNA419519	SAMN14314907	SRR11262070	JAFEXY000000000
**MF6798**	2017	M4	meat	food processing environment	ST394	CC415	II	MF7713	PRJNA689484	SAMN17224380	SRR13588268	JAERCC000000000
**MF6799**	2017	M1	meat	food processing environment	ST9	CC9	II	MF1576	PRJNA419519	SAMN14314908	SRR11262069	JAFEXX000000000
**MF6800**	2017	M1	meat	food processing environment	ST9	CC9	II	MF1576	PRJNA419519	SAMN14314909	SRR11262068	JAFEXW000000000
**MF6801**	2017	M1	meat	food processing environment	ST9	CC9	II	MF1576	PRJNA419519	SAMN14314910	SRR11262067	JAFEXV000000000
**MF6802**	2017	M1	meat	food processing environment	ST9	CC9	II	MF1576	PRJNA419519	SAMN14314911	SRR11262066	JAFEXU000000000
**MF6803**	2017	M1	meat	food processing environment	ST9	CC9	II	MF1576	PRJNA419519	SAMN14314912	SRR11262065	JAFEXT000000000
**MF6809**	2017	M1	meat	food processing environment	ST9	CC9	II	MF1576	PRJNA419519	SAMN14314913	SRR11262064	JAFEXS000000000
**MF6810**	2017	M1	meat	food processing environment	ST9	CC9	II	MF1576	PRJNA419519	SAMN14314914	SRR11262062	JAFEXR000000000
**MF6811**	2017	M1	meat	food processing environment	ST9	CC9	II	MF1576	PRJNA419519	SAMN14314915	SRR11262061	JAFEXQ000000000
**MF6812**	2017	M4	meat	food processing environment	ST1416	CC19	II	MF7335	PRJNA689484	SAMN17224381	SRR13588267	JAERCB000000000
**MF6813**	2017	M4	meat	food processing environment	ST1416	CC19	II	MF7335	PRJNA689484	SAMN17224382	SRR13588266	JAERCA000000000
**MF6814**	2017	M4	meat	food processing environment	ST1416	CC19	II	MF7335	PRJNA689484	SAMN17224383	SRR13588264	JAERBZ000000000
**MF6815**	2017	M4	meat	food processing environment	ST1416	CC19	II	MF7335	PRJNA689484	SAMN17224384	SRR13588263	JAERBY000000000
**MF6817**	2017	M1	meat	food processing environment	ST9	CC9	II	MF1576	PRJNA419519	SAMN14314916	SRR11262060	JAFEXP000000000
**MF6818**	2017	M1	meat	food processing environment	ST9	CC9	II	MF1576	PRJNA419519	SAMN14314917	SRR11262059	JAFEXO000000000
**MF6819**	2017	M1	meat	food processing environment	ST9	CC9	II	MF1576	PRJNA419519	SAMN14314918	SRR11262058	JAFEXN000000000
**MF6820**	2017	M1	meat	food processing environment	ST9	CC9	II	MF1576	PRJNA419519	SAMN14314919	SRR11262185	JAFEXM000000000
**MF6821**	2017	M1	meat	food processing environment	ST9	CC9	II	MF1576	PRJNA419519	SAMN14314920	SRR11262184	JAFEXL000000000
**MF6822**	2017	M1	meat	food processing environment	ST9	CC9	II	MF1576	PRJNA419519	SAMN14314921	SRR11262183	JAFEXK000000000
**MF6823**	2017	M1	meat	food processing environment	ST9	CC9	II	MF1576	PRJNA419519	SAMN14314922	SRR11262182	JAFEXJ000000000
**MF6824**	2017	M1	meat	food processing environment	ST9	CC9	II	MF1576	PRJNA419519	SAMN14314923	SRR11262181	JAFEXI000000000
**MF6836**	2017	M1	meat	food processing environment	ST9	CC9	II	MF1576	PRJNA419519	SAMN14314924	SRR11262179	JAFEXH000000000
**MF6837**	2017	M1	meat	food processing environment	ST9	CC9	II	MF1576	PRJNA419519	SAMN14314925	SRR11262178	JAFEXG000000000
**MF7318**	2017	M8	meat	food processing environment	ST9	CC9	II	MF1576	PRJNA689484	SAMN17224462	SRR13588320	JAEQYY000000000
**MF7320**	2017	M8	meat	food processing environment	ST9	CC9	II	MF1576	PRJNA689484	SAMN17224463	SRR13588323	JAEQYX000000000
**MF7321**	2017	M8	meat	food processing environment	ST9	CC9	II	MF1576	PRJNA689484	SAMN17224464	SRR13588319	JAEQYW000000000
**MF7324**	2017	M8	meat	food processing environment	ST9	CC9	II	MF1576	PRJNA689484	SAMN17224465	SRR13588318	JAEQYV000000000
**MF7325**	2017	M8	meat	food processing environment	ST121	CC121	II	MF4804	PRJNA689484	SAMN17224466	SRR13588317	JAEQYU000000000
**MF7326**	2017	M8	meat	food processing environment	ST9	CC9	II	MF1576	PRJNA689484	SAMN17224467	SRR13588316	JAEQYT000000000
**MF7684**	2017	M6	meat	food processing environment	ST5	CC5	I	MF7680	PRJNA689484	SAMN17224553	SRR13588329	JAEQVL000000000
**MF7685**	2017	M6	meat	food processing environment	ST5	CC5	I	MF7680	PRJNA689484	SAMN17224554	SRR13588328	JAEQVK000000000

**MF7686**	2017	M6	meat	food processing environment	ST5	CC5	I	MF7680	PRJNA689484	SAMN17224555	SRR13588327	JAEQVJ000000000
**MF7403**	2018	M8	meat	food processing environment	ST9	CC9	II	MF1576	PRJNA689484	SAMN17224507	SRR13588294	JAEQXF000000000
**MF7408**	2018	M8	meat	food processing environment	ST199	CC199	II	MF7408	PRJNA689484	SAMN17224508	SRR13588293	JAEQXE000000000
**MF7687**	2018	M6	meat	food processing environment	ST5	CC5	I	MF7680	PRJNA689484	SAMN17224556	SRR13588447	JAEQVI000000000
**MF7688**	2018	M6	meat	food processing environment	ST5	CC5	I	MF7680	PRJNA689484	SAMN17224557	SRR13588446	JAEQVH000000000
**MF7263**	2019	M7	meat	food processing environment	ST7	CC7	II	MF2133	PRJNA689484	SAMN17224447	SRR13588140	JAEQZN000000000
**MF7413**	2019	M8	meat	food processing environment	ST121	CC121	II	MF4804	PRJNA689484	SAMN17224509	SRR13588292	JAEQXD000000000
**MF7416**	2019	M8	meat	food processing environment	ST9	CC9	II	MF1576	PRJNA689484	SAMN17224510	SRR13588291	JAEQXC000000000
**MF7420**	2019	M8	meat	food processing environment	ST121	CC121	II	MF4804	PRJNA689484	SAMN17224511	SRR13588290	JAEQXB000000000
**MF7421**	2019	M8	meat	food processing environment	ST9	CC9	II	MF1576	PRJNA689484	SAMN17224512	SRR13588289	JAEQXA000000000
**MF7426**	2019	M8	meat	food processing environment	ST9	CC9	II	MF1576	PRJNA689484	SAMN17224513	SRR13588287	JAEQWZ000000000
**MF7427**	2019	M8	meat	food processing environment	ST9	CC9	II	MF1576	PRJNA689484	SAMN17224514	SRR13588286	JAEQWY000000000
**MF7429**	2019	M8	meat	food processing environment	ST9	CC9	II	MF1576	PRJNA689484	SAMN17224515	SRR13588285	JAEQWX000000000
**MF7430**	2019	M8	meat	food processing environment	ST9	CC9	II	MF1576	PRJNA689484	SAMN17224516	SRR13588284	JAEQWW000000000
**MF7432**	2019	M8	meat	food processing environment	ST9	CC9	II	MF1576	PRJNA689484	SAMN17224517	SRR13588283	JAEQWV000000000
**MF7437**	2019	M8	meat	food processing environment	ST9	CC9	II	MF1576	PRJNA689484	SAMN17224518	SRR13378886	JAEQWU000000000
**MF7456**	2019	M7	meat	food processing environment	ST8	CC8	II	MF4245	PRJNA689484	SAMN17224519	SRR13588139	JAEQWT000000000
**MF7586**	2019	M7	meat	food processing environment	ST7	CC7	II	MF2133	PRJNA689484	SAMN17224520	SRR13588138	JAEQWS000000000
**MF7587**	2019	M7	meat	food processing environment	ST7	CC7	II	MF2133	PRJNA689484	SAMN17224521	SRR13588137	JAEQWR000000000
**MF7648**	2019	M8	meat	food product (cooked)	ST451	CC11	II	MF4627	PRJNA689484	SAMN17224522	SRR13588136	JAEQWQ000000000
**MF7649**	2019	M8	meat	food processing environment	ST199	CC199	II	MF7408	PRJNA689484	SAMN17224523	SRR13588135	JAEQWP000000000
**MF7650**	2019	M8	meat	food processing environment	ST199	CC199	II	MF7408	PRJNA689484	SAMN17224524	SRR13588134	JAEQWO000000000
**MF7651**	2019	M8	meat	food processing environment	ST121	CC121	II	MF4804	PRJNA689484	SAMN17224525	SRR13588133	JAEQWN000000000
**MF7654**	2019	M8	meat	food processing environment	ST121	CC121	II	MF4804	PRJNA689484	SAMN17224526	SRR13588132	JAEQWM000000000
**MF7657**	2019	M8	meat	food processing environment	ST121	CC121	II	MF4804	PRJNA689484	SAMN17224527	SRR13588334	JAEQWL000000000
**MF7659**	2019	M1	meat	food processing environment	ST91	CC91	II	MF7663	PRJNA689484	SAMN17224528	SRR13588130	JAEQWK000000000
**MF7660**	2019	M1	meat	food processing environment	ST91	CC91	II	MF7663	PRJNA689484	SAMN17224529	SRR13588129	JAEQWJ000000000
**MF7661**	2019	M1	meat	food processing environment	ST8	CC8	II	MF4245	PRJNA689484	SAMN17224530	SRR13588128	JAEQWI000000000
**MF7662**	2019	M1	meat	food processing environment	ST121	CC121	II	MF4804	PRJNA689484	SAMN17224531	SRR13588127	JAEQWH000000000
**MF7663**	2019	M1	meat	food processing environment	ST91	CC91	II	MF7663	PRJNA689484	SAMN17224532	SRR13588126	JAEQWG000000000
**MF7664**	2019	M1	meat	food processing environment	ST91	CC91	II	MF7663	PRJNA689484	SAMN17224533	SRR13588125	JAEQWF000000000
**MF7665**	2019	M1	meat	food processing environment	ST8	CC8	II	MF4245	PRJNA689484	SAMN17224534	SRR13588124	JAEQWE000000000
**MF7666**	2019	M1	meat	food processing environment	ST8	CC8	II	MF4245	PRJNA689484	SAMN17224535	SRR13588123	JAEQWD000000000
**MF7667**	2019	M1	meat	food processing environment	ST394	CC415	II	MF7713	PRJNA689484	SAMN17224536	SRR13588122	JAEQWC000000000
**MF7668**	2019	M1	meat	food processing - low hygienic zone	ST14	CC14	II	MF3939	PRJNA689484	SAMN17224537	SRR13588121	JAEQWB000000000
**MF7669**	2019	M1	meat	food processing - low hygienic zone	ST91	CC91	II	MF7663	PRJNA689484	SAMN17224538	SRR13588119	JAEQWA000000000
**MF7670**	2019	M1	meat	food processing - low hygienic zone	ST220	CC220	I	MF7752	PRJNA689484	SAMN17224539	SRR13588118	JAEQVZ000000000
**MF7671**	2019	M1	meat	food processing - low hygienic zone	ST451	CC11	II	MF4627	PRJNA689484	SAMN17224540	SRR13588117	JAEQVY000000000
**MF7672**	2019	M1	meat	food processing environment	ST9	CC9	II	MF1576	PRJNA689484	SAMN17224541	SRR13588116	JAEQVX000000000
**MF7673**	2019	M1	meat	food processing environment	ST9	CC9	II	MF1576	PRJNA689484	SAMN17224542	SRR13588115	JAEQVW000000000
**MF7674**	2019	M1	meat	food processing environment	ST451	CC11	II	MF4627	PRJNA689484	SAMN17224543	SRR13588114	JAEQVV000000000
**MF7675**	2019	M1	meat	food processing environment	ST451	CC11	II	MF4627	PRJNA689484	SAMN17224544	SRR13588113	JAEQVU000000000
**MF7676**	2019	M1	meat	food processing environment	ST1416	CC19	II	MF7335	PRJNA689484	SAMN17224545	SRR13588112	JAEQVT000000000
**MF7677**	2019	M1	meat	food processing environment	ST2344	CC9	II	MF1576	PRJNA689484	SAMN17224546	SRR13588111	JAEQVS000000000
**MF7678**	2019	M1	meat	food processing - low hygienic zone	ST37	CC37	II	MF7858	PRJNA689484	SAMN17224547	SRR13588110	JAEQVR000000000
**MF7679**	2019	M1	meat	food processing environment	ST8	CC8	II	MF4245	PRJNA689484	SAMN17224548	SRR13588108	JAEQVQ000000000

**MF7689**	2019	M6	meat	food processing environment	ST5	CC5	I	MF7680	PRJNA689484	SAMN17224558	SRR13588445	JAEQVG000000000
**MF7744**	2019	M9	meat	food processing environment	ST21	CC21	II	MF7744	PRJNA689484	SAMN17224613	SRR13588438	JAEQTD000000000
**MF7745**	2019	M9	meat	food processing environment	ST9	CC9	II	MF1576	PRJNA689484	SAMN17224614	SRR13588436	JAEQTC000000000
**MF7746**	2019	M9	meat	food processing environment	ST9	CC9	II	MF1576	PRJNA689484	SAMN17224615	SRR13588435	JAEQTB000000000
**MF7747**	2019	M9	meat	food processing environment	ST9	CC9	II	MF1576	PRJNA689484	SAMN17224616	SRR13588434	JAEQTA000000000
**MF7748**	2019	M9	meat	food processing environment	ST6	CC6	I	MF7750	PRJNA689484	SAMN17224617	SRR13588433	JAEQSZ000000000
**MF7749**	2019	M9	meat	food processing environment	ST9	CC9	II	MF1576	PRJNA689484	SAMN17224618	SRR13588432	JAEQSY000000000
**MF7750**	2019	M9	meat	food processing environment	ST6	CC6	I	MF7750	PRJNA689484	SAMN17224619	SRR13588431	JAEQSX000000000
**MF7751**	2019	M9	meat	food processing environment	ST91	CC91	II	MF7663	PRJNA689484	SAMN17224620	SRR13588430	JAEQSW000000000
**MF7752**	2019	M9	meat	food processing - low hygienic zone	ST220	CC220	I	MF7752	PRJNA689484	SAMN17224621	SRR13588429	JAEQSV000000000
**MF7753**	2019	M9	meat	food processing - low hygienic zone	ST451	CC11	II	MF4627	PRJNA689484	SAMN17224622	SRR13588428	JAEQSU000000000
**MF7754**	2019	M9	meat	food processing - low hygienic zone	ST394	CC415	II	MF7713	PRJNA689484	SAMN17224623	SRR13588427	JAEQST000000000
**MF7807**	2019	M4	meat	food processing environment	ST394	CC415	II	MF7713	PRJNA689484	SAMN17224625	SRR13588422	JAEQSR000000000
**MF7808**	2019	M4	meat	food processing environment	ST1416	CC19	II	MF7335	PRJNA689484	SAMN17224626	SRR13588421	JAEQSQ000000000
**MF7809**	2019	M4	meat	food processing environment	ST1416	CC19	II	MF7335	PRJNA689484	SAMN17224627	SRR13588420	JAEQSP000000000
**MF7810**	2019	M4	meat	food processing environment	ST1416	CC19	II	MF7335	PRJNA689484	SAMN17224628	SRR13588419	JAEQSO000000000
**MF7811**	2019	M4	meat	food processing environment	ST394	CC415	II	MF7713	PRJNA689484	SAMN17224629	SRR13588418	JAEQSN000000000
**MF7812**	2019	M4	meat	food processing environment	ST9	CC9	II	MF1576	PRJNA689484	SAMN17224630	SRR13588417	JAEQSM000000000
**MF7813**	2019	M4	meat	food processing environment	ST1416	CC19	II	MF7335	PRJNA689484	SAMN17224631	SRR13588416	JAEQSL000000000
**MF7814**	2019	M4	meat	food processing environment	ST1416	CC19	II	MF7335	PRJNA689484	SAMN17224632	SRR13588415	JAEQSK000000000
**MF7815**	2019	M4	meat	food processing environment	ST9	CC9	II	MF1576	PRJNA689484	SAMN17224633	SRR13588414	JAEQSJ000000000
**MF7816**	2019	M4	meat	food processing environment	ST1416	CC19	II	MF7335	PRJNA689484	SAMN17224634	SRR13378885	JAEQSI000000000
**MF7817**	2019	M4	meat	food processing environment	ST1416	CC19	II	MF7335	PRJNA689484	SAMN17224635	SRR13378884	JAEQSH000000000
**MF7818**	2019	M4	meat	food processing environment	ST1416	CC19	II	MF7335	PRJNA689484	SAMN17224636	SRR13378882	JAEQSG000000000
**MF7819**	2019	M4	meat	food processing environment	ST1416	CC19	II	MF7335	PRJNA689484	SAMN17224637	SRR13378881	JAEQSF000000000
**MF7820**	2019	M4	meat	food processing environment	ST1416	CC19	II	MF7335	PRJNA689484	SAMN17224638	SRR13378880	JAEQSE000000000
**MF7821**	2019	M4	meat	food processing environment	ST1416	CC19	II	MF7335	PRJNA689484	SAMN17224639	SRR13378879	JAEQSD000000000
**MF7822**	2019	M4	meat	food processing environment	ST9	CC9	II	MF1576	PRJNA689484	SAMN17224640	SRR13378878	JAEQSC000000000
**MF7823**	2019	M4	meat	food processing environment	ST1416	CC19	II	MF7335	PRJNA689484	SAMN17224641	SRR13378877	JAEQSB000000000
**MF7824**	2019	M4	meat	food processing environment	ST121	CC121	II	MF4804	PRJNA689484	SAMN17224642	SRR13588412	JAEQSA000000000
**MF7825**	2019	M4	meat	food processing environment	ST1416	CC19	II	MF7335	PRJNA689484	SAMN17224643	SRR13588411	JAEQRZ000000000
**MF7826**	2020	M4	meat	food processing environment	ST1416	CC19	II	MF7335	PRJNA689484	SAMN17224644	SRR13588410	JAEQRY000000000
**MF7827**	2020	M4	meat	food processing environment	ST394	CC415	II	MF7713	PRJNA689484	SAMN17224645	SRR13378876	JAEQRX000000000
**MF7828**	2020	M4	meat	food processing environment	ST1416	CC19	II	MF7335	PRJNA689484	SAMN17224646	SRR13378875	JAEQRW000000000
**MF7829**	2020	M4	meat	food processing environment	ST1416	CC19	II	MF7335	PRJNA689484	SAMN17224647	SRR13378874	JAEQRV000000000
**MF7830**	2020	M4	meat	food processing environment	ST1416	CC19	II	MF7335	PRJNA689484	SAMN17224648	SRR13378873	JAEQRU000000000
**MF7835**	2020	M4	meat	food processing environment	ST394	CC415	II	MF7713	PRJNA689484	SAMN17224649	SRR13378871	JAEQRT000000000
**MF7836**	2020	M1	meat	food processing environment	ST1416	CC19	II	MF7335	PRJNA689484	SAMN17224650	SRR13588409	JAEQRS000000000
**MF7837**	2020	M1	meat	food processing environment	ST9	CC9	II	MF1576	PRJNA689484	SAMN17224651	SRR13588408	JAEQRR000000000
**MF7838**	2020	M1	meat	food processing environment	ST394	CC415	II	MF7713	PRJNA689484	SAMN17224652	SRR13588407	JAEQRP000000000
**MF7839**	2020	M1	meat	food processing environment	ST394	CC415	II	MF7713	PRJNA689484	SAMN17224653	SRR13588406	JAEQRO000000000
**MF7840**	2020	M1	meat	food processing environment	ST91	CC91	II	MF7663	PRJNA689484	SAMN17224654	SRR13588405	JAEQRN000000000
**MF7841**	2020	M1	meat	food processing environment	ST91	CC91	II	MF7663	PRJNA689484	SAMN17224655	SRR13588404	JAEQRM000000000
**MF7842**	2020	M1	meat	food processing environment	ST8	CC8	II	MF4245	PRJNA689484	SAMN17224656	SRR13588403	JAEQRL000000000
**MF7843**	2020	M1	meat	food processing - low hygienic zone	ST91	CC91	II	MF7663	PRJNA689484	SAMN17224657	SRR13588401	JAEQRK000000000
**MF7844**	2020	M1	meat	food processing - low hygienic zone	ST8	CC8	II	MF4245	PRJNA689484	SAMN17224658	SRR13588400	JAEQRJ000000000

**MF7845**	2020	M4	meat	food processing environment	ST1416	CC19	II	MF7335	PRJNA689484	SAMN17224659	SRR13378870	JAEQRI000000000
**MF7846**	2020	M4	meat	food processing environment	ST1416	CC19	II	MF7335	PRJNA689484	SAMN17224660	SRR13378869	JAEQRH000000000
**MF7847**	2020	M4	meat	food processing environment	ST394	CC415	II	MF7713	PRJNA689484	SAMN17224661	SRR13378868	JAEQRG000000000
**MF7848**	2020	M4	meat	food processing environment	ST394	CC415	II	MF7713	PRJNA689484	SAMN17224662	SRR13378867	JAEQRF000000000
**MF7849**	2020	M4	meat	food processing environment	ST394	CC415	II	MF7713	PRJNA689484	SAMN17224663	SRR13378866	JAEQRE000000000
**MF4245**	2001	S1	salmon	food processing environment	ST8	CC8	II	MF4245	PRJNA293674	SAMN04009325	SRR3099222	LKVA00000000
**MF3853**	2011	S4	salmon	food processing environment	ST31	CC31	II	MF3853	PRJNA689484	SAMN17224282	SRR13588276	JAERFT000000000
**MF3858**	2011	S4	salmon	food processing environment	ST121	CC121	II	MF4804	PRJNA689484	SAMN17224283	SRR13588265	JAERFS000000000
**MF3860**	2011	S4	salmon	food processing environment	ST20	CC20	II	MF7172	PRJNA689484	SAMN17224284	SRR13588254	JAERFR000000000
**MF3886**	2011	S1	salmon	food processing environment	ST7	CC7	II	MF2133	PRJNA689484	SAMN17224285	SRR13588095	JAERFQ000000000
**MF3887**	2011	S1	salmon	food processing environment	ST7	CC7	II	MF2133	PRJNA689484	SAMN17224286	SRR13588094	JAERFP000000000
**MF3904**	2011	S1	salmon	food processing environment	ST7	CC7	II	MF2133	PRJNA689484	SAMN17224287	SRR13588164	JAERFO000000000
**MF3912**	2011	S1	salmon	food processing environment	ST7	CC7	II	MF2133	PRJNA689484	SAMN17224288	SRR13588153	JAERFN000000000
**MF3939**	2011	S3	salmon	food processing environment	ST14	CC14	II	MF3939	PRJNA689484	SAMN17224289	SRR13588243	JAERFM000000000
**MF3949**	2011	S1	salmon	food processing environment	ST8	CC8	II	MF4245	PRJNA293674	SAMN04009326	SRR3099221	LKUZ00000000
**MF3992**	2011	S2	salmon	food processing environment	ST732	CC7	II	MF2133	PRJNA689484	SAMN17224290	SRR13588142	JAERFL000000000
**MF3993**	2011	S2	salmon	food processing environment	ST732	CC7	II	MF2133	PRJNA689484	SAMN17224291	SRR13588131	JAERFK000000000
**MF3994**	2011	S2	salmon	food processing environment	ST732	CC7	II	MF2133	PRJNA689484	SAMN17224292	SRR13588382	JAERFJ000000000
**MF3995**	2011	S2	salmon	food processing environment	ST732	CC7	II	MF2133	PRJNA689484	SAMN17224293	SRR13588232	JAERFI000000000
**MF3999**	2011	S2	salmon	food processing environment	ST14	CC14	II	MF3939	PRJNA689484	SAMN17224294	SRR13588380	JAERFH000000000
**MF4001**	2011	S2	salmon	food processing environment	ST14	CC14	II	MF3939	PRJNA689484	SAMN17224295	SRR13588221	JAERFG000000000
**MF4009**	2011	S2	salmon	raw material	ST551	CC8	II	MF4245	PRJNA689484	SAMN17224296	SRR13588379	JAERFF000000000
**MF4076**	2011	S1	salmon	food processing environment	ST8	CC8	II	MF4245	PRJNA689484	SAMN17224297	SRR13588120	JAERFE000000000
**MF4077**	2011	S1	salmon	food processing environment	ST8	CC8	II	MF4245	PRJNA293674	SAMN04404360	SRR3099223	LQXC00000000
**MF4085**	2011	S1	salmon	food processing environment	ST8	CC8	II	MF4245	PRJNA689484	SAMN17224298	SRR13588109	JAERFD000000000
**MF4455**	2011	S1	salmon	food processing environment	ST7	CC7	II	MF2133	PRJNA689484	SAMN17224303	SRR13588182	JAEREY000000000
**MF4464**	2011	S1	salmon	food processing environment	ST7	CC7	II	MF2133	PRJNA689484	SAMN17224304	SRR13588172	JAEREX000000000
**MF4466**	2011	S1	salmon	food processing environment	ST7	CC7	II	MF2133	PRJNA689484	SAMN17224305	SRR13588171	JAEREW000000000
**MF4382**	2012	S2	salmon	food processing environment	ST732	CC7	II	MF2133	PRJNA689484	SAMN17224299	SRR13588098	JAERFC000000000
**MF4388**	2012	S2	salmon	food processing environment	ST7	CC7	II	MF2133	PRJNA689484	SAMN17224300	SRR13588378	JAERFB000000000
**MF4417**	2012	S2	salmon	food processing environment	ST732	CC7	II	MF2133	PRJNA689484	SAMN17224301	SRR13588083	JAERFA000000000
**MF4420**	2012	S2	salmon	food processing environment	ST732	CC7	II	MF2133	PRJNA689484	SAMN17224302	SRR13588093	JAEREZ000000000
**MF4475**	2012	S3	salmon	food processing environment	ST14	CC14	II	MF3939	PRJNA689484	SAMN17224306	SRR13588210	JAEREV000000000
**MF4586**	2012	S1	salmon	food processing environment	ST14	CC14	II	MF3939	PRJNA689484	SAMN17224310	SRR13588310	JAERER000000000
**MF4588**	2012	S1	salmon	food processing environment	ST7	CC7	II	MF2133	PRJNA689484	SAMN17224311	SRR13588299	JAEREQ000000000
**MF4594**	2012	S1	salmon	food processing environment	ST177	CC177	II	MF7243	PRJNA689484	SAMN17224312	SRR13588170	JAEREP000000000
**MF4601**	2012	S1	salmon	food processing environment	ST177	CC177	II	MF7243	PRJNA689484	SAMN17224313	SRR13588169	JAEREO000000000
**MF4792**	2012	S2	salmon	food processing environment	ST31	CC31	II	MF3853	PRJNA689484	SAMN17224316	SRR13588281	JAEREL000000000
**MF4804**	2012	S2	salmon	food processing environment	ST121	CC121	II	MF4804	PRJNA689484	SAMN17224317	SRR13588280	JAEREK000000000
**MF5046**	2013	S1	salmon	food processing environment	ST7	CC7	II	MF2133	PRJNA689484	SAMN17224320	SRR13588167	JAEREH000000000
**MF5050**	2013	S1	salmon	food processing environment	ST7	CC7	II	MF2133	PRJNA689484	SAMN17224321	SRR13588166	JAEREG000000000
**MF5081**	2013	S1	salmon	food processing environment	ST7	CC7	II	MF2133	PRJNA689484	SAMN17224322	SRR13588165	JAEREF000000000
**MF5166**	2013	S1	salmon	food processing environment	ST7	CC7	II	MF2133	PRJNA689484	SAMN17224323	SRR13588163	JAEREE000000000
**MF5256**	2013	S1	salmon	food processing environment	ST7	CC7	II	MF2133	PRJNA689484	SAMN17224324	SRR13588376	JAERED000000000
**MF5257**	2013	S1	salmon	food processing environment	ST7	CC7	II	MF2133	PRJNA689484	SAMN17224325	SRR13588162	JAEREC000000000
**MF5259**	2013	S1	salmon	food processing environment	ST7	CC7	II	MF2133	PRJNA689484	SAMN17224326	SRR13588279	JAEREB000000000

**MF5415**	2014	S1	salmon	food processing environment	ST7	CC7	II	MF2133	PRJNA689484	SAMN17224335	SRR13588160	JAERDS000000000
**MF5445**	2014	S1	salmon	raw material	ST1607	CC91	II	MF7663	PRJNA689484	SAMN17224336	SRR13588372	JAERDR000000000
**MF7329**	2017	S6	salmon	food processing environment	ST2	CC2	I	MF7332	PRJNA689484	SAMN17224468	SRR13588315	JAEQYS000000000
**MF7330**	2017	S6	salmon	food processing environment	ST2	CC2	I	MF7332	PRJNA689484	SAMN17224469	SRR13588356	JAEQYR000000000
**MF7331**	2017	S6	salmon	food processing environment	ST2	CC2	I	MF7332	PRJNA689484	SAMN17224470	SRR13588355	JAEQYQ000000000
**MF7332**	2017	S6	salmon	food processing environment	ST2	CC2	I	MF7332	PRJNA689484	SAMN17224471	SRR13588354	JAEQYP000000000
**MF7333**	2017	S6	salmon	food product	ST2	CC2	I	MF7332	PRJNA689484	SAMN17224472	SRR13588353	JAEQYO000000000
**MF7335**	2017	S6	salmon	food processing environment	ST19	CC19	II	MF7335	PRJNA689484	SAMN17224473	SRR13588352	JAEQYN000000000
**MF7336**	2017	S6	salmon	food processing environment	ST2	CC2	I	MF7332	PRJNA689484	SAMN17224474	SRR13588351	JAEQYM000000000
**MF7337**	2017	S6	salmon	food processing environment	ST403	CC403	II	MF7380	PRJNA689484	SAMN17224475	SRR13588314	JAEQYL000000000
**MF7339**	2017	S6	salmon	food processing environment	ST2	CC2	I	MF7332	PRJNA689484	SAMN17224476	SRR13588313	JAEQYK000000000
**MF7341**	2017	S6	salmon	food processing environment	ST403	CC403	II	MF7380	PRJNA689484	SAMN17224477	SRR13588312	JAEQYJ000000000
**MF7345**	2017	S6	salmon	food processing environment	ST19	CC19	II	MF7335	PRJNA689484	SAMN17224478	SRR13588311	JAEQYI000000000
**MF7350**	2017	S6	salmon	food processing environment	ST249	CC315	I	MF7350	PRJNA689484	SAMN17224479	SRR13588350	JAEQYH000000000
**MF7354**	2017	S6	salmon	food processing environment	ST403	CC403	II	MF7380	PRJNA689484	SAMN17224480	SRR13588309	JAEQYG000000000
**MF7355**	2017	S6	salmon	food processing environment	ST403	CC403	II	MF7380	PRJNA689484	SAMN17224481	SRR13588308	JAEQYF000000000
**MF7360**	2017	S6	salmon	food processing environment	ST403	CC403	II	MF7380	PRJNA689484	SAMN17224482	SRR13588349	JAEQYE000000000
**MF7363**	2017	S6	salmon	food processing environment	ST19	CC19	II	MF7335	PRJNA689484	SAMN17224483	SRR13588347	JAEQYD000000000
**MF7366**	2017	S6	salmon	food processing environment	ST403	CC403	II	MF7380	PRJNA689484	SAMN17224484	SRR13588307	JAEQYC000000000
**MF7371**	2017	S6	salmon	food processing environment	ST19	CC19	II	MF7335	PRJNA689484	SAMN17224485	SRR13588346	JAEQYB000000000
**MF7372**	2017	S6	salmon	food processing environment	ST19	CC19	II	MF7335	PRJNA689484	SAMN17224486	SRR13588345	JAEQYA000000000
**MF7375**	2017	S6	salmon	food processing environment	ST8	CC8	II	MF4245	PRJNA689484	SAMN17224487	SRR13588306	JAEQXZ000000000
**MF7376**	2017	S6	salmon	food processing environment	ST249	CC315	I	MF7350	PRJNA689484	SAMN17224488	SRR13588344	JAEQXY000000000
**MF7380**	2017	S6	salmon	food processing environment	ST403	CC403	II	MF7380	PRJNA689484	SAMN17224489	SRR13588305	JAEQXX000000000
**MF7382**	2017	S6	salmon	food processing environment	ST403	CC403	II	MF7380	PRJNA689484	SAMN17224490	SRR13588304	JAEQXW000000000
**MF7386**	2017	S6	salmon	food processing environment	ST403	CC403	II	MF7380	PRJNA689484	SAMN17224492	SRR13588303	JAEQXU000000000
**MF6980**	2018	S2	salmon	food product	ST14	CC14	II	MF3939	PRJNA689484	SAMN17224385	SRR13588262	JAERBX000000000
**MF6981**	2018	S2	salmon	food processing environment	ST1	CC1	I	MF7036	PRJNA689484	SAMN17224386	SRR13588261	JAERBW000000000
**MF6982**	2018	S2	salmon	food processing environment	ST14	CC14	II	MF3939	PRJNA689484	SAMN17224387	SRR13588260	JAERBV000000000
**MF7025**	2018	S2	salmon	food processing environment	ST14	CC14	II	MF3939	PRJNA689484	SAMN17224389	SRR13588258	JAERBT000000000
**MF7026**	2018	S2	salmon	food product	ST1	CC1	I	MF7036	PRJNA689484	SAMN17224390	SRR13588257	JAERBS000000000
**MF7027**	2018	S2	salmon	food product	ST1	CC1	I	MF7036	PRJNA689484	SAMN17224391	SRR13588256	JAERBR000000000
**MF7028**	2018	S2	salmon	food processing environment	ST1	CC1	I	MF7036	PRJNA689484	SAMN17224392	SRR13588255	JAERBQ000000000
**MF7029**	2018	S2	salmon	food product	ST1	CC1	I	MF7036	PRJNA689484	SAMN17224393	SRR13588253	JAERBP000000000
**MF7030**	2018	S2	salmon	food product	ST1	CC1	I	MF7036	PRJNA689484	SAMN17224394	SRR13588252	JAERBO000000000
**MF7031**	2018	S2	salmon	food processing environment	ST14	CC14	II	MF3939	PRJNA689484	SAMN17224395	SRR13588251	JAERBN000000000
**MF7032**	2018	S2	salmon	food processing environment	ST1	CC1	I	MF7036	PRJNA689484	SAMN17224396	SRR13588250	JAERBM000000000
**MF7033**	2018	S2	salmon	food processing environment	ST14	CC14	II	MF3939	PRJNA689484	SAMN17224397	SRR13588249	JAERBL000000000
**MF7034**	2018	S2	salmon	food product	ST1	CC1	I	MF7036	PRJNA689484	SAMN17224398	SRR13588248	JAERBK000000000
**MF7035**	2018	S2	salmon	raw material	ST121	CC121	II	MF4804	PRJNA689484	SAMN17224399	SRR13588247	JAERBJ000000000
**MF7036**	2018	S2	salmon	food processing environment	ST1	CC1	I	MF7036	PRJNA689484	SAMN17224400	SRR13588246	JAERBI000000000
**MF7037**	2018	S2	salmon	food processing environment	ST1	CC1	I	MF7036	PRJNA689484	SAMN17224401	SRR13588245	JAERBH000000000
**MF7038**	2018	S2	salmon	food processing environment	ST14	CC14	II	MF3939	PRJNA689484	SAMN17224402	SRR13588244	JAERBG000000000
**MF7039**	2018	S2	salmon	food processing environment	ST1	CC1	I	MF7036	PRJNA689484	SAMN17224403	SRR13588242	JAERBF000000000
**MF7040**	2018	S2	salmon	food processing environment	ST1	CC1	I	MF7036	PRJNA689484	SAMN17224404	SRR13588241	JAERBE000000000
**MF7041**	2018	S2	salmon	food processing environment	ST14	CC14	II	MF3939	PRJNA689484	SAMN17224405	SRR13588240	JAERBD000000000

**MF7042**	2018	S2	salmon	food processing environment	ST1	CC1	I	MF7036	PRJNA689484	SAMN17224406	SRR13588239	JAERBC000000000
**MF7043**	2018	S2	salmon	food processing environment	ST1	CC1	I	MF7036	PRJNA689484	SAMN17224407	SRR13588238	JAERBB000000000
**MF7044**	2018	S2	salmon	food processing environment	ST732	CC7	II	MF2133	PRJNA689484	SAMN17224408	SRR13588237	JAERBA000000000
**MF7045**	2018	S2	salmon	food processing environment	ST1	CC1	I	MF7036	PRJNA689484	SAMN17224409	SRR13588236	JAERAZ000000000
**MF7064**	2018	S2	salmon	food processing environment	ST14	CC14	II	MF3939	PRJNA689484	SAMN17224410	SRR13588235	JAERAY000000000
**MF7065**	2018	S2	salmon	food processing environment	ST14	CC14	II	MF3939	PRJNA689484	SAMN17224411	SRR13588234	JAERAX000000000
**MF7066**	2018	S2	salmon	food processing environment	ST1	CC1	I	MF7036	PRJNA689484	SAMN17224412	SRR13588233	JAERAW000000000
**MF7067**	2018	S2	salmon	food processing environment	ST14	CC14	II	MF3939	PRJNA689484	SAMN17224413	SRR13588231	JAERAV000000000
**MF7068**	2018	S2	salmon	food processing environment	ST1	CC1	I	MF7036	PRJNA689484	SAMN17224414	SRR13588230	JAERAU000000000
**MF7069**	2018	S2	salmon	food processing environment	ST1	CC1	I	MF7036	PRJNA689484	SAMN17224415	SRR13588229	JAERAT000000000
**MF7072**	2018	S2	salmon	food processing environment	ST14	CC14	II	MF3939	PRJNA689484	SAMN17224416	SRR13588228	JAERAS000000000
**MF7074**	2018	S2	salmon	food processing environment	ST14	CC14	II	MF3939	PRJNA689484	SAMN17224417	SRR13588227	JAERAR000000000
**MF7098**	2018	S2	salmon	food processing environment	ST451	CC11	II	MF4627	PRJNA689484	SAMN17224418	SRR13588226	JAERAQ000000000
**MF7104**	2018	S2	salmon	food processing environment	ST1	CC1	I	MF7036	PRJNA689484	SAMN17224419	SRR13588366	JAERAP000000000
**MF7105**	2018	S2	salmon	food processing environment	ST1	CC1	I	MF7036	PRJNA689484	SAMN17224420	SRR13588225	JAERAO000000000
**MF7107**	2018	S2	salmon	food processing environment	ST1	CC1	I	MF7036	PRJNA689484	SAMN17224421	SRR13588365	JAERAN000000000
**MF7147**	2018	S2	salmon	raw material	ST20	CC20	II	MF7172	PRJNA689484	SAMN17224422	SRR13588364	JAERAM000000000
**MF7149**	2018	S2	salmon	raw material	ST1	CC1	I	MF7036	PRJNA689484	SAMN17224423	SRR13588224	JAERAL000000000
**MF7151**	2018	S2	salmon	food processing environment	ST296	CC88	I	MF7854	PRJNA689484	SAMN17224424	SRR13588363	JAERAK000000000
**MF7152**	2018	S2	salmon	raw material	ST1	CC1	I	MF7036	PRJNA689484	SAMN17224425	SRR13588223	JAERAJ000000000
**MF7156**	2018	S2	salmon	food processing environment	ST14	CC14	II	MF3939	PRJNA689484	SAMN17224426	SRR13588222	JAERAI000000000
**MF7161**	2018	S2	salmon	food processing environment	ST732	CC7	II	MF2133	PRJNA689484	SAMN17224427	SRR13588362	JAERAH000000000
**MF7172**	2018	S2	salmon	raw material	ST20	CC20	II	MF7172	PRJNA689484	SAMN17224430	SRR13588218	JAERAE000000000
**MF7242**	2018	S5	salmon	food processing environment	ST177	CC177	II	MF7243	PRJNA689484	SAMN17224441	SRR13588211	JAEQZT000000000
**MF7243**	2018	S5	salmon	food processing environment	ST177	CC177	II	MF7243	PRJNA689484	SAMN17224442	SRR13588209	JAEQZS000000000
**MF7244**	2018	S5	salmon	food processing environment	ST177	CC177	II	MF7243	PRJNA689484	SAMN17224443	SRR13588208	JAEQZR000000000
**MF7245**	2018	S5	salmon	food processing environment	ST177	CC177	II	MF7243	PRJNA689484	SAMN17224444	SRR13588207	JAEQZQ000000000
**MF7260**	2018	S5	salmon	food processing environment	ST177	CC177	II	MF7243	PRJNA689484	SAMN17224445	SRR13588206	JAEQZP000000000
**MF7384**	2018	S6	salmon	food processing environment	ST19	CC19	II	MF7335	PRJNA689484	SAMN17224491	SRR13588343	JAEQXV000000000
**MF7387**	2018	S6	salmon	food processing environment	ST403	CC403	II	MF7380	PRJNA689484	SAMN17224493	SRR13588342	JAEQXT000000000
**MF7164**	2019	S2	salmon	food processing environment	ST14	CC14	II	MF3939	PRJNA689484	SAMN17224428	SRR13588220	JAERAG000000000
**MF7170**	2019	S2	salmon	food processing environment	ST1	CC1	I	MF7036	PRJNA689484	SAMN17224429	SRR13588219	JAERAF000000000
**MF7176**	2019	S2	salmon	food processing environment	ST1	CC1	I	MF7036	PRJNA689484	SAMN17224431	SRR13588217	JAERAD000000000
**MF7177**	2019	S2	salmon	food processing environment	ST1	CC1	I	MF7036	PRJNA689484	SAMN17224432	SRR13588216	JAERAC000000000
**MF7223**	2019	S2	salmon	food processing environment	ST732	CC7	II	MF2133	PRJNA689484	SAMN17224433	SRR13588361	JAERAB000000000
**MF7224**	2019	S2	salmon	food processing environment	ST732	CC7	II	MF2133	PRJNA689484	SAMN17224434	SRR13588360	JAERAA000000000
**MF7227**	2019	S2	salmon	food product	ST1	CC1	I	MF7036	PRJNA689484	SAMN17224435	SRR13588215	JAEQZZ000000000
**MF7228**	2019	S2	salmon	food processing environment	ST1	CC1	I	MF7036	PRJNA689484	SAMN17224436	SRR13588214	JAEQZY000000000
**MF7231**	2019	S2	salmon	food processing environment	ST732	CC7	II	MF2133	PRJNA689484	SAMN17224437	SRR13588358	JAEQZX000000000
**MF7235**	2019	S2	salmon	food processing environment	ST732	CC7	II	MF2133	PRJNA689484	SAMN17224438	SRR13588213	JAEQZW000000000
**MF7236**	2019	S2	salmon	food processing environment	ST1	CC1	I	MF7036	PRJNA689484	SAMN17224439	SRR13588212	JAEQZV000000000
**MF7240**	2019	S2	salmon	food processing environment	ST1	CC1	I	MF7036	PRJNA689484	SAMN17224440	SRR13588357	JAEQZU000000000
**MF7262**	2019	S5	salmon	food processing environment	ST177	CC177	II	MF7243	PRJNA689484	SAMN17224446	SRR13588205	JAEQZO000000000
**MF7277**	2019	S5	salmon	food processing environment	ST177	CC177	II	MF7243	PRJNA689484	SAMN17224448	SRR13588204	JAEQZM000000000
**MF7282**	2019	S5	salmon	food processing environment	ST177	CC177	II	MF7243	PRJNA689484	SAMN17224449	SRR13588203	JAEQZL000000000
**MF7286**	2019	S5	salmon	food processing environment	ST177	CC177	II	MF7243	PRJNA689484	SAMN17224450	SRR13588202	JAEQZK000000000

**MF7287**	2019	S5	salmon	food processing environment	ST177	CC177	II	MF7243	PRJNA689484	SAMN17224451	SRR13588201	JAEQZJ000000000
**MF7288**	2019	S5	salmon	food processing environment	ST177	CC177	II	MF7243	PRJNA689484	SAMN17224452	SRR13588200	JAEQZI000000000
**MF7295**	2019	S5	salmon	food processing environment	ST177	CC177	II	MF7243	PRJNA689484	SAMN17224453	SRR13588198	JAEQZH000000000
**MF7298**	2019	S5	salmon	food processing environment	ST177	CC177	II	MF7243	PRJNA689484	SAMN17224454	SRR13588197	JAEQZG000000000
**MF7301**	2019	S5	salmon	food processing environment	ST177	CC177	II	MF7243	PRJNA689484	SAMN17224455	SRR13588196	JAEQZF000000000
**MF7306**	2019	S5	salmon	food processing environment	ST177	CC177	II	MF7243	PRJNA689484	SAMN17224456	SRR13588195	JAEQZE000000000
**MF7308**	2019	S5	salmon	food processing environment	ST177	CC177	II	MF7243	PRJNA689484	SAMN17224457	SRR13588194	JAEQZD000000000
**MF7309**	2019	S5	salmon	food processing environment	ST177	CC177	II	MF7243	PRJNA689484	SAMN17224458	SRR13588193	JAEQZC000000000
**MF7310**	2019	S5	salmon	food processing environment	ST177	CC177	II	MF7243	PRJNA689484	SAMN17224459	SRR13588192	JAEQZB000000000
**MF7314**	2019	S5	salmon	food processing environment	ST177	CC177	II	MF7243	PRJNA689484	SAMN17224460	SRR13588322	JAEQZA000000000
**MF7315**	2019	S5	salmon	food processing environment	ST121	CC121	II	MF4804	PRJNA689484	SAMN17224461	SRR13588321	JAEQYZ000000000
**MF7388**	2019	S6	salmon	food processing environment	ST121	CC121	II	MF4804	PRJNA689484	SAMN17224494	SRR13588341	JAEQXS000000000
**MF7389**	2019	S6	salmon	food processing environment	ST121	CC121	II	MF4804	PRJNA689484	SAMN17224495	SRR13588340	JAEQXR000000000
**MF7390**	2019	S6	salmon	food processing environment	ST121	CC121	II	MF4804	PRJNA689484	SAMN17224496	SRR13588339	JAEQXQ000000000
**MF7391**	2019	S6	salmon	food processing environment	ST121	CC121	II	MF4804	PRJNA689484	SAMN17224497	SRR13588338	JAEQXP000000000
**MF7392**	2019	S6	salmon	food processing environment	ST121	CC121	II	MF4804	PRJNA689484	SAMN17224498	SRR13588302	JAEQXO000000000
**MF7393**	2019	S6	salmon	raw material	ST121	CC121	II	MF4804	PRJNA689484	SAMN17224499	SRR13588336	JAEQXN000000000
**MF7394**	2019	S6	salmon	food processing environment	ST403	CC403	II	MF7380	PRJNA689484	SAMN17224500	SRR13588301	JAEQXM000000000
**MF7395**	2019	S6	salmon	food processing environment	ST121	CC121	II	MF4804	PRJNA689484	SAMN17224501	SRR13588300	JAEQXL000000000
**MF7396**	2019	S6	salmon	food product	ST121	CC121	II	MF4804	PRJNA689484	SAMN17224502	SRR13588298	JAEQXK000000000
**MF7397**	2019	S6	salmon	raw material	ST121	CC121	II	MF4804	PRJNA689484	SAMN17224503	SRR13588335	JAEQXJ000000000
**MF7398**	2019	S6	salmon	food processing environment	ST121	CC121	II	MF4804	PRJNA689484	SAMN17224504	SRR13588297	JAEQXI000000000
**MF7399**	2019	S6	salmon	food processing environment	ST121	CC121	II	MF4804	PRJNA689484	SAMN17224505	SRR13588296	JAEQXH000000000
**MF7400**	2019	S6	salmon	food processing environment	ST121	CC121	II	MF4804	PRJNA689484	SAMN17224506	SRR13588295	JAEQXG000000000
**MF7694**	2019	-	salmon	food product	ST20	CC20	II	MF7172	PRJNA689484	SAMN17224563	SRR13588440	JAEQVB000000000
**MF7695**	2019	S6	salmon	food processing environment	ST249	CC315	I	MF7350	PRJNA689484	SAMN17224564	SRR13588107	JAEQVA000000000
**MF7696**	2019	S6	salmon	food processing environment	ST121	CC121	II	MF4804	PRJNA689484	SAMN17224565	SRR13588106	JAEQUZ000000000
**MF7697**	2019	S6	salmon	food processing environment	ST121	CC121	II	MF4804	PRJNA689484	SAMN17224566	SRR13588105	JAEQUY000000000
**MF7698**	2019	S6	salmon	food processing environment	ST121	CC121	II	MF4804	PRJNA689484	SAMN17224567	SRR13588104	JAEQUX000000000
**MF7699**	2019	S6	salmon	raw material	ST121	CC121	II	MF4804	PRJNA689484	SAMN17224568	SRR13588103	JAEQUW000000000
**MF7700**	2019	S6	salmon	food processing environment	ST121	CC121	II	MF4804	PRJNA689484	SAMN17224569	SRR13588102	JAEQUV000000000
**MF7701**	2019	S6	salmon	food processing environment	ST394	CC415	II	MF7713	PRJNA689484	SAMN17224570	SRR13588101	JAEQUU000000000
**MF7702**	2019	S6	salmon	food processing environment	ST403	CC403	II	MF7380	PRJNA689484	SAMN17224571	SRR13588100	JAEQUT000000000
**MF7703**	2019	S6	salmon	food processing environment	ST394	CC415	II	MF7713	PRJNA689484	SAMN17224572	SRR13588099	JAEQUS000000000
**MF7704**	2019	S6	salmon	food processing environment	ST394	CC415	II	MF7713	PRJNA689484	SAMN17224573	SRR13588097	JAEQUR000000000
**MF7705**	2019	S6	salmon	food processing environment	ST121	CC121	II	MF4804	PRJNA689484	SAMN17224574	SRR13588096	JAEQUQ000000000
**MF7706**	2019	S6	salmon	raw material	ST394	CC415	II	MF7713	PRJNA689484	SAMN17224575	SRR13588091	JAEQUP000000000
**MF7707**	2019	S6	salmon	food processing environment	ST121	CC121	II	MF4804	PRJNA689484	SAMN17224576	SRR13588090	JAEQUO000000000
**MF7708**	2019	S6	salmon	food processing environment	ST403	CC403	II	MF7380	PRJNA689484	SAMN17224577	SRR13588089	JAEQUN000000000
**MF7709**	2019	S6	salmon	food processing environment	ST121	CC121	II	MF4804	PRJNA689484	SAMN17224578	SRR13588088	JAEQUM000000000
**MF7710**	2019	S6	salmon	food processing environment	ST394	CC415	II	MF7713	PRJNA689484	SAMN17224579	SRR13588087	JAEQUL000000000
**MF7711**	2019	S6	salmon	food processing environment	ST121	CC121	II	MF4804	PRJNA689484	SAMN17224580	SRR13588086	JAEQUK000000000
**MF7712**	2019	S6	salmon	food processing environment	ST403	CC403	II	MF7380	PRJNA689484	SAMN17224581	SRR13588085	JAEQUJ000000000
**MF7713**	2019	S6	salmon	food processing environment	ST394	CC415	II	MF7713	PRJNA689484	SAMN17224582	SRR13588084	JAEQUI000000000
**MF7714**	2019	S6	salmon	food processing environment	ST121	CC121	II	MF4804	PRJNA689484	SAMN17224583	SRR13588082	JAEQUH000000000
**MF7715**	2019	S6	salmon	raw material	ST121	CC121	II	MF4804	PRJNA689484	SAMN17224584	SRR13588081	JAEQUG000000000

**MF7716**	2019	S6	salmon	raw material	ST121	CC121	II	MF4804	PRJNA689484	SAMN17224585	SRR13588080	JAEQUF000000000
**MF7717**	2019	S6	salmon	raw material	ST121	CC121	II	MF4804	PRJNA689484	SAMN17224586	SRR13588079	JAEQUE000000000
**MF7718**	2019	S6	salmon	food processing environment	ST19	CC19	II	MF7335	PRJNA689484	SAMN17224587	SRR13588078	JAEQUD000000000
**MF7719**	2019	S6	salmon	food processing environment	ST121	CC121	II	MF4804	PRJNA689484	SAMN17224588	SRR13588077	JAEQUC000000000
**MF7720**	2019	S6	salmon	food processing environment	ST394	CC415	II	MF7713	PRJNA689484	SAMN17224589	SRR13588076	JAEQUB000000000
**MF7721**	2019	S6	salmon	food processing environment	ST121	CC121	II	MF4804	PRJNA689484	SAMN17224590	SRR13588075	JAEQUA000000000
**MF7722**	2019	S6	salmon	food product	ST121	CC121	II	MF4804	PRJNA689484	SAMN17224591	SRR13588074	JAEQTZ000000000
**MF7723**	2019	S6	salmon	food product	ST121	CC121	II	MF4804	PRJNA689484	SAMN17224592	SRR13588073	JAEQTY000000000
**MF7724**	2019	S6	salmon	raw material	ST121	CC121	II	MF4804	PRJNA689484	SAMN17224593	SRR13588092	JAEQTX000000000
**MF7725**	2019	S6	salmon	food processing environment	ST121	CC121	II	MF4804	PRJNA689484	SAMN17224594	SRR13588191	JAEQTW000000000
**MF7726**	2019	S6	salmon	food processing environment	ST19	CC19	II	MF7335	PRJNA689484	SAMN17224595	SRR13588190	JAEQTV000000000
**MF7727**	2019	S6	salmon	food processing environment	ST394	CC415	II	MF7713	PRJNA689484	SAMN17224596	SRR13588189	JAEQTU000000000
**MF7728**	2019	S6	salmon	food processing environment	ST394	CC415	II	MF7713	PRJNA689484	SAMN17224597	SRR13588188	JAEQTT000000000
**MF7729**	2019	S6	salmon	food processing environment	ST121	CC121	II	MF4804	PRJNA689484	SAMN17224598	SRR13588187	JAEQTS000000000
**MF7730**	2019	S6	salmon	food processing environment	ST31	CC31	II	MF3853	PRJNA689484	SAMN17224599	SRR13588186	JAEQTR000000000
**MF7731**	2019	S6	salmon	food processing environment	ST394	CC415	II	MF7713	PRJNA689484	SAMN17224600	SRR13588185	JAEQTQ000000000
**MF7732**	2019	S6	salmon	food processing environment	ST394	CC415	II	MF7713	PRJNA689484	SAMN17224601	SRR13588184	JAEQTP000000000
**MF7733**	2019	S6	salmon	food processing environment	ST394	CC415	II	MF7713	PRJNA689484	SAMN17224602	SRR13588183	JAEQTO000000000
**MF7734**	2019	S6	salmon	food processing environment	ST19	CC19	II	MF7335	PRJNA689484	SAMN17224603	SRR13588181	JAEQTN000000000
**MF7735**	2019	S6	salmon	food product	ST394	CC415	II	MF7713	PRJNA689484	SAMN17224604	SRR13588180	JAEQTM000000000
**MF7736**	2019	S6	salmon	food processing environment	ST121	CC121	II	MF4804	PRJNA689484	SAMN17224605	SRR13588179	JAEQTL000000000
**MF7737**	2019	S6	salmon	raw material	ST394	CC415	II	MF7713	PRJNA689484	SAMN17224606	SRR13588178	JAEQTK000000000
**MF7738**	2019	S6	salmon	food product	ST394	CC415	II	MF7713	PRJNA689484	SAMN17224607	SRR13588177	JAEQTJ000000000
**MF7739**	2019	S6	salmon	food product	ST1	CC1	I	MF7036	PRJNA689484	SAMN17224608	SRR13588176	JAEQTI000000000
**MF7740**	2019	S6	salmon	food processing environment	ST403	CC403	II	MF7380	PRJNA689484	SAMN17224609	SRR13588175	JAEQTH000000000
**MF7741**	2019	S6	salmon	raw material	ST121	CC121	II	MF4804	PRJNA689484	SAMN17224610	SRR13588174	JAEQTG000000000
**MF7742**	2019	S6	salmon	raw material	ST121	CC121	II	MF4804	PRJNA689484	SAMN17224611	SRR13588173	JAEQTF000000000
**MF7743**	2019	S6	salmon	raw material	ST121	CC121	II	MF4804	PRJNA689484	SAMN17224612	SRR13588439	JAEQTE000000000
**MF7850**	2019	S6	salmon	food processing environment	ST37	CC37	II	MF7858	PRJNA689484	SAMN17224664	SRR13378865	JAEQRD000000000
**MF7851**	2019	S6	salmon	raw material	ST37	CC37	II	MF7858	PRJNA689484	SAMN17224665	SRR13378864	JAEQRC000000000
**MF7852**	2019	S6	salmon	raw material	ST37	CC37	II	MF7858	PRJNA689484	SAMN17224666	SRR13378863	JAEQRB000000000
**MF7853**	2019	S6	salmon	food processing environment	ST249	CC315	I	MF7350	PRJNA689484	SAMN17224667	SRR13378862	JAEQRA000000000
**MF7854**	2019	S6	salmon	raw material	ST296	CC88	I	MF7854	PRJNA689484	SAMN17224668	SRR13378860	JAEQQZ000000000
**MF7855**	2019	S6	salmon	food processing environment	ST19	CC19	II	MF7335	PRJNA689484	SAMN17224669	SRR13378859	JAEQQY000000000
**MF7856**	2019	S6	salmon	food processing environment	ST249	CC315	I	MF7350	PRJNA689484	SAMN17224670	SRR13378858	JAEQQX000000000
**MF7857**	2019	S6	salmon	raw material	ST121	CC121	II	MF4804	PRJNA689484	SAMN17224671	SRR13378857	JAEQQW000000000
**MF7858**	2019	S6	salmon	food processing environment	ST37	CC37	II	MF7858	PRJNA689484	SAMN17224672	SRR13378856	JAEQQV000000000
**MF7859**	2019	S6	salmon	food processing environment	ST37	CC37	II	MF7858	PRJNA689484	SAMN17224673	SRR13378855	JAEQQU000000000
**MF7860**	2019	S6	salmon	food processing environment	ST37	CC37	II	MF7858	PRJNA689484	SAMN17224674	SRR13378854	JAEQQT000000000
**MF7861**	2019	S6	salmon	food processing environment	ST37	CC37	II	MF7858	PRJNA689484	SAMN17224675	SRR13378853	JAEQQS000000000
**MF7862**	2019	S6	salmon	food processing environment	ST37	CC37	II	MF7858	PRJNA689484	SAMN17224676	SRR13378852	JAEQQR000000000
**MF7863**	2019	S6	salmon	food processing environment	ST37	CC37	II	MF7858	PRJNA689484	SAMN17224677	SRR13378851	JAEQQQ000000000
**MF7864**	2019	S6	salmon	food processing environment	ST37	CC37	II	MF7858	PRJNA689484	SAMN17224678	SRR13378849	JAEQQP000000000
**MF7866**	2019	S6	salmon	raw material	ST37	CC37	II	MF7858	PRJNA689484	SAMN17224679	SRR13378848	JAEQQO000000000
**MF7867**	2019	S6	salmon	food processing environment	ST37	CC37	II	MF7858	PRJNA689484	SAMN17224680	SRR13378847	JAEQQN000000000
**MF7870**	2019	S6	salmon	food processing environment	ST403	CC403	II	MF7380	PRJNA689484	SAMN17224681	SRR13378846	JAEQQM000000000

**MF7871**	2019	S6	salmon	food processing environment	ST19	CC19	II	MF7335	PRJNA689484	SAMN17224682	SRR13378845	JAEQQL000000000
**MF7873**	2019	S6	salmon	food processing environment	ST19	CC19	II	MF7335	PRJNA689484	SAMN17224683	SRR13378844	JAEQQK000000000
**MF7876**	2019	S6	salmon	raw material	ST8	CC8	II	MF4245	PRJNA689484	SAMN17224684	SRR13378843	JAEQQJ000000000
**MF7878**	2020	S6	salmon	food processing environment	ST3	CC3	I	MF7891	PRJNA689484	SAMN17224685	SRR13378842	JAEQQI000000000
**MF7880**	2020	S6	salmon	raw material	ST8	CC8	II	MF4245	PRJNA689484	SAMN17224686	SRR13378841	JAEQQH000000000
**MF7881**	2020	S6	salmon	food product	ST19	CC19	II	MF7335	PRJNA689484	SAMN17224687	SRR13378840	JAEQQG000000000
**MF7883**	2020	S6	salmon	food processing environment	ST403	CC403	II	MF7380	PRJNA689484	SAMN17224688	SRR13378838	JAEQQF000000000
**MF7884**	2020	S6	salmon	food processing environment	ST403	CC403	II	MF7380	PRJNA689484	SAMN17224689	SRR13378837	JAEQQE000000000
**MF7885**	2020	S6	salmon	food product	ST19	CC19	II	MF7335	PRJNA689484	SAMN17224690	SRR13378836	JAEQQD000000000
**MF7887**	2020	S6	salmon	raw material	ST18	CC18	II	MF4566	PRJNA689484	SAMN17224691	SRR13378835	JAEQQC000000000
**MF7889**	2020	S6	salmon	food processing environment	ST3	CC3	I	MF7891	PRJNA689484	SAMN17224692	SRR13378834	JAEQQB000000000
**MF7890**	2020	S6	salmon	food processing environment	ST249	CC315	I	MF7350	PRJNA689484	SAMN17224693	SRR13378833	JAEQQA000000000
**MF7891**	2020	S6	salmon	food processing environment	ST3	CC3	I	MF7891	PRJNA689484	SAMN17224694	SRR13378832	JAEQPZ000000000
**MF7892**	2020	S6	salmon	food product	ST3	CC3	I	MF7891	PRJNA689484	SAMN17224695	SRR13378831	JAEQPY000000000
**MF7896**	2020	S6	salmon	food processing environment	ST3	CC3	I	MF7891	PRJNA689484	SAMN17224696	SRR13378830	JAEQPX000000000
**MF7897**	2020	S6	salmon	raw material	ST19	CC19	II	MF7335	PRJNA689484	SAMN17224697	SRR13378829	JAEQPW000000000
**MF7899**	2020	S6	salmon	raw material	ST19	CC19	II	MF7335	PRJNA689484	SAMN17224698	SRR13378827	JAEQPV000000000
**MF7900**	2020	S6	salmon	raw material	ST19	CC19	II	MF7335	PRJNA689484	SAMN17224699	SRR13378826	JAEQPU000000000
**MF7901**	2020	S6	salmon	food product	ST3	CC3	I	MF7891	PRJNA689484	SAMN17224700	SRR13378825	JAEQPT000000000
**MF7904**	2020	S6	salmon	food processing environment	ST403	CC403	II	MF7380	PRJNA689484	SAMN17224701	SRR13378824	JAEQPS000000000
**MF7905**	2020	S6	salmon	food processing environment	ST403	CC403	II	MF7380	PRJNA689484	SAMN17224702	SRR13378823	JAEQPR000000000
**MF7906**	2020	S6	salmon	food processing environment	ST3	CC3	I	MF7891	PRJNA689484	SAMN17224703	SRR13378822	JAEQPQ000000000
**MF7908**	2020	S6	salmon	food product	ST3	CC3	I	MF7891	PRJNA689484	SAMN17224704	SRR13378821	JAEQPP000000000
**MF7910**	2020	S6	salmon	food processing environment	ST249	CC315	I	MF7350	PRJNA689484	SAMN17224705	SRR13378820	JAEQPO000000000
**MF7912**	2020	S6	salmon	food processing environment	ST3	CC3	I	MF7891	PRJNA689484	SAMN17224706	SRR13378819	JAEQPN000000000
**MF7913**	2020	S6	salmon	food processing environment	ST3	CC3	I	MF7891	PRJNA689484	SAMN17224707	SRR13378818	JAEQPM000000000
**MF7914**	2020	S6	salmon	food processing environment	ST403	CC403	II	MF7380	PRJNA689484	SAMN17224708	SRR13378816	JAEQPL000000000
**MF7915**	2020	S6	salmon	food processing environment	ST403	CC403	II	MF7380	PRJNA689484	SAMN17224709	SRR13378815	JAEQPK000000000
**MF7916**	2020	S6	salmon	food processing environment	ST3	CC3	I	MF7891	PRJNA689484	SAMN17224710	SRR13378814	JAEQPJ000000000
**MF7918**	2020	S6	salmon	food processing environment	ST121	CC121	II	MF4804	PRJNA689484	SAMN17224711	SRR13378813	JAEQPI000000000
**MF7919**	2020	S6	salmon	food processing environment	ST121	CC121	II	MF4804	PRJNA689484	SAMN17224712	SRR13378812	JAEQPH000000000
**MF7920**	2020	S6	salmon	food processing environment	ST3	CC3	I	MF7891	PRJNA689484	SAMN17224713	SRR13378811	JAEQPG000000000
**MF7921**	2020	S6	salmon	food processing environment	ST19	CC19	II	MF7335	PRJNA689484	SAMN17224714	SRR13378810	JAEQPF000000000
**MF7922**	2020	S6	salmon	food processing environment	ST19	CC19	II	MF7335	PRJNA689484	SAMN17224715	SRR13378809	JAEQPE000000000
**MF7923**	2020	S6	salmon	food processing environment	ST19	CC19	II	MF7335	PRJNA689484	SAMN17224716	SRR13378808	JAEQPD000000000
**MF7924**	2020	S6	salmon	food processing environment	ST249	CC315	I	MF7350	PRJNA689484	SAMN17224717	SRR13378807	JAEQPC000000000
**MF7926**	2020	S6	salmon	food processing environment	ST3	CC3	I	MF7891	PRJNA689484	SAMN17224718	SRR13378805	JAEQPB000000000
**MF7928**	2020	S6	salmon	raw material	ST121	CC121	II	MF4804	PRJNA689484	SAMN17224719	SRR13378804	JAEQPA000000000
**MF7930**	2020	S6	salmon	raw material	ST3	CC3	I	MF7891	PRJNA689484	SAMN17224720	SRR13378803	JAEQOZ000000000
**MF7932**	2020	S6	salmon	food processing environment	ST121	CC121	II	MF4804	PRJNA689484	SAMN17224721	SRR13378802	JAEQOY000000000
**MF7934**	2020	S6	salmon	raw material	ST19	CC19	II	MF7335	PRJNA689484	SAMN17224722	SRR13378801	JAEQOX000000000
**MF7935**	2020	S6	salmon	food processing environment	ST3	CC3	I	MF7891	PRJNA689484	SAMN17224723	SRR13378800	JAEQOW000000000
**MF7936**	2020	S6	salmon	food processing environment	ST19	CC19	II	MF7335	PRJNA689484	SAMN17224724	SRR13378799	JAEQOV000000000
**MF7937**	2020	S6	salmon	food processing environment	ST19	CC19	II	MF7335	PRJNA689484	SAMN17224725	SRR13378798	JAEQOU000000000
**MF7938**	2020	S6	salmon	food processing environment	ST3	CC3	I	MF7891	PRJNA689484	SAMN17224726	SRR13378797	JAEQOT000000000
**MF7939**	2020	S6	salmon	food processing environment	ST249	CC315	I	MF7350	PRJNA689484	SAMN17224727	SRR13378796	JAEQOS000000000

**MF7941**	2020	S6	salmon	food product	ST19	CC19	II	MF7335	PRJNA689484	SAMN17224728	SRR13378928	JAEQOR000000000
**MF7943**	2020	S6	salmon	food product	ST19	CC19	II	MF7335	PRJNA689484	SAMN17224729	SRR13378927	JAEQOQ000000000
**MF7945**	2020	S6	salmon	food product	ST19	CC19	II	MF7335	PRJNA689484	SAMN17224730	SRR13378926	JAEQOP000000000
**MF7948**	2020	S6	salmon	food processing environment	ST3	CC3	I	MF7891	PRJNA689484	SAMN17224731	SRR13378925	JAEQOO000000000
**MF7949**	2020	S6	salmon	food processing environment	ST121	CC121	II	MF4804	PRJNA689484	SAMN17224732	SRR13378924	JAEQON000000000
**MF7951**	2020	S6	salmon	food processing environment	ST403	CC403	II	MF7380	PRJNA689484	SAMN17224733	SRR13378923	JAEQOM000000000
**MF7952**	2020	S6	salmon	food processing environment	ST403	CC403	II	MF7380	PRJNA689484	SAMN17224734	SRR13378922	JAEQOL000000000
**MF7953**	2020	S6	salmon	food processing environment	ST3	CC3	I	MF7891	PRJNA689484	SAMN17224735	SRR13378921	JAEQOK000000000
**MF7954**	2020	S6	salmon	food processing environment	ST3	CC3	I	MF7891	PRJNA689484	SAMN17224736	SRR13378920	JAEQOJ000000000
**MF7955**	2020	S6	salmon	raw material	ST121	CC121	II	MF4804	PRJNA689484	SAMN17224737	SRR13378919	JAEQOI000000000
**MF7957**	2020	S6	salmon	raw material	ST121	CC121	II	MF4804	PRJNA689484	SAMN17224738	SRR13378917	JAEQOH000000000
**MF7959**	2020	S6	salmon	raw material	ST121	CC121	II	MF4804	PRJNA689484	SAMN17224739	SRR13378916	JAEQOG000000000
**MF7961**	2020	S6	salmon	raw material	ST121	CC121	II	MF4804	PRJNA689484	SAMN17224740	SRR13378915	JAEQOF000000000
**MF7963**	2020	S6	salmon	raw material	ST121	CC121	II	MF4804	PRJNA689484	SAMN17224741	SRR13378914	JAEQOE000000000
**MF7965**	2020	S6	salmon	raw material	ST121	CC121	II	MF4804	PRJNA689484	SAMN17224742	SRR13378913	JAEQOD000000000
**MF7967**	2020	S6	salmon	food processing environment	ST403	CC403	II	MF7380	PRJNA689484	SAMN17224743	SRR13378912	JAEQOC000000000
**MF7968**	2020	S6	salmon	food processing environment	ST403	CC403	II	MF7380	PRJNA689484	SAMN17224744	SRR13378911	JAEQOB000000000
**MF7969**	2020	S6	salmon	food processing environment	ST3	CC3	I	MF7891	PRJNA689484	SAMN17224745	SRR13378910	JAEQOA000000000
**MF7971**	2020	S6	salmon	food processing environment	ST249	CC315	I	MF7350	PRJNA689484	SAMN17224746	SRR13378909	JAEQNZ000000000
**MF7973**	2020	S6	salmon	food processing environment	ST3	CC3	I	MF7891	PRJNA689484	SAMN17224747	SRR13378908	JAEQNY000000000
**MF7975**	2020	S6	salmon	food product	ST3	CC3	I	MF7891	PRJNA689484	SAMN17224748	SRR13378906	JAEQNX000000000
**MF7977**	2020	S6	salmon	raw material	ST121	CC121	II	MF4804	PRJNA689484	SAMN17224749	SRR13378905	JAEQNW000000000
**MF7979**	2020	S6	salmon	raw material	ST121	CC121	II	MF4804	PRJNA689484	SAMN17224750	SRR13378904	JAEQNV000000000
**MF7981**	2020	S6	salmon	raw material	ST121	CC121	II	MF4804	PRJNA689484	SAMN17224751	SRR13378903	JAEQNU000000000
**MF7983**	2020	S6	salmon	food product	ST121	CC121	II	MF4804	PRJNA689484	SAMN17224752	SRR13378902	JAEQNT000000000
**MF7985**	2020	S6	salmon	raw material	ST121	CC121	II	MF4804	PRJNA689484	SAMN17224753	SRR13378901	JAEQNS000000000
**MF7986**	2020	S6	salmon	raw material	ST121	CC121	II	MF4804	PRJNA689484	SAMN17224754	SRR13378900	JAEQNR000000000
**MF7988**	2020	S6	salmon	food processing environment	ST37	CC37	II	MF7858	PRJNA689484	SAMN17224755	SRR13378899	JAEQNQ000000000
**MF7989**	2020	S6	salmon	food processing environment	ST403	CC403	II	MF7380	PRJNA689484	SAMN17224756	SRR13378898	JAEQNP000000000
**MF7990**	2020	S6	salmon	food processing environment	ST3	CC3	I	MF7891	PRJNA689484	SAMN17224757	SRR13378897	JAEQNO000000000
**MF7991**	2020	S6	salmon	raw material	ST121	CC121	II	MF4804	PRJNA689484	SAMN17224758	SRR13378895	JAEQNN000000000
**MF7993**	2020	S6	salmon	raw material	ST121	CC121	II	MF4804	PRJNA689484	SAMN17224759	SRR13378894	JAEQNM000000000
**MF7995**	2020	S6	salmon	raw material	ST121	CC121	II	MF4804	PRJNA689484	SAMN17224760	SRR13378893	JAEQNL000000000
**MF7997**	2020	S6	salmon	food product	ST121	CC121	II	MF4804	PRJNA689484	SAMN17224761	SRR13378892	JAEQNK000000000
**MF7999**	2020	S6	salmon	food product	ST121	CC121	II	MF4804	PRJNA689484	SAMN17224762	SRR13378891	JAEQNJ000000000
**MF8001**	2020	S6	salmon	raw material	ST121	CC121	II	MF4804	PRJNA689484	SAMN17224763	SRR13378890	JAEQNI000000000

*Genomes from PRJNA293674 and PRJNA419519 were previously published [21, 59].

Table S2: Number of isolates from each processing plant

		No of isolates from meat processing plants									Salmon processing plants						other sources/ processing plants	Grand total
		M1	M2	M3	M4	M5	M6	M7	M8	M9	S1	S2	S3	S4	S5	S6		
lineage I	CC1			2								30				1	1	34
	CC2															7		7
	CC3															23	1	24
	CC4				1													1
	CC5						13											13
	CC6				1					2								3
	CC88											1				1		2
	CC220	1								1								2
	CC315															10		10
lineage II	CC7		12	4	2		15	3			16	14					2	68
	CC8	6	1				2	1			5	1				3		19
	CC9	115		8	141	4		3	14	4							1	290
	CC11	3			3				1	1		1						9
	CC14	1									1	16	2					20
	CC18	2						1								1	1	5
	CC19	3			25		1									26		55
	CC20											2		1			1	4
	CC21			1						1								2
	CC31											1		1		1		3
	CC37	1														13		14
	CC91	12								1	1							14
	CC101				1													1
	CC121	1		2	8		3	2	6			2		1	1	60		86
	CC177										2				19		1	22
	CC199								3									3
	CC200			1														1
	CC403															27		27
	CC415	3	1		10					1						15		30
Grand total		148	14	18	192	4	34	10	24	11	25	68	2	3	20	188	8	769

Table S3: Prevalence and distribution of plasmids in each CC

	Frequency (%) of plasmids in *L. monocytogenes* isolates (n = total no of strains)						
	This study – food processing industry					Clinical isolates [60, 61]	rural/urban/ farm/slug environments [61]
	repA G1	*repA* G2	*repA* G1 and G2	*repA* G12	in total		
**Lineage I total**	29 %	2 %	10 %	0 %	41% (n=97)	25% (n=24)	0% (n=25)
**CC1**					0% (n=34)	17% (n=6)**	0% (n=8)
**CC2**					0% (n=7)		
**CC3**	100 %				100% (n=24)	100% (n=1)	
**CC4**					0% (n=1)	0% (n=2)	0% (n=9)
**CC5**	23 %		77 %		100% (n=13)	100% (n=2)	
**CC6**	33 %				33% (n=3)	0% (n=2)	0% (n=6)
**CC59**						0% (n=1)	
**CC87**						0% (n=6)	
**CC88**		100 %			100% (n=2)	100% (n=2)	
**CC220**					0% (n=2)	0% (n=2)	0% (n=1)
**CC224**							0% (n=1)
**CC315**					0% (n=10)		
**Lineage II total**	40 %	20 %		0.1 %	60% (n=672)	26% (n=87)*	9% (n=193)
**CC7**	9 %	19 %		1 %	29% (n=68)	11% (n=27)*	47% (n=17)
**CC8**		95 %			95% (n=19)	43% (n=7)	63% (n=8)
**CC9**	83 %				83% (n=290)	50% (n=2)	100% (n=1)
**CC11**		11 %			11% (n=9)	0% (n=2)	4% (n=26)
**CC14**					0% (n=20)	0% (n=3)	0% (n=6)
**CC18**					0% (n=5)	0% (n=5)	0% (n=16)
**CC19**					0% (n=55)	0% (n=5)	0% (n=17)
**CC20**		25 %			25% (n=4)	0% (n=5)	8% (n=12)
**CC21**		50 %			50% (n=2)		0% (n=3)
**CC29**							0% (n=2)
**CC31**	100 %				100% (n=3)	0% (n=1)	0% (n=1)
**CC37**					0% (n=14)	0% (n=3)	0% (n=22)
**CC89**						0% (n=1)	
**CC90**							0% (n=5)
**CC91**					0% (n=14)	0% (n=1)	0% (n=32)
**CC101**	100 %				100% (n=1)	0% (n=2)	
**CC121**		100 %			100% (n=86)	100% (n=15)	0% (n=1)
**CC124**							0% (n=1)
**CC177**					0% (n=22)	0% (n=2)	0% (n=4)
**CC199**	100 %				100% (n=3)	100% (n=1)	
**CC200**					0% (n=1)		
**CC204**							0% (n=5)
**CC226**						0% (n=2)	0% (n=3)
**CC403**					0% (n=27)	0% (n=1)	0% (n=2)
**CC412**							0% (n=2)
**CC415**	50 %	50 %			100% (n=30)	0% (n=2)	20% (n=5)
**CC475**							0% (n=1)
**CC671**							0% (n=1)
**Grand total**	39 %	18 %	1 %	0.1 %	58% (n=769)	26% (n=111)*	8% (n=218)

* Plasmid count includes 2 small non-*repA* plasmids
** One of these was the *repA* G4 plasmid

Table S4: BLAST analysis of stress survival and resistance genes

BLAST results			Gene(s) used as queries in BLAST							
Core gene	Acces-sory gene*	Absent in all genomes	Gene	GenBank accession no.	Locus tag/region used as query	Assigned operon	Source *Lm* strain or sequence	Stress condition	Function	Ref.
X			*arcA*	NC_003210.1	lmo0043		EGD-e	acid stress	arginin deiminase	[62]
X			*arcC*	NC_003210.1	lmo0039		EGD-e	acid stress	carbamate kinase	[62]
X			*argB*	NC_003210.1	lmo1589	*argCJBDF* operon	EGD-e	acid stress	acetylglutamate kinase	[62]
X			*argC*	NC_003210.1	lmo1591	*argCJBDF* operon	EGD-e	acid stress	N-acetyl-gamma-glutamyl-phosphate reductase	[62]
X			*argD*	NC_003210.1	lmo1588	*argCJBDF* operon	EGD-e	acid stress	acetylornithine aminotransferase	[62]
X			*argF*	NC_003210.1	lmo1587	*argCJBDF* operon	EGD-e	acid stress	ornithine carbamoyltransferase	[62]
X			*argG*	NC_003210.1	lmo2090	*argGH* operon	EGD-e	acid stress	argininosuccinate synthase	[62]
X			*argH*	NC_003210.1	lmo2091	*argGH* operon	EGD-e	acid stress	argininosuccinate lyase	[62]
X			*argJ*	NC_003210.1	lmo1590	*argCJBDF* operon	EGD-e	acid stress	acetyltransferase	[62]
X			*argR*	NC_003210.1	lmo1367		EGD-e	acid stress	transcriptional regulator of the arginine deiminase system	[62]
	X		*arsA1D1R1D2 R2A2B1B2*	CM001159.1	LMOSA_2210-2280 (from genomic island LGI2)	*arsA1D1R1D2R2A2B 1B2*	Scott A	arsenic	arsenic resistance genomic island	[9]
	X		*arsCBADR*	MZ090008	pLIS28_00021		70Lodz, plasmid pLIS28, Tn*554*-like transposon	arsenic	arsenic resistance transposon	[8]
	X		*bapL*	NC_003210.1	lmo0435		EGD	biofilm formation	biofilm-associated protein	[34]
	X		*bcrABC*	CP025260.1	CV733_15740, CV733_15735, and CV733_15730	*bcrABC*	MF4624	benzalkonium chloride	benzalkonium chloride resistance	[18, 19]
X			*betL*	NC_003210.1	lmo2092		EGD-e	osmotic stress	glycine betaine transporter	[63]
	X		*cadA1C1*	CP023753.1	CRD58_15970, CRD58_15975	*cadA1C1*	AT3E, plasmid pLM58	cadmium	cadmium resistance cassette located on transposon Tn*5422*	[10, 64]
	X		*cadA2C2*	AADR01000058.1	LMOh7858_pLM80_0083, LMOh7858_pLM80_0082	*cadA2C2*	H7858, plasmid pLM80	cadmium	cadmium resistance cassette located on plasmid pLM80	[10, 64]
		X	*cadA3C3*	NC_003210.1	lmo1100, lmo1102	*cadA3C3*	EGD-e	cadmium	cadmium resistance cassette located in an integrative conjugative element	[10, 64]
	X		*cadA4C4*	CM001159.1	LMOSA_2330, LMOSA_2321	*cadA4C4*	Scott A	cadmium	cadmium resistance cassette	[10]
	X		*cadA5C5*	NZ_MIMA010000 12	BG835_RS07990, BG835_RS07995	*cadA5C5*	OLM 10	cadmium	cadmium resistance cassette	[7, 9]
		X	*cadA6aC6a*	MW124301	pLIS400101c, pLIS400106c	*cadA6aC6a*	*L. seeligeri* Sr12 plasmid pLIS4	cadmium	cadmium resistance cassette	[11]
		X	*cadA6bC6b*	MW124302	pLIS600081, pLIS600076	*cadA6bC6b*	*L. ivanovii* strain Sr11 plasmid pLIS6	cadmium	cadmium resistance cassette	[11]
	X		*clpL*	CP023753.1	CRD58_16010		AT3E, plasmid pLM58	heat stress	ATP-dependent protease	[65]

X			*clpP*	NC_003210.1	lmo2468		EGD-e	heat stress	ATP-dependent Clp protease proteolytic subunit	[66]
X			*clpX*	NC_003210.1	lmo1268		EGD-e	heat stress	chaperone for ClpP	[67]
X			*cspD*	NC_003210.1	lmo1879		EGD-e	cold and osmotic stress	cold shock protein	[68]
X			*ctsR*	NC_003210.1	lmo0229		EGD-e	heat stress	transcriptional regulator	[66]
X			*dnaJ*	NC_003210.1	lmo1472	*dnaK* operon	EGD-e	heat stress	heat shock protein	[69]
X			*dnaK*	NC_003210.1	lmo1473	*dnaK* operon	EGD-e	heat stress	heat shock protein	[69]
	C		*emrC*	CP038643	E5D16_15160		N12-0935	QAC resistance	multidrug efflux SMR transporter	[6, 15]
		X	*emrE*	LJOZ02000007.1	AOA13_1592c		198	quaternary ammonium compounds	quaternary ammonium compounds efflux pump	[16, 17]
X			*fepA*	NC_003210.1	lmo2087		EGD-e	fluoroquinolone resistance and contribution to QAC adaptation	MATE-family efflux pump, FepA fluoroquinolone efflux pump (and regulator)	[29]
X			*fepR*	NC_003210.1	lmo2088		EGD-e	fluoroquinolone resistance and contribution to QAC adaptation	MATE-family efflux pump, FepA fluoroquinolone efflux pump (and regulator)	[29]
X			*fosX_2*		lmo1702; ResFinder database			Fosfomycin resistance	Catalyzes the hydration of fosfomycin	[70, 71]
X			*fur*	NC_003210.1	lmo1956		EGD-e	heme stress	transcriptional regulator	[72]
X			*gadC*	NC_003210.1	lmo2362		EGD-e	acid stress	amino acid antiporter	[73]
	X		*gadD1*	NC_003210.1	lmo0447	SSI-1	EGD-e	acid stress	glutamate decarboxylase acid resistance system, SSI-1	[38, 74]
X			*gadD2*	NC_003210.1	lmo2363		EGD-e	acid stress	glutamate decarboxylase (GAD) system responsible for acid resistance	[74]
X			*gadD3*	NC_003210.1	lmo2434		EGD-e	acid stress	glutamate decarboxylase (GAD) system responsible for acid resistance	[74]
X			*gbuA*	NC_003210.1	lmo1014	*gbuABC* operon	EGD-e	osmotic stress	glycine betaine/proline transport system ATP-binding protein	[75, 76]
X			*gbuB*	NC_003210.1	lmo1015	*gbuABC* operon	EGD-e	osmotic stress	glycine betaine/proline transport system permease	[75, 76]
X			*gbuC*	NC_003210.1	lmo1016	*gbuABC* operon	EGD-e	osmotic stress	glycine betaine-binding protein	[75, 76]
	X		*gbuC*-like gene	HG813248.1	LMR479A_p0089		R479a, plasmid R479a	salt stress	putative glycine-betaine transporter binding protein	[77]
X			*groEL*	NC_003210.1	lmo2068	*groESL* operon	EGD-e	heat stress	molecular chaperone	[78]
X			*groES*	NC_003210.1	lmo2069	*groESL* operon	EGD-e	heat stress	molecular chaperone	[78]
X			*grpE*	NC_003210.1	lmo1474	*dnaK* operon	EGD-e	heat stress	heat shock protein	[69]
X			*hrcA*	NC_003210.1	lmo1475	*dnaK* operon	EGD-e	heat stress	heat-inducible transcription repressor	[69]
X			*hsdM*	NC_003210.1	lmo1582		EGD-e	osmotic stress	DNA methylase	[79]
	X		*inlA* PMSC mutation	NC_003210.1	lmo0433	*inlAB*	EGD-e	Virulence, adhesion, dessication stress	internalin A	[4, 42, 47]

	X		*inlB PMSC mutation*	NC_003210.1	lmo0434	*inlAB*	EGD-e	Virulence, biofilm formation	internalin B	[42, 47]
	X		*inlL*	NC_003210.1	lmo2026			Adhesion and biofilm formation	biofilm-associated protein	[35]
X			*kat*	NC_003210.1	lmo2785		EGD-e	oxidative stress	catalase	[80]
X			*lde*		lmo2741		EGD-e	*Listeria* drug efflux	fluoroquinolone resistance	[27, 28]
X			*ldh*	NC_003210.1	lmo0210		EGD-e	energy stress and cellular metabolism	L-lactate dehydrogenase	[80]
	X		*lin0465*	HG813249.1	LM6179_0749	SSI-2	6179	alkaline and oxidative stress	putative PfpI protease, SSI-2	[39]
X			*lisK*	NC_003210.1	lmo1378	*lisRK* operon	EGD-e	general stress response	two-component sensor histidine kinase	[81]
X			*lisR*	NC_003210.1	lmo1377	*lisRK* operon	EGD-e	general stress response	two-component response regulator	[81]
X			*lmo0398*	NC_003210.1	lmo0398		EGD-e	oxidative stress	PTS sugar transporter subunit IIA	[80]
X			*lmo0399*	NC_003210.1	lmo0399		EGD-e	oxidative stress	PTS sugar transporter subunit IIB	[80]
X			*lmo0400*	NC_003210.1	lmo0400		EGD-e	oxidative stress	PTS sugar transporter subunit IIC	[80]
X			*lmo0669*	NC_003210.1	lmo0669		EGD-e	oxidative stress	oxidoreductase	[82]
X			*lmo0781*	NC_003210.1	lmo0781		EGD-e	oxidative stress	PTS mannose transporter subunit IID	[80]
X			*lmo0782*	NC_003210.1	lmo0782		EGD-e	oxidative stress	PTS mannose transporter subunit IIC	[80]
X			*lmo0783*	NC_003210.1	lmo0783		EGD-e	oxidative stress	PTS mannose transporter subunit IIB	[80]
X			*lmo0784*	NC_003210.1	lmo0784		EGD-e	oxidative stress	PTS mannose transporter subunit IIB	[80]
X			*lmo0799*	NC_003210.1	lmo0799		EGD-e	blue light	blue light photoreceptor	[83]
X			*lmo1433*	NC_003210.1	lmo1433		EGD-e	oxidative stress	glutathione reductase	[84]
X			*lmo1470*	NC_003210.1	lmo1470	*dnaK* operon	EGD-e	general stress response	16S ribosomal RNA methyltransferase RsmE	[85]
X			*lmo1601*	NC_003210.1	lmo1601		EGD-e	general stress response	general stress protein	[80]
X			*lmo1602*	NC_003210.1	lmo1602		EGD-e	general stress response	hypothetical protein encoded in the same operon as *lmo1601*	[80]
X			*lmo2230*	NC_003210.1	lmo2230		EGD-e	arsenate	arsenate reductase	[86]
	N		*LMOf2365_0481*	AE017262.2	LMOf2365_0481		F2365	None detected	hypothetical protein of unknown function encoded in the same hypervariable region as SSI-1 and SSI-2	[87]
X			*ltrC*	NC_003210.1	lmo2398		EGD-e	cold stress	C protein required at low temperatures	[88]
	X		*mco*	HG813248.1	LMR479A_p0066		R479a, plasmid R479a	copper, acid stress	multicopper oxidase	[77, 89, 90]
X			*mdrL*	NC_003210.1	lmo1409		LO28	benzalkonium chloride	multidrug efflux MFS transporter	[24-26]
X			*mepA*	CYWG01000004.1	LM800396_120197		LM08-00396	QAC resistance	MATE-family efflux pump	[30]
	X		NiCo riboswitch	HG813248.1			R479a, plasmid R479a	heavy metal, acid stress	uncharacterized NiCo riboswitch	[90]

	X		*npr*	HG813248.1	LMR479A_p0090		R479a, plasmid R479a	oxidative, salt, acid stress	putative NADH peroxidase	[77]
X			*oppA*	NC_003210.1	lmo2569		EGD-e	cold stress	peptide ABC transporter substrate-binding protein	[91]
X			*opuCA*	NC_003210.1	lmo1428	*opuC* operon	EGD-e	osmotic stress	osmoprotectant transport system ATP-binding protein	[92]
X			*opuCB*	NC_003210.1	lmo1427	*opuC* operon	EGD-e	osmotic stress	osmoprotectant transport system permease	[92]
X			*opuCC*	NC_003210.1	lmo1426	*opuC* operon	EGD-e	osmotic stress	osmoprotectant transport system substrate-binding protein	[92]
X			*opuCD*	NC_003210.1	lmo1425	*opuC* operon	EGD-e	osmotic stress	osmoprotectant transport system permease	[92]
X			*pdhB*	NC_003210.1	lmo1053		EGD-e	oxidative stress	pyruvate dehydrogenase E1 component subunit beta	[80]
X			*perR*	NC_003210.1	lmo1683		EGD-e	oxidative stress	peroxide operon transcriptional regulator	[93]
	X		*qacH* (Tn*6188*)	MK944277.1			16-LI00532-0	QAC resistance	quaternary ammonium compounds resistance	[13]
X			*rli47*	NC_003210.1	lmos81		EGD-e	lactic acid stress	sigB-dependent ncRNA	[90]
X			*rsbR*	NC_003210.1	lmo0889	*sigB* operon	EGD-e	stressosome	positive regulator of sigB activity	[94]
X			*rsbS*	NC_003210.1	lmo0890	*sigB* operon	EGD-e	stressosome	negative regulator of sigB activity	[94]
X			*rsbT*	NC_003210.1	lmo0891	*sigB* operon	EGD-e	stressosome	positive regulator of sigB activity	[95]
X			*rsbU*	NC_003210.1	lmo0892	*sigB* operon	EGD-e	stressosome	serine phosphatase	[94]
X			*rsbV*	NC_003210.1	lmo0893	*sigB* operon	EGD-e	stressosome	anti-anti-sigma factor (antagonist of RsbW)	[95]
X			*rsbW*	NC_003210.1	lmo0894	*sigB* operon	EGD-e	stressosome	serine-protein kinase	[95]
X			*rsbX*	NC_003210.1	lmo0896	*sigB* operon	EGD-e	stressosome	phosphoserine phosphatase	[95]
X			*sigB*	NC_003210.1	lmo0895	*sigB* operon	EGD-e	general stress response	RNA polymerase sigma factor	[96]
X			*sigL*	NC_003210.1	lmo2461		EGD-e	cold stress	RNA polymerase sigma-54 factor	[97]
X			*sugE1*	NC_003210.1	lmo0853	*sug* operon	EGD-e	QAC resistance	SMR transporter	[23]
X			*sugE2*	NC_003210.1	lmo0854	*sug* operon	EGD-e	QAC resistance	SMR transporter	[23]
X			*sugR*	NC_003210.1	lmo0852	*sug* operon	EGD-e	QAC resistance	SMR transporter	(23]
	C		*tet*(M)	FN433596	*tet*(M) allele 7; ResFinder database		Staphylococcus aureus subsp. aureus TW20	tetracycline resistance	tetracycline resistance ribosomal protection protein	[70, 98]
X			*tetR*	KJ000253.1	1152...1745		BM4715	antibiotics	transcriptional repressor in the regulation of several genes for drug efflux systems	[99, 100]
	X		*tmr*	AADR01000010.1	LMOh7858_pLM80_0067		H7858, plasmid pLM80	dye detoxification	triphenylmethane reductase	[22]
X			*uspA*	NC_003210.1	lmo0515		EGD-e	general stress response	universal stress protein A	[101]

* **X** denotes accessory stress survival gene or resistance gene identified among the food processing environment isolates. **C** indicates a gene only found in one clinical isolate. **N** indicates that this gene does not have an identified stress-associated function (it is encoded in the same hypervariable region as SSI-1 and SSI-2).

Table S5: Pearson's chi-square test: source meat/salmon

Results from statistical test for association between source of isolates (meat processing [hygienic and low-hygienic zones] and salmon processing environments) and the presence or absence of categories of genetic determinants of stress survival and resistance.

Test for association between:		Including CC9			Excluding CC9		
**Source of isolates**	**Presence vs. absence of genetic determinants**	**χ^2 test statistic**	**DF**	***p*-value**	**χ^2 test statistic**	**DF**	***p*-value**
Meat processing; hygienic zone vs. salmon processing	plasmid(s)	59.685	1	<0.001	0.602	1	0.438
	cadmium resistance genes	7.270	1	0.007	10.756	1	0.001
	arsenic resistance genes	122.322	1	<0.001	39.571	1	<0.001
	QAC resistance genes	129.831	1	<0.001	3.010	1	0.083
	biofilm-associated genes	144.400	1	<0.001	10.148	1	0.001
	SSI-1 or SSI-2	76.235	1	<0.001	0.002	1	0.966
	inlA PMSC mutation	179.883	1	<0.001	0.379	1	0.538
Meat processing; **low** hygienic zone vs. salmon processing	plasmid(s)	0.804	1	0.370	1.819	1	0.177
	cadmium resistance genes	2.536	1	0.111	3.362	1	0.067
	arsenic resistance genes	0.102	1	0.750*	0.982	1	0.322*
	QAC resistance genes	0	1	0.983*	0.652	1	0.420*
	biofilm-associated genes	0.059	1	0.809	1.157	1	0.282
	SSI-1 or SSI-2	1.208	1	0.272	3.466	1	0.063
	inlA PMSC mutation	0.210	1	0.647*	0.787	1	0.375*
Meat processing; hygienic zone vs. Meat processing; **low** hygienic zone	plasmid(s)	12.696	1	<0.001	2.550	1	0.110
	cadmium resistance genes	6.648	1	0.010	0.290	1	0.590
	arsenic resistance genes	11.151	1	0.001	11.825	1	**
	QAC resistance genes	13.536	1	<0.001	0.034	1	0.853
	biofilm-associated genes	26.787	1	<0.001*	5.141	1	0.023*
	SSI-1 or SSI-2	22.170	1	<0.001*	3.237	1	0.072
	inlA PMSC mutation	17.758	1	<0.001	0.448	1	0.503

*1 cell(s) with expected counts less than 5.
** Chi-square approximation probably invalid. 2 cell(s) with expected counts less than 5.

Table S6: Pearson's chi-square test: persistence and pervasion

Results from statistical test for association between isolates designated as persistent *vs.* non-persistent and isolates designated as pervasive *vs.* non-pervasive and the presence or absence of categories of genetic determinants of stress survival and resistance.

**Test for association between:**		**χ^2 test statistic**	**DF**	***p*-value**
**Category**	**Presence vs. absence of genetic determinants**			
Persistent vs. non-persistent	plasmid(s)	0.090	1	0.764
	cadmium resistance genes	0.597	1	0.440
	arsenic resistance genes	2.108	1	0.147
	QAC resistance genes	5.338	1	0.021
	biofilm-associated genes	4.059	1	0.044
	SSI-1 or SSI-2	0.267	1	0.605
	inlA PMSC mutation	2.704	1	0.100
Pervasive vs. non-pervasive	plasmid(s)	27.22	1	<0.001
	cadmium resistance genes	57.883	1	<0.001
	arsenic resistance genes	150.845	1	<0.001
	QAC resistance genes	265.923	1	<0.001
	biofilm-associated genes	23.151	1	<0.001
	SSI-1 or SSI-2	58.088	1	<0.001
	inlA PMSC mutation	159.926	1	<0.001
	clpL	59,322	1	<0.001
	cadA1C1	27,673	1	<0.001
	cadA2C2	8,197	1	0.004
	bcrABC	50,822	1	<0.001
	*qacH*_Tn*6188*	146,217	1	<0.001
	LGI2_ars_operon	12,834	1	<0.001
	Tn554_arsCBADR	146,827	2	<0.001
	gadD1_SSI_1	25,137	1	<0.001
	lin0465_SSI_2	9,808	1	0.002
	*bapL**	126,464	1	<0.001
	*inlL***	24,710	1	<0.001

* BapL was designated as 'absent' in isolates with PMSC mutation at codon 61 (full-length BapL is 2013 aa in length)

** Isolates encoding InlL of both length 626 aa and length 621 aa were designated as 'present'.

Table S7: Pearson's chi-square test: source food/clinical/nature

Results from statistical test for association between source of isolates (food processing environment, natural environment, clinical isolates) and the presence or absence of categories of genetic determinants of stress survival and resistance.

**Test for association between:**		**χ^2 test statistic**	**DF**	***p*-value**
**Source of isolates**	**Presence vs. absence of genetic determinants**			
Food processing environments vs. clinical isolates	plasmid(s)	41.575	1	<0.001
	cadmium resistance genes	62.613	1	<0.001
	arsenic resistance genes	71.533	1	<0.001
	QAC resistance genes	33.392	1	<0.001
	biofilm-associated genes	11.739	1	0.001
	SSI-1 or SSI-2	8.840	1	0.003
	inlA PMSC mutation	48.555	1	<0.001
Food processing environments vs. natural environments	plasmid(s)	171.017	1	<0.001
	cadmium resistance genes	182.401	1	<0.001
	arsenic resistance genes	101.63	1	<0.001
	QAC resistance genes	151.704	1	<0.001
	biofilm-associated genes	14.898	1	<0.001
	SSI-1 or SSI-2	159.261	1	<0.001
	inlA PMSC mutation	182.01	1	<0.001
Clinical isolates vs. natural environments	plasmid(s)	18.919	1	<0.001
	cadmium resistance genes	7.641	1	0.006
	arsenic resistance genes	2.284	1	0.131
	QAC resistance genes	37.397	1	<0.001
	biofilm-associated genes	0.161	1	0.688
	SSI-1 or SSI-2	35.689	1	<0.001
	inlA PMSC mutation	35.207	1	<0.001

Table S8: Plasmids used in BLAST analysis

Plasmids were selected from the study by Chmielowska et al. [2].

GenBank accession no.	Size (bp)	Strain	Plasmid name	Species	Replication system group
CM008329.1	58 524	198	p198	*L. monocytogenes*	*repA* G1
MT459813.1	52 825	2017-TE-6913-1	p2017-TE-6913-1	*L. monocytogenes*	*repA* G1
CP045971.1	2 776	AUSMDU00000235	pAUSMDU00000235	*L. monocytogenes*	?
CP045750.1	51 115	CFSAN008100	pCFSAN008100 (pCFSAN008100a)	*L. monocytogenes*	*repA* G1
CP011399.1	68 224	CFSAN008100	pCFSAN008100 (pCFSAN008100b)	*L. monocytogenes*	*repA* G1
CP014251.1	55 521	CFSAN010068	pCFSAN010068_01	*L. monocytogenes*	*repA* G1
CP022021.1	152 337	FDA00006907	pCFSAN021445	*L. monocytogenes*	*repA* G1 + *repA* G2
CP020829.1	67 033	CFSAN022990	pCFSAN022990	*L. monocytogenes*	*repA* G2
CP023051.1	61 922	FDA00011238	pCFSAN059932	*L. monocytogenes*	*repA* G1
KC456362.1	7 641	TTS-2011	pDB2011	*Listeria innocua*	RCR family Rep
CP028184.1	62 214	CFSAN054109	pGMI16-004	*L. monocytogenes*	*repA* G2
U40997.1	3 712	BM4293	pIP823	*L. monocytogenes*	RCR family Rep
CP062128.1	77 825	FSL J1-208	pj1-0208 = pLMIV	*L. monocytogenes*	*repA* G3
FR667693.1	79 249	DSM20601	pLGUG1	*Listeria grayi*	*repA* G2
AL592102.1	81 905	Clip11262	pLI100	*Listeria innocua*	*repA* G2
MZ043158.1	60 411	53/04	pLIS10	*L. monocytogenes*	*repA* G1
MZ065170.1	65 608	112Lodz	pLIS11	*L. monocytogenes*	*repA* G2
MZ043154.1	70 920	434/05	pLIS14	*L. monocytogenes*	*repA* G2
MZ089996.1	29 940	Lmo28	pLIS16	*L. monocytogenes*	*repA* G2
MZ089997.1	29 506	43/06	pLIS17	*L. monocytogenes*	*repA* G1
MW934262.1	91 964	24/04	pLIS2	*Listeria innocua*	*repA* G1
MZ090000.1	31 739	11/07	pLIS20	*L. monocytogenes*	*repA* G1
MZ090001.1	60 921	05/09	pLIS21	*L. monocytogenes*	*repA* G2
MZ090004.1	46 284	06/09	pLIS24	*L. monocytogenes*	*repA* G1
MZ090005.1	77 818	09/09/S	pLIS25	*L. monocytogenes*	*repA* G2
MZ090006.1	54 219	417/10/SW	pLIS26	*L. monocytogenes*	*repA* G4
MZ090007.1	48 877	417/10/SW	pLIS27	*L. monocytogenes*	*repA* G1
MZ090008.1	66 985	70 łodź	pLIS28	*L. monocytogenes*	*repA* G1
MZ090010.1	28 400	58/10/SW	pLIS30	*L. monocytogenes*	*repA* G1
MZ127840.1	31 265	1027 PZH	pLIS31	*L. monocytogenes*	*repA* G1
MZ127841.1	70 718	30/10/S	pLIS32	*L. monocytogenes*	*repA* G2
MZ127843.1	63 146	11PZH	pLIS34	*L. monocytogenes*	*repA* G1
MZ127844.1	69 726	62/06	pLIS35	*Listeria innocua*	*repA* G1
MZ127845.1	55 161	165/05	pLIS36	*Listeria innocua*	*repA* G1
MZ127846.1	91 920	65/06	pLIS37	*Listeria innocua*	*repA* G1
MW124301.1	101 192	Sr12	pLIS4	*Listeria seeligeri*	*repA* G6
MZ127849.1	63 046	04/07	pLIS40	*Listeria innocua*	*repA* G2
MZ147614.1	79 330	114/05	pLIS41	*Listeria innocua*	*repA* G2
MZ230002.1	51 747	Sr102	pLIS42	*Listeria innocua*	*repA* G4
MZ147615.1	72 838	Sr108	pLIS43	*Listeria innocua*	*repA* G1
MZ147616.1	60 559	Sr114	pLIS44	*Listeria innocua*	*repA* G4
MZ147618.1	50 430	Sr19	pLIS47	*Listeria ivanovii*	*repA* G5
MZ147619.1	73 914	Sr39	pLIS48	*Listeria ivanovii*	*repA* G5
MZ147620.1	47 009	45/06	pLIS49	*Listeria welshimeri*	*repA* G1
CP063072.1	60 659	Sr73	pLIS5	*Listeria seeligeri*	*repA* G5
MZ151536.1	83 141	FSL S4 0057	pLIS51	*Listeria seeligeri*	*repA* G6
MZ151537.1	46 586	Sr77	pLIS52	*Listeria seeligeri*	*repA* G5
MZ151538.1	72 088	19/09/S	pLIS54	*Listeria grayi*	*repA* G1
MZ151539.1	9 622	09/09/S	pLIS55	*L. monocytogenes*	?
MW124302.1	59 142	Sr11	pLIS6	*Listeria ivanovii*	*repA* G5
MW927705.1	30 460	53/06	pLIS7	*L. monocytogenes*	*repA* G1
MZ043155.1	91 343	6E/09	pLIS8	*L. monocytogenes*	*repA* G2
MZ043156.1	81 274	38/04	pLIS9	*L. monocytogenes*	*repA* G1
FR667692.1	57 780	SLCC2755	pLM1-2bUG1	*L. monocytogenes*	*repA* G1
MK134858.1	91 245	-	pLM1686	*L. monocytogenes*	*repA* G2
GU244485.1	32 307	Lm1	pLM33	*L. monocytogenes*	*repA* G1
CP019166.1	77 105	HPB5415	pLM5578	*L. monocytogenes*	*repA* G2
CM009923.1	81 666	LM-F-131	pLM-F-131	*L. monocytogenes*	*repA* G2
CP038643.1	4 392	N12-0935	pLmN12-0935	*L. monocytogenes*	RCR family Rep
HG813248.1	86 652	R479a	pLMR479a	*L. monocytogenes*	*repA* G2

CP025444.1	25 550	MF4545	pMF4545	*L. monocytogenes*	*repA* G1
CP025260.1	63 182	MF4624	pMF4624	*L. monocytogenes*	*repA* G1
CP025083.1	58 524	MF4626	pMF4626	*L. monocytogenes*	*repA* G1
CP025566.1	15 792	ATCC 51779	pPIR00541	*L. monocytogenes*	*repA* G1
CP062125.1	50 101	FSL R9-0915	pr9-0915	*L. monocytogenes*	*repA* G1
CP015509.1	33 502	F4244	unnamed (pF4244)	*L. monocytogenes*	*repA* G1
CP006612.1	55 804	J1776	unnamed (pJ1776)	*L. monocytogenes*	*repA* G1
CP006611.1	148 959	N1-011A	unnamed (pN1-011A)	*L. monocytogenes*	*repA* G1 + *repA* G2
MH277333.1	85 554	NH1	unnamed (pNH1)	*L. monocytogenes*	*repA* G1

Table S9: Genes encoding RepA used in BLAST analysis

Gene name	Protein ID	GenBank accession no (DNA sequence)	locus_tag	Strain	Plasmid name	Species
repA (G1)	AUH56704.1	CP025444.1	CV731_15405	MF4545	pMF4545	*L. monocytogenes*
repA (G2)	APV05983.1	CP019166.1	BWI19_15595	HPB5415	pLM5578	*L. monocytogenes*
repA (G3)	QOF63826.1	CP062128.1	IFI77_14350	FSL J1-208	pj1-0208 = pLMIV	*L. monocytogenes*
repA (G4)	UCK60685.1	MZ090006.1	pLIS26_00001c	417/10/SW	pLIS26	*L. monocytogenes*
repA (G5)	QPL19438.1	MW124302.1	pLIS600001c	Sr11	pLIS6	*Listeria ivanovii*
repA (G6)	QPL19350.1	MW124301.1	pLIS400001c	Sr12	pLIS4	*Listeria seeligeri*
repA (G7)	WP_187136074.1	NZ_JAARUF010000013.1	HB999_RS14515	FSL L7-1071	(draft genome)	*Listeria booriae*
repA (G8)	EAC9277011.1	AAAKTE010000016.1	B1N12_15035	CFSAN060100	(draft genome)	*L. monocytogenes*
repA (G9)	WP_031644646.1	NZ_PQHI01000013.1	CXR94_RS14695	CDPHFDLB-FM17-00092	(draft genome)	*L. monocytogenes*
repA (G10)	HAB8811037.1	DAAIMV010000022.1	GYS95_12350	CFIAFB20120154	(draft genome)	*L. monocytogenes*
repA (G11)	WP_061665668.1	NZ_LUEQ01000011.1	AWI79_RS14870	CFSAN026587	(draft genome)	*L. monocytogenes*

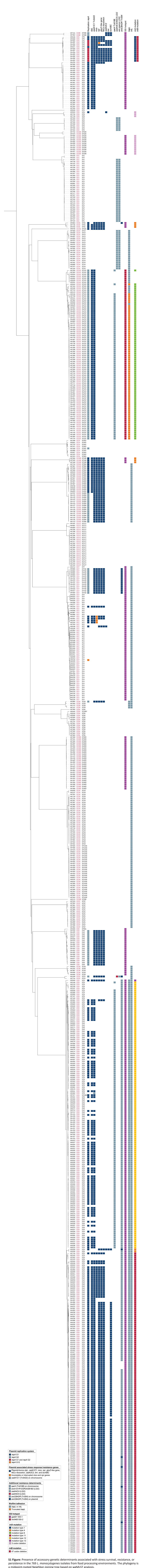

S1 Figure: Presence of accessory genetic determinants associated with stress survival, resistance, or persistence in the 769 *L. monocytogenes* isolates from food processing environments. The phylogeny is a midpoint-rooted Neighbor-Joining tree based on wgMLST analysis.

Figure S2: Presence of *bcrABC* and *qacH* in CC9

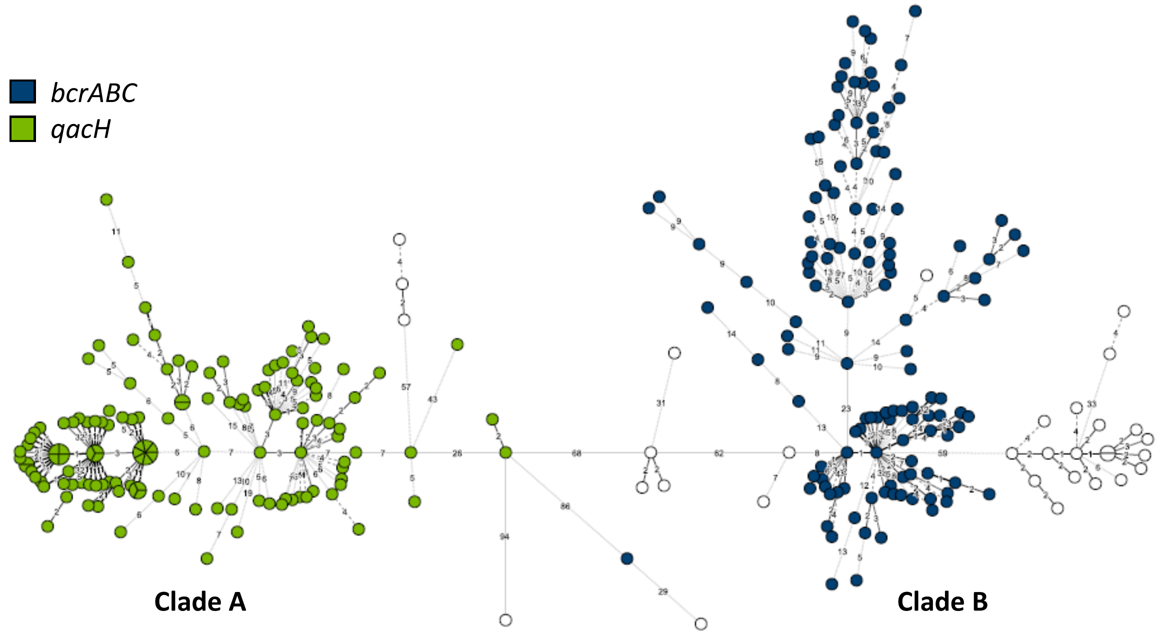

Figure S2: Distribution of *bcrABC* and *qacH* QAC resistance genes in the CC9 isolates (n=290). A minimum spanning tree where nodes are colored by the presence of *bcrABC* and *qacH*. Labelling of Clades A and B is according to Fagerlund *et al*. [21]. The area of each node is proportional to the number of isolates represented, and the number of allelic differences between isolates is indicated on the branches connecting two nodes. Branch lengths are square root scaled.

References

1. Kuenne C, Voget S, Pischimarov J, Oehm S, Goesmann A, Daniel R, Hain T, Chakraborty T. 2010. Comparative analysis of plasmids in the genus *Listeria*. *PLoS One* **5:**e12511.
2. Chmielowska C, Korsak D, Chapkauskaitse E, Decewicz P, Lasek R, Szuplewska M, Bartosik D. 2021. Plasmidome of *Listeria* spp. – The *repA*-family business. *Int J Mol Sci* **22:**10320.
3. Fagerlund A, Langsrud S, Moen B, Heir E, Møretrø T. 2018. Complete genome sequences of six *Listeria monocytogenes* sequence type 9 isolates from meat processing plants in Norway. *Genome Announc* **6:**e00016-18.
4. Hingston P, Chen J, Dhillon BK, Laing C, Bertelli C, Gannon V, Tasara T, Allen K, Brinkman FS, Truelstrup Hansen L, Wang S. 2017. Genotypes associated with *Listeria monocytogenes* isolates displaying impaired or enhanced tolerances to cold, salt, acid, or desiccation stress. *Front Microbiol* **8:**369.
5. Baines SL, da Silva AG, Carter GP, Jennison A, Rathnayake I, Graham RM, Sintchenko V, Wang Q, Rockett RJ, Timms VJ, Martinez E, Ballard S, Tomita T, Isles N, Horan KA, Pitchers W, Stinear TP, Williamson DA, Howden BP, Seemann T, Communicable Diseases Genomics Network (CDGN). 2020. Complete microbial genomes for public health in Australia and the Southwest Pacific. *Microb Genom* **6:**000471.
6. Kropac AC, Eshwar AK, Stephan R, Tasara T. 2019. New insights on the role of the pLMST6 plasmid in *Listeria monocytogenes* biocide tolerance and virulence. *Front Microbiol* **10:**1538.
7. Parsons C, Lee S, Kathariou S. 2020. Dissemination and conservation of cadmium and arsenic resistance determinants in *Listeria* and other Gram-positive bacteria. *Mol Microbiol* **113:**560-569.
8. Kuenne C, Billion A, Mraheil MA, Strittmatter A, Daniel R, Goesmann A, Barbuddhe S, Hain T, Chakraborty T. 2013. Reassessment of the *Listeria monocytogenes* pan-genome reveals dynamic integration hotspots and mobile genetic elements as major components of the accessory genome. *BMC Genom* **14:**47.
9. Lee S, Ward TJ, Jima DD, Parsons C, Kathariou S. 2017. The arsenic resistance-associated *Listeria* Genomic Island LGI2 exhibits sequence and integration site diversity and a propensity for three *Listeria monocytogenes* clones with enhanced virulence. *Appl Environ Microbiol* **83:**e01189-17.
10. Parsons C, Lee S, Jayeola V, Kathariou S. 2017. Novel cadmium resistance determinant in *Listeria monocytogenes*. *Appl Environ Microbiol* **83:**e02580-16.
11. Chmielowska C, Korsak D, Szmulkowska B, Krop A, Lipka K, Krupińska M, Bartosik D. 2020. Genetic carriers and genomic distribution of *cadA6* - A novel variant of a cadmium resistance determinant identified in *Listeria* spp. *Int J Mol Sci* **21:**8713.
12. Lee S, Parsons C, Chen Y, Hanafy Z, Brown E, Kathariou S. 2021. Identification and characterization of a novel genomic island harboring cadmium and arsenic resistance genes in *Listeria welshimeri*. *Biomolecules* **11:**560.
13. Müller A, Rychli K, Muhterem-Uyar M, Zaiser A, Stessl B, Guinane CM, Cotter PD, Wagner M, Schmitz-Esser S. 2013. Tn*6188* - a novel transposon in *Listeria monocytogenes* responsible for tolerance to benzalkonium chloride. *PLoS One* **8:**e76835.
14. Müller A, Rychli K, Zaiser A, Wieser C, Wagner M, Schmitz-Esser S. 2014. The *Listeria monocytogenes* transposon Tn*6188* provides increased tolerance to various quaternary ammonium compounds and ethidium bromide. *FEMS Microbiol Lett* **361:**166-173.
15. Kremer PH, Lees JA, Koopmans MM, Ferwerda B, Arends AW, Feller MM, Schipper K, Valls Seron M, van der Ende A, Brouwer MC, van de Beek D, Bentley SD. 2017. Benzalkonium tolerance genes and outcome in *Listeria monocytogenes* meningitis. *Clin Microbiol Infect* **23:**265 e261-265 e267.

16. Gilmour MW, Graham M, Van Domselaar G, Tyler S, Kent H, Trout-Yakel KM, Larios O, Allen V, Lee B, Nadon C. 2010. High-throughput genome sequencing of two *Listeria monocytogenes* clinical isolates during a large foodborne outbreak. *BMC Genom* **11:**120.

17. Kovacevic J, Ziegler J, Walecka-Zacharska E, Reimer A, Kitts DD, Gilmour MW. 2016. Tolerance of *Listeria monocytogenes* to quaternary ammonium sanitizers is mediated by a novel efflux pump encoded by *emrE*. *Appl Environ Microbiol* **82:**939-953.

18. Dutta V, Elhanafi D, Kathariou S. 2013. Conservation and distribution of the benzalkonium chloride resistance cassette *bcrABC* in *Listeria monocytogenes*. *Appl Environ Microbiol* **79:**6067-6074.

19. Elhanafi D, Dutta V, Kathariou S. 2010. Genetic characterization of plasmid-associated benzalkonium chloride resistance determinants in a *Listeria monocytogenes* strain from the 1998-1999 outbreak. *Appl Environ Microbiol* **76:**8231-8238.

20. Pirone-Davies C, Chen Y, Pightling A, Ryan G, Wang Y, Yao K, Hoffmann M, Allard MW. 2018. Genes significantly associated with lineage II food isolates of *Listeria monocytogenes*. *BMC Genom* **19:**708.

21. Fagerlund A, Langsrud S, Møretrø T. 2020. In-depth longitudinal study of *Listeria monocytogenes* ST9 isolates from the meat processing industry: Resolving diversity and transmission patterns using whole-genome sequencing. *Appl Environ Microbiol* **86:**e00579-20.

22. Dutta V, Elhanafi D, Osborne J, Martinez MR, Kathariou S. 2014. Genetic characterization of plasmid-associated triphenylmethane reductase in *Listeria monocytogenes*. *Appl Environ Microbiol* **80:**5379-5385.

23. Jiang X, Ren S, Geng Y, Yu T, Li Y, Liu L, Liu G, Wang H, Shi L. 2020. The *sug* operon involves in resistance to quaternary ammonium compounds in *Listeria monocytogenes* EGD-e. *Appl Microbiol Biotechnol* **104:**7093-7104.

24. Jiang X, Yu T, Xu Y, Wang H, Korkeala H, Shi L. 2019. MdrL, a major facilitator superfamily efflux pump of *Listeria monocytogenes* involved in tolerance to benzalkonium chloride. Appl Microbiol Biotechnol 103:1339-1350.

25. Lim SY, Yap KP, Thong KL. 2016. Comparative genomics analyses revealed two virulent *Listeria monocytogenes* strains isolated from ready-to-eat food. *Gut Pathog* **8:**65.

26. Mata MT, Baquero F, Pérez-Díaz JC. 2000. A multidrug efflux transporter in *Listeria monocytogenes*. *FEMS Microbiol Lett* **187:**185-188.

27. Godreuil S, Galimand M, Gerbaud G, Jacquet C, Courvalin P. 2003. Efflux pump Lde is associated with fluoroquinolone resistance in *Listeria monocytogenes*. *Antimicrob Agents Chemother* **47:**704-708.

28. Conficoni D, Losasso C, Cortini E, Di Cesare A, Cibin V, Giaccone V, Corno G, Ricci A. 2016. Resistance to biocides in *Listeria monocytogenes* collected in meat-processing environments. *Front Microbiol* **7:**1627.

29. Bland R, Waite-Cusic J, Weisberg AJ, Riutta ER, Chang JH, Kovacevic J. 2021. Adaptation to a commercial quaternary ammonium compound sanitizer leads to cross-resistance to select antibiotics in *Listeria monocytogenes* isolated from fresh produce environments. *Front Microbiol* **12:**782920.

30. Haubert L, Kremer FS, da Silva WP. 2018. Whole-genome sequencing identification of a multidrug-resistant *Listeria monocytogenes* serotype 1/2a isolated from fresh mixed sausage in southern Brazil. *Infect Genet Evol* **65:**127-130.

31. Møretrø T, Langsrud S. 2004. *Listeria monocytogenes*: Biofilm formation and persistence in food processing environments. *Biofilms* **1:**107-121.

32. Giaouris E, Heir E, Desvaux M, Hébraud M, Møretrø T, Langsrud S, Doulgeraki A, Nychas GJ, Kačániová M, Czaczyk K, Ölmez H, Simões M. 2015. Intra- and inter-species interactions within biofilms of important foodborne bacterial pathogens. *Front Microbiol* **6:**841.

33. Giaouris E, Heir E, Hébraud M, Chorianopoulos N, Langsrud S, Møretrø T, Habimana O, Desvaux M, Renier S, Nychas GJ. 2014. Attachment and biofilm formation by foodborne bacteria in meat processing environments: causes, implications, role of bacterial interactions and control by alternative novel methods. *Meat Sci* **97:**298-309.

34. Jordan SJ, Perni S, Glenn S, Fernandes I, Barbosa M, Sol M, Tenreiro RP, Chambel L, Barata B, Zilhao I, Aldsworth TG, Adriao A, Faleiro ML, Shama G, Andrew PW. 2008. *Listeria monocytogenes* biofilm-associated protein (BapL) may contribute to surface attachment of *L. monocytogenes* but is absent from many field isolates. *Appl Environ Microbiol* **74:**5451-5456.

35. Popowska M, Krawczyk-Balska A, Ostrowski R, Desvaux M. 2017. InlL from *Listeria monocytogenes* is involved in biofilm formation and adhesion to mucin. *Front Microbiol* **8:**660.

36. Bierne H, Sabet C, Personnic N, Cossart P. 2007. Internalins: a complex family of leucine-rich repeat-containing proteins in *Listeria monocytogenes*. *Microbes Infect* **9:**1156-1166.

37. Wagner E. 2019. The role of hypervariable genetic hotspots in stress response and virulence of *Listeria monocytogenes*. PhD thesis. University of Veterinary Medicine Vienna. https://www.vetmeduni.ac.at/hochschulschriften/phds/AC15733057.pdf

38. Ryan S, Begley M, Hill C, Gahan CG. 2010. A five-gene stress survival islet (SSI-1) that contributes to the growth of *Listeria monocytogenes* in suboptimal conditions. *J Appl Microbiol* **109:**984-995.

39. Harter E, Wagner EM, Zaiser A, Halecker S, Wagner M, Rychli K. 2017. Stress Survival Islet 2, predominantly present in *Listeria monocytogenes* strains of sequence type 121, is involved in the alkaline and oxidative stress responses. *Appl Environ Microbiol* **83:**e00827-17.

40. Assisi C, Forauer E, Oliver HF, Etter AJ. 2021. Genomic and transcriptomic analysis of biofilm formation in persistent and transient *Listeria monocytogenes* isolates from the retail deli environment does not yield insight into persistence mechanisms. *Foodborne Pathog Dis* **18:**179-188.

41. Keeney K, Trmcic A, Zhu Z, Delaquis P, Wang S. 2018. Stress survival islet 1 contributes to serotype-specific differences in biofilm formation in *Listeria monocytogenes*. *Lett Appl Microbiol* **67:**530-536.

42. Mahoney DBJ, Falardeau J, Hingston P, Chmielowska C, Carroll LM, Wiedmann M, Jang SS, Wang S. 2022. Associations between *Listeria monocytogenes* genomic characteristics and adhesion to polystyrene at 8 °C. *Food Microbiol* **102:**103915.

43. Upham J, Chen S, Boutilier E, Hodges L, Eisebraun M, Croxen MA, Fortuna A, Mallo GV, Garduño RA. 2019. Potential ad hoc markers of persistence and virulence in Canadian *Listeria monocytogenes* food and clinical isolates. *J Food Prot* **82:**1909-1921.

44. Drolia R, Bhunia AK. 2019. Crossing the intestinal barrier via *Listeria* adhesion protein and internalin A. *Trends Microbiol* **27:**408-425.

45. Ireton K, Mortuza R, Gyanwali GC, Gianfelice A, Hussain M. 2021. Role of internalin proteins in the pathogenesis of *Listeria monocytogenes*. *Mol Microbiol* **116:**1407-1419.

46. Van Stelten A, Simpson JM, Ward TJ, Nightingale KK. 2010. Revelation by single-nucleotide polymorphism genotyping that mutations leading to a premature stop codon in *inlA* are common among *Listeria monocytogenes* isolates from ready-to-eat foods but not human listeriosis cases. *Appl Environ Microbiol* **76:**2783-2790.

47. Piercey MJ, Hingston PA, Truelstrup Hansen L. 2016. Genes involved in *Listeria monocytogenes* biofilm formation at a simulated food processing plant temperature of 15 °C. *Int J Food Microbiol* **223:**63-74.

48. Orsi RH, Bowen BM, Wiedmann M. 2010. Homopolymeric tracts represent a general regulatory mechanism in prokaryotes. *BMC Genom* **11:**102.

49. Jonquières R, Bierne H, Mengaud J, Cossart P. 1998. The *inlA* gene of *Listeria monocytogenes* LO28 harbors a nonsense mutation resulting in release of internalin. *Infect Immun* **66:**3420-3422.

50. Chen Y, Chen Y, Pouillot R, Dennis S, Xian Z, Luchansky JB, Porto-Fett ACS, Lindsay JA, Hammack TS, Allard M, Van Doren JM, Brown EW. 2020. Genetic diversity and profiles of genes associated with virulence and stress resistance among isolates from the 2010-2013 interagency *Listeria monocytogenes* market basket survey. *PLoS One* **15:**e0231393.

51. Ragon M, Wirth T, Hollandt F, Lavenir R, Lecuit M, Le Monnier A, Brisse S. 2008. A new perspective on *Listeria monocytogenes* evolution. *PLoS Pathog* **4:**e1000146.

52. Kovacevic J, Arguedas-Villa C, Wozniak A, Tasara T, Allen KJ. 2013. Examination of food chain-derived *Listeria monocytogenes* strains of different serotypes reveals considerable diversity in *inlA* genotypes, mutability, and adaptation to cold temperatures. *Appl Environ Microbiol* **79:**1915-1922.

53. Camargo AC, Moura A, Avillan J, Herman N, McFarland AP, Sreevatsan S, Call DR, Woodward JJ, Lecuit M, Nero LA. 2019. Whole-genome sequencing reveals *Listeria monocytogenes* diversity and allows identification of long-term persistent strains in Brazil. *Environ Microbiol* **21:**4478-4487.

54. Bleymüller WM, Lammermann N, Ebbes M, Maynard D, Geerds C, Niemann HH. 2016. MET-activating residues in the B-repeat of the *Listeria monocytogenes* invasion protein InlB. *J Biol Chem* **291:**25567-25577.

55. Ebbes M, Bleymüller WM, Cernescu M, Nolker R, Brutschy B, Niemann HH. 2011. Fold and function of the InlB B-repeat. *J Biol Chem* **286:**15496-15506.

56. Geerds C, Bleymüller WM, Meyer T, Widmann C, Niemann HH. 2022. A recurring packing contact in crystals of InlB pinpoints functional binding sites in the internalin domain and the B repeat. *Acta Crystallogr D Struct Biol 78:*310-320.

57. Kurpas M, Osek J, Moura A, Leclercq A, Lecuit M, Wieczorek K. 2020. Genomic characterization of *Listeria monocytogenes* isolated from ready-to-eat meat and meat processing environments in Poland. *Front Microbiol* **11:**1412.

58. Hurley D, Luque-Sastre L, Parker CT, Huynh S, Eshwar AK, Nguyen SV, Andrews N, Moura A, Fox EM, Jordan K, Lehner A, Stephan R, Fanning S. 2019. Whole-genome sequencing-based characterization of 100 *Listeria monocytogenes* isolates collected from food processing environments over a four-year period. *mSphere* **4:**e00252-19.

59. Fagerlund A, Langsrud S, Schirmer BCT, Møretrø T, Heir E. 2016. Genome analysis of *Listeria monocytogenes* sequence type 8 strains persisting in salmon and poultry processing environments and comparison with related strains. *PLoS One* **11:**e0151117.

60. Van Walle I, Björkman JT, Cormican M, Dallman T, Mossong J, Moura A, Pietzka A, Ruppitsch W, Takkinen J, European Listeria WGS Typing Group. 2018. Retrospective validation of whole genome sequencing-enhanced surveillance of listeriosis in Europe, 2010 to 2015. *Euro Surveill* **23:**1700798.

61. Fagerlund A, Idland L, Heir E, Møretrø T, Aspholm M, Lindbäck T, Langsrud S. 2022. WGS analysis of *Listeria monocytogenes* from rural, urban, and farm environments in Norway: Genetic diversity, persistence, and relation to clinical and food isolates. *Appl Environ Microbiol* **88:**e02136-21.

62. Cheng C, Dong Z, Han X, Sun J, Wang H, Jiang L, Yang Y, Ma T, Chen Z, Yu J, Fang W, Song H. 2017. *Listeria monocytogenes* 10403S arginine repressor ArgR finely tunes arginine metabolism regulation under acidic conditions. *Front Microbiol* **8:**145.

63. Sleator RD, Gahan CG, Abee T, Hill C. 1999. Identification and disruption of BetL, a secondary glycine betaine transport system linked to the salt tolerance of *Listeria monocytogenes* LO28. *Appl Environ Microbiol* **65:**2078-2083.

64. Mullapudi S, Siletzky RM, Kathariou S. 2010. Diverse cadmium resistance determinants in *Listeria monocytogenes* isolates from the turkey processing plant environment. *Appl Environ Microbiol* **76:**627-630.

65. Pöntinen A, Aalto-Araneda M, Lindström M, Korkeala H. 2017. Heat resistance mediated by pLM58 plasmid-borne ClpL in *Listeria monocytogenes*. *mSphere* **2:**e00364-17.

66. Nair S, Derre I, Msadek T, Gaillot O, Berche P. 2000. CtsR controls class III heat shock gene expression in the human pathogen *Listeria monocytogenes*. *Mol Microbiol* **35:**800-811.

67. Balogh D, Dahmen M, Stahl M, Poreba M, Gersch M, Drag M, Sieber S. 2017. Insights into ClpXP proteolysis: heterooligomerization and partial deactivation enhance chaperone affinity and substrate turnover in *Listeria monocytogenes*. *Chem Sci* **8:**1592-1600.

68. Schmid B, Klumpp J, Raimann E, Loessner MJ, Stephan R, Tasara T. 2009. Role of cold shock proteins in growth of *Listeria monocytogenes* under cold and osmotic stress conditions. *Appl Environ Microbiol* **75:**1621-1627.

69. Hanawa T, Fukuda M, Kawakami H, Hirano H, Kamiya S, Yamamoto T. 1999. The *Listeria monocytogenes* DnaK chaperone is required for stress tolerance and efficient phagocytosis with macrophages. *Cell Stress Chaperones* **4:**118-128.

70. Zankari E, Hasman H, Cosentino S, Vestergaard M, Rasmussen S, Lund O, Aarestrup FM, Larsen MV. 2012. Identification of acquired antimicrobial resistance genes. *J Antimicrob Chemother* **67:**2640-2644.

71. Fillgrove KL, Pakhomova S, Newcomer ME, Armstrong RN. 2003. Mechanistic diversity of fosfomycin resistance in pathogenic microorganisms. *J Am Chem Soc* **125:**15730-15731.

72. Rea RB, Gahan CG, Hill C. 2004. Disruption of putative regulatory loci in *Listeria monocytogenes* demonstrates a significant role for Fur and PerR in virulence. *Infect Immun* **72:**717-727.

73. Cotter PD, Gahan CG, Hill C. 2001. A glutamate decarboxylase system protects *Listeria monocytogenes i*n gastric fluid. *Mol Microbiol* **40:**465-475.

74. Chen J, Fang C, Zheng T, Zhu N, Bei Y, Fang W. 2012. Genomic presence of *gadD1* glutamate decarboxylase correlates with the organization of *ascB-dapE* internalin cluster in *Listeria monocytogenes*. *Foodborne Pathog Dis* **9:**175-178.

75. Ko R, Smith LT. 1999. Identification of an ATP-driven, osmoregulated glycine betaine transport system in *Listeria monocytogenes*. *Appl Environ Microbiol* **65:**4040-4048.

76. Mendum ML, Smith LT. 2002. Characterization of glycine betaine porter I from *Listeria monocytogenes* and its roles in salt and chill tolerance. *Appl Environ Microbiol* **68:**813-819.

77. Hingston P, Brenner T, Truelstrup Hansen L, Wang S. 2019. Comparative analysis of *Listeria monocytogenes* plasmids and expression levels of plasmid-encoded genes during growth under salt and acid stress conditions. *Toxins (Basel)* **11:**426.

78. Gahan CG, O'Mahony J, Hill C. 2001. Characterization of the *groESL* operon in *Listeria monocytogenes*: utilization of two reporter systems (*gfp* and *hly*) for evaluating in vivo expression. *Infect Immun* **69:**3924-3932.

79. Durack J, Ross T, Bowman JP. 2013. Characterisation of the transcriptomes of genetically diverse *Listeria monocytogenes* exposed to hyperosmotic and low temperature conditions reveal global stress-adaptation mechanisms. *PLoS One* **8:**e73603.

80. Chaturongakul S, Raengpradub S, Wiedmann M, Boor KJ. 2008. Modulation of stress and virulence in *Listeria monocytogenes*. *Trends Microbiol* **16:**388-396.

81. Cotter PD, Emerson N, Gahan CG, Hill C. 1999. Identification and disruption of *lisRK*, a genetic locus encoding a two-component signal transduction system involved in stress tolerance and virulence in *Listeria monocytogenes*. *J Bacteriol* **181:**6840-6843.

82. Sue D, Fink D, Wiedmann M, Boor KJ. 2004. σ^B-dependent gene induction and expression in *Listeria monocytogenes* during osmotic and acid stress conditions simulating the intestinal environment. *Microbiology (Reading)* **150:**3843-3855.

83. Ondrusch N, Kreft J. 2011. Blue and red light modulates SigB-dependent gene transcription, swimming motility and invasiveness in *Listeria monocytogenes*. *PLoS One* **6:**e16151.

84. Hain T, Hossain H, Chatterjee SS, Machata S, Volk U, Wagner S, Brors B, Haas S, Kuenne CT, Billion A, Otten S, Pane-Farre J, Engelmann S, Chakraborty T. 2008. Temporal transcriptomic analysis of the *Listeria monocytogenes* EGD-e σ^B regulon. *BMC Microbiol* **8:**20.

85. Hu Y, Oliver HF, Raengpradub S, Palmer ME, Orsi RH, Wiedmann M, Boor KJ. 2007. Transcriptomic and phenotypic analyses suggest a network between the transcriptional regulators HrcA and σ^B in *Listeria monocytogenes*. *Appl Environ Microbiol* **73:**7981-7991.

86. Raengpradub S, Wiedmann M, Boor KJ. 2008. Comparative analysis of the σ^B-dependent stress responses in *Listeria monocytogenes* and *Listeria innocua* strains exposed to selected stress conditions. *Appl Environ Microbiol* **74:**158-171.

87. Hein I, Klinger S, Dooms M, Flekna G, Stessl B, Leclercq A, Hill C, Allerberger F, Wagner M. 2011. Stress survival islet 1 (SSI-1) survey in *Listeria monocytogenes* reveals an insert common to *Listeria innocua* in sequence type 121 *L. monocytogenes* strains. *Appl Environ Microbiol* **77:**2169-2173.

88. Zheng W, Kathariou S. 1995. Differentiation of epidemic-associated strains of *Listeria monocytogenes* by restriction fragment length polymorphism in a gene region essential for growth at low temperatures (4°C). *Appl Environ Microbiol* **61:**4310-4314.

89. Sitthisak S, Howieson K, Amezola C, Jayaswal RK. 2005. Characterization of a multicopper oxidase gene from *Staphylococcus aureus*. *Appl Environ Microbiol* **71:**5650-5653.

90. Cortes BW, Naditz AL, Anast JM, Schmitz-Esser S. 2020. Transcriptome sequencing of *Listeria monocytogenes* reveals major gene expression changes in response to lactic acid stress exposure but a less pronounced response to oxidative stress. *Front Microbiol* **10:**3110.

91. Borezee E, Pellegrini E, Berche P. 2000. OppA of *Listeria monocytogenes*, an oligopeptide-binding protein required for bacterial growth at low temperature and involved in intracellular survival. *Infect Immun* **68:**7069-7077.

92. Fraser KR, Harvie D, Coote PJ, O'Byrne CP. 2000. Identification and characterization of an ATP binding cassette L-carnitine transporter in *Listeria monocytogenes*. *Appl Environ Microbiol* **66:**4696-4704.

93. Rea R, Hill C, Gahan CG. 2005. *Listeria monocytogenes* PerR mutants display a small-colony phenotype, increased sensitivity to hydrogen peroxide, and significantly reduced murine virulence. *Appl Environ Microbiol* **71:**8314-8322.

94. Ferreira A, Gray M, Wiedmann M, Boor KJ. 2004. Comparative genomic analysis of the *sigB* operon in *Listeria monocytogenes* and in other Gram-positive bacteria. *Curr Microbiol* **48:**39-46.

95. Chaturongakul S, Boor KJ. 2004. RsbT and RsbV contribute to σ^B-dependent survival under environmental, energy, and intracellular stress conditions in *Listeria monocytogenes*. *Appl Environ Microbiol* **70:**5349-5356.

96. Kazmierczak MJ, Mithoe SC, Boor KJ, Wiedmann M. 2003. *Listeria monocytogenes* σ^B regulates stress response and virulence functions. *J Bacteriol* **185:**5722-5734.

97. Raimann E, Schmid B, Stephan R, Tasara T. 2009. The alternative sigma factor σ^L of *L. monocytogenes* promotes growth under diverse environmental stresses. *Foodborne Pathog Dis* **6:**583-591.

98. Teatero S, Ramoutar E, McGeer A, Li A, Melano RG, Wasserscheid J, Dewar K, Fittipaldi N. 2016. Clonal Complex 17 Group B *Streptococcus* strains causing invasive disease in neonates and adults originate from the same genetic pool. *Sci Rep* **6:**20047.

99. Wilson A, Gray J, Chandry PS, Fox EM. 2018. Phenotypic and genotypic analysis of antimicrobial resistance among *Listeria monocytogenes* isolated from Australian food production chains. *Genes (Basel)* **9:**80.

100. Ramos JL, Martínez-Bueno M, Molina-Henares AJ, Terán W, Watanabe K, Zhang X, Gallegos MT, Brennan R, Tobes R. 2005. The TetR family of transcriptional repressors. *Microbiol Mol Biol Rev* **69:**326-356.

101. Seifart Gomes C, Izar B, Pazan F, Mohamed W, Mraheil MA, Mukherjee K, Billion A, Aharonowitz Y, Chakraborty T, Hain T. 2011. Universal stress proteins are important for oxidative and acid stress resistance and growth of *Listeria monocytogenes* EGD-e *in vitro* and *in vivo*. *PLoS One* **6:**e24965.
